# Supplementary material for: In vivo MRI is sensitive to remyelination in a nonhuman primate model of multiple sclerosis
Source: eLife. 2023 Apr 21;12:e73786. doi: 10.7554/eLife.73786 (PMC10171859; doi:10.7554/eLife.73786)
Supplement: Supplementary file 1. — For each of the immunohistochemical targets, respective source, clonalities and hosts, and methods for antigen retrieval, blocking, and primary and secondary antibody inoculation are listed. HIER = heat-induced epitope retrieval; RT = room temperature; P=polyclonal antibody; M=monoclonal antibody; Rb = rabbit; Ms = mouse [file elife-73786-supp1.docx]

|  | **Company** | **Host / Clonality** | **Antigen Retrieval Method** | **Protein Blocking Reagent** | **Primary Antibody Concentration** | **Primary Antibody Inoculation** | **Secondary Antibody** |
| --- | --- | --- | --- | --- | --- | --- | --- |
| *Iba1* | Wako | Rb, P | HIER with Citrate Buffer, 20" | Protein Block (Dako), 20" for HRP; 2.5% Horse Serum (Vector) for AP | 1:400 | 1 hour, RT | Powervision Poly-HRP or ImmPRESS AP |
| *PLP* | BioRad | Ms, M | HIER with Citrate Buffer, 20" | Protein Block (Dako), 20" | 1:200 | 1 hour, RT | Powervision Poly-HRP |
| *ASPA* | GeneTex | Rb, P | HIER with Citrate Buffer, 45" | Protein Block (Dako), 20" | 1:1500 | 1 hour, RT | Powervision Poly-HRP |
| *Olig2* | EMD Millipore | Rb, P | None when double-staining | 2.5% Horse Serum (Vector), 20" | 1:200 | 1 hour, RT | ImmPRESS AP |
