# Supplementary material for: In vivo MRI is sensitive to remyelination in a nonhuman primate model of multiple sclerosis
Source: eLife. 2023 Apr 21;12:e73786. doi: 10.7554/eLife.73786 (PMC10171859; doi:10.7554/eLife.73786)

IBA1 and PLP IHC Raw Data

- All in x200 zoom using the same microscope
- Left: IBA1
- Right: PLP

M#2

- 1 Chronic Demyelinated
- 9 Remyelinated

Chronic Demyelinated

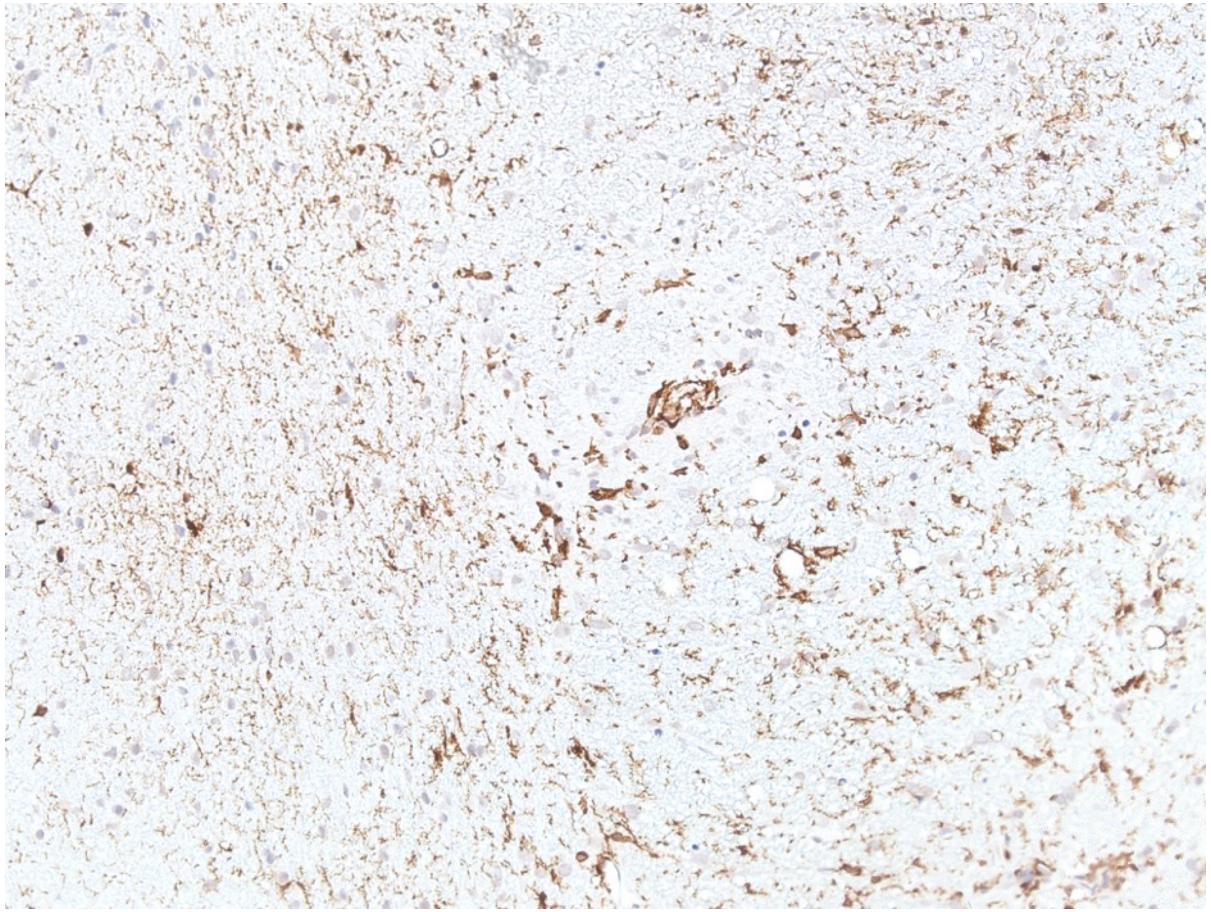

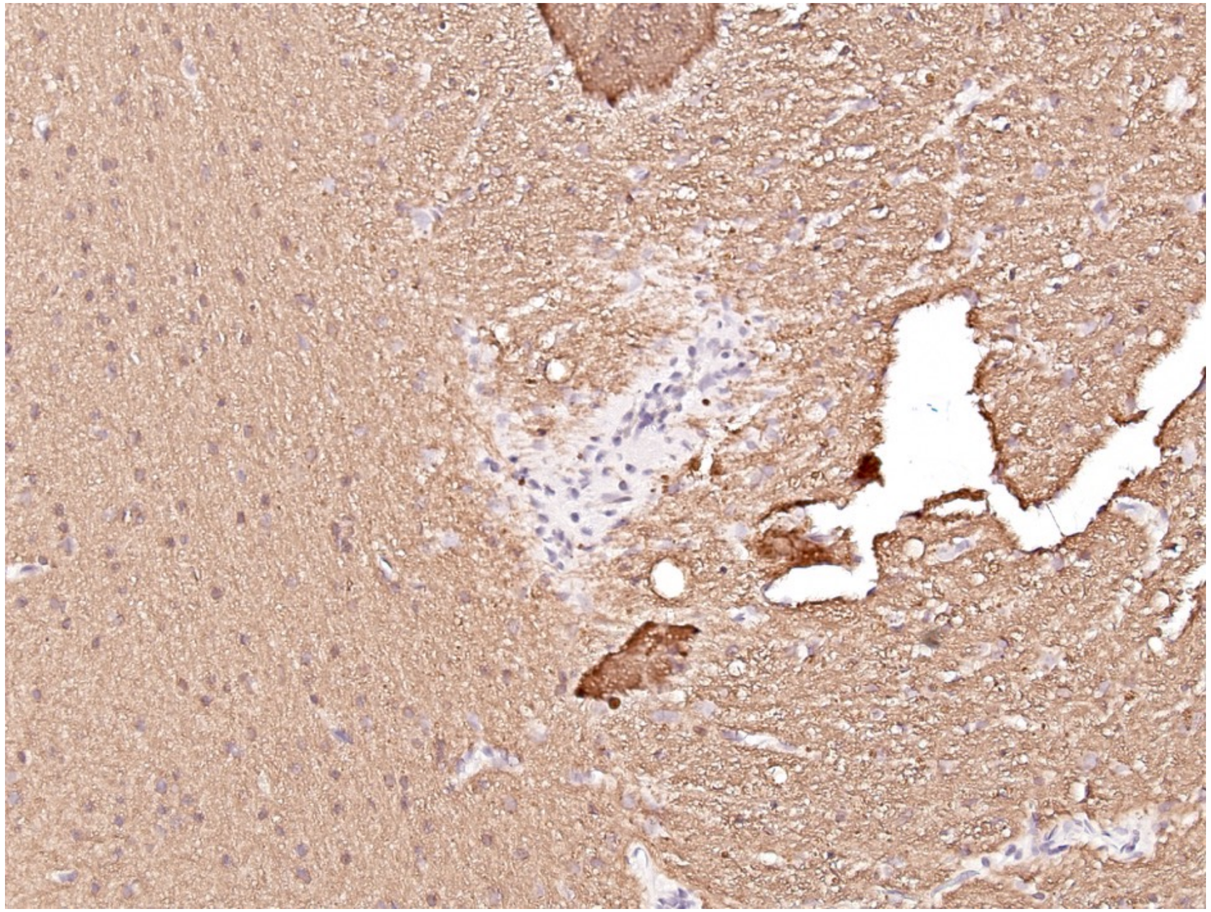

Remyelinated

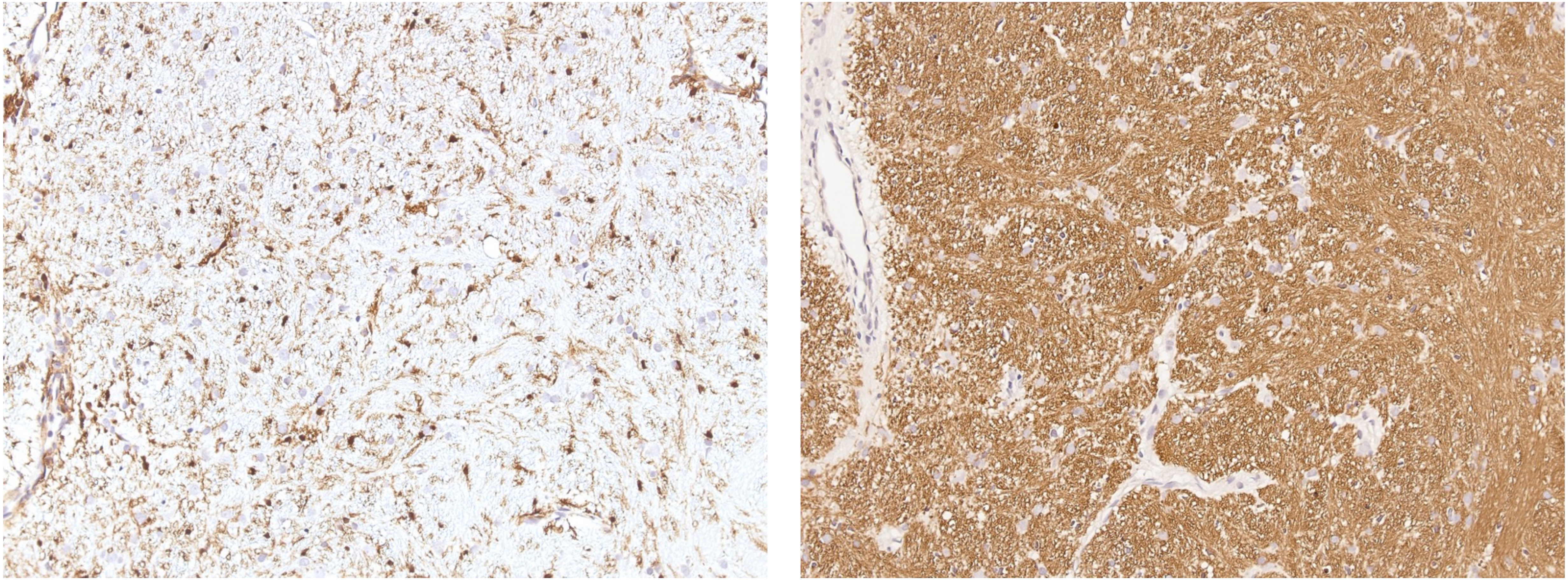

Remyelinated

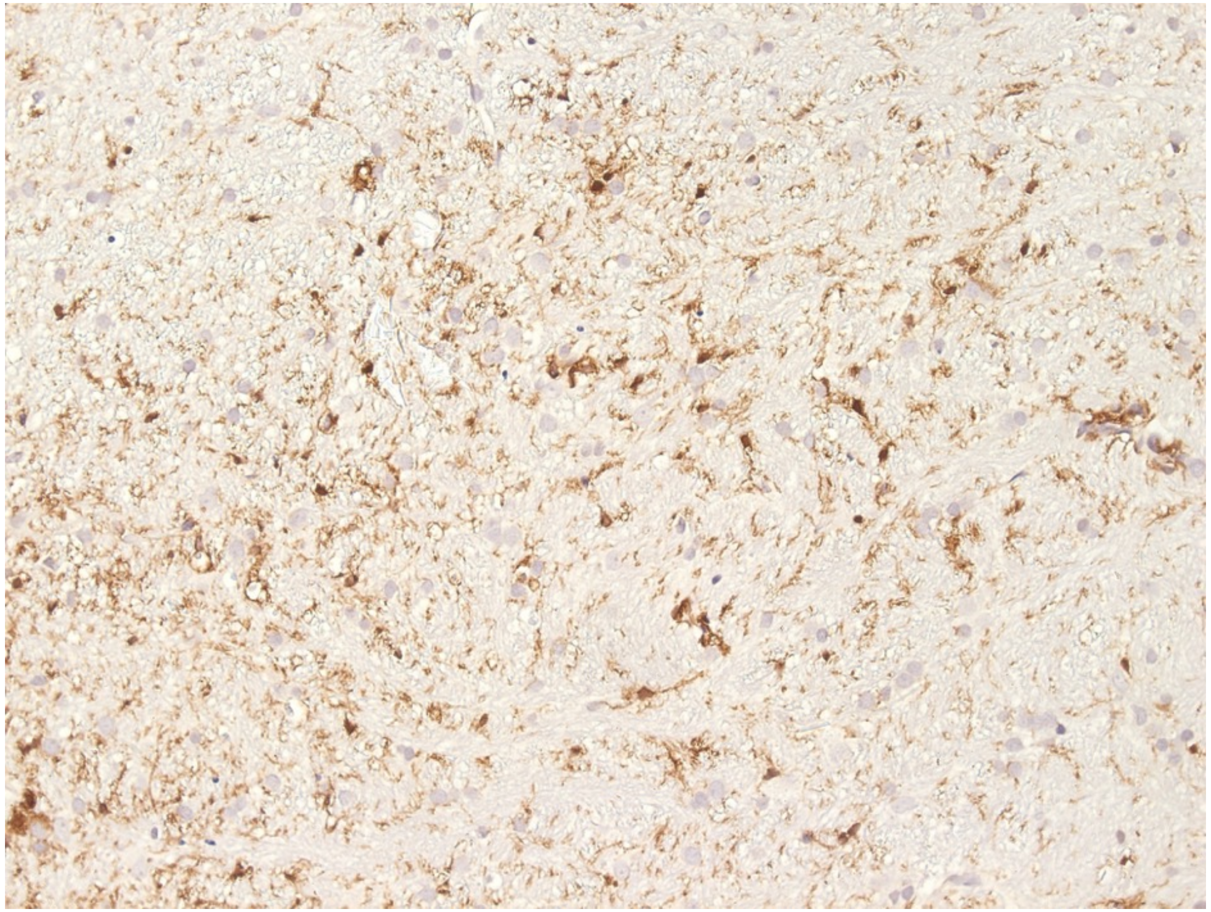

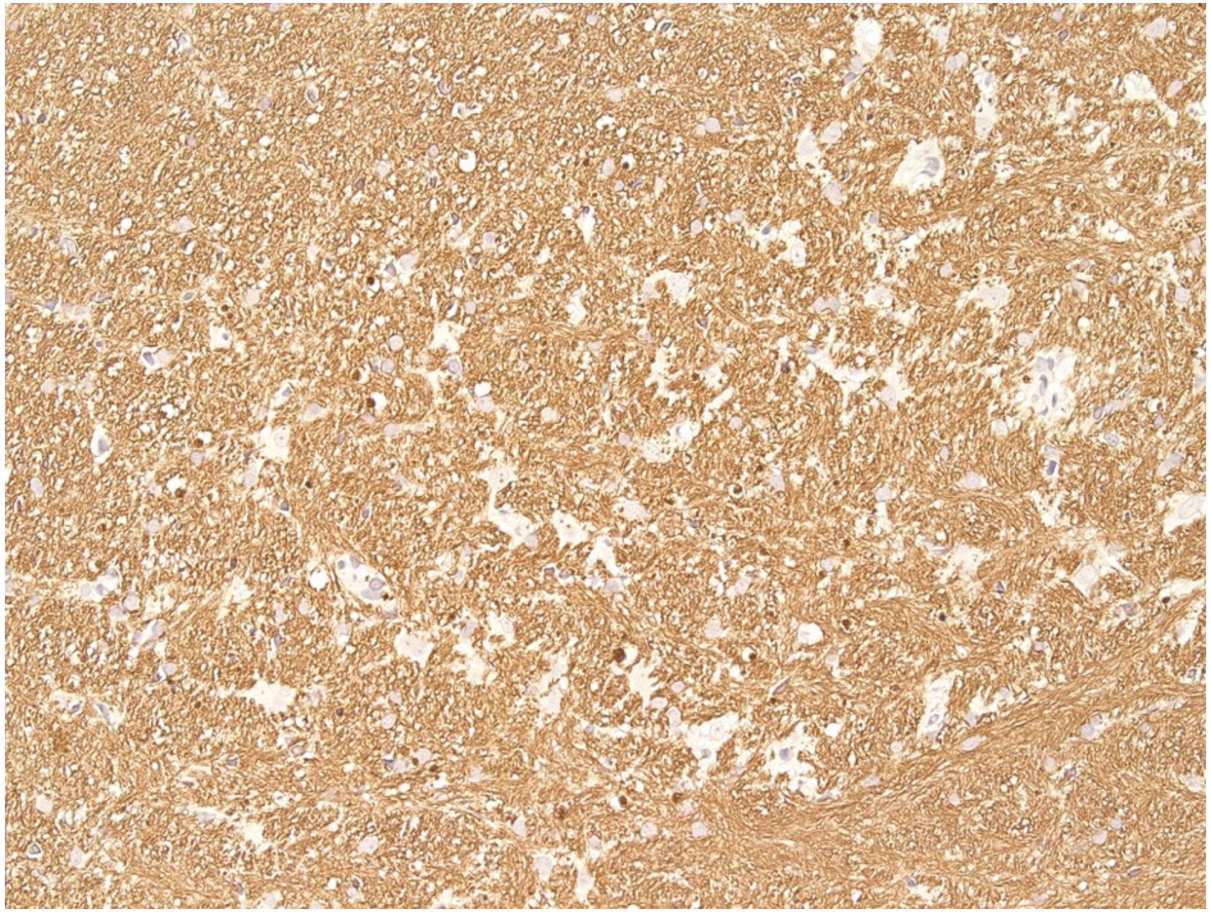

Remyelinated

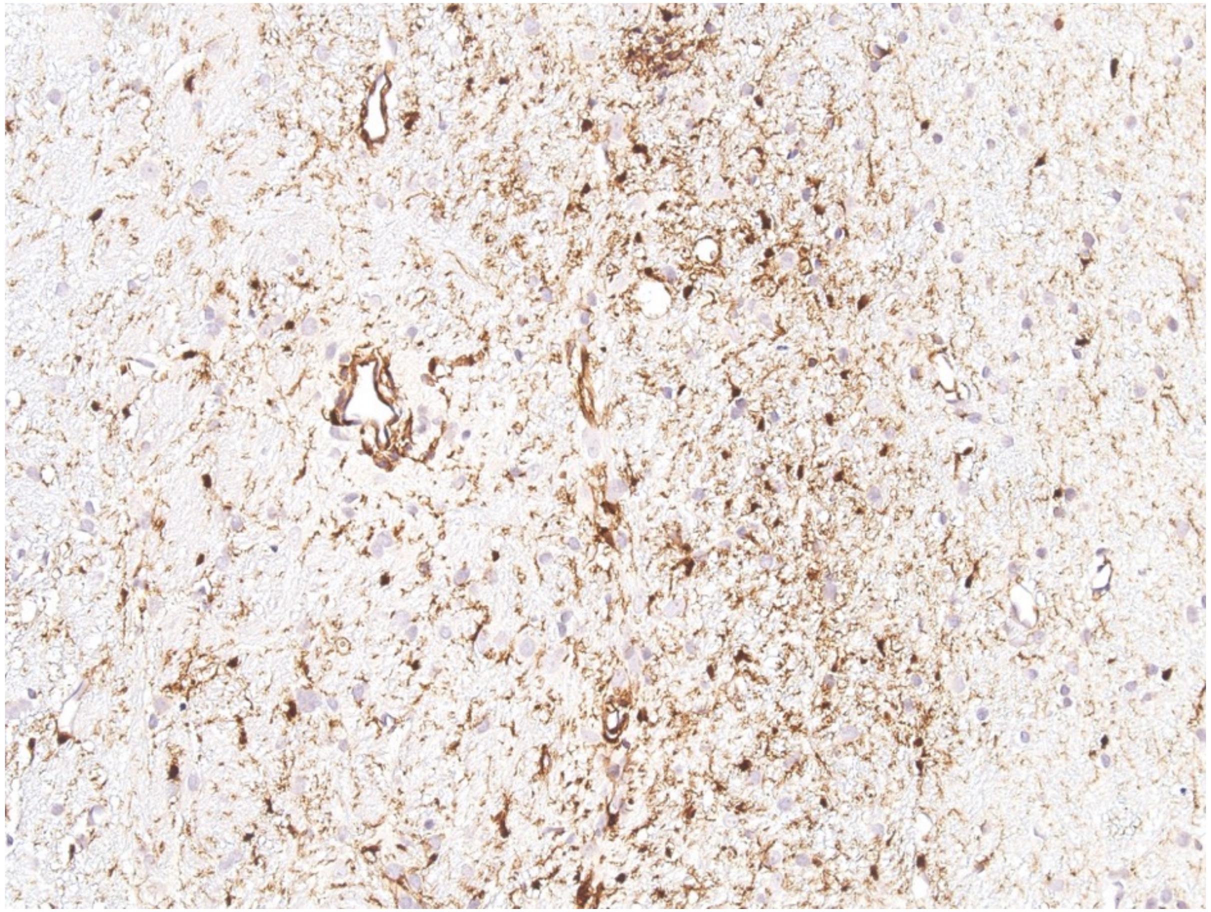

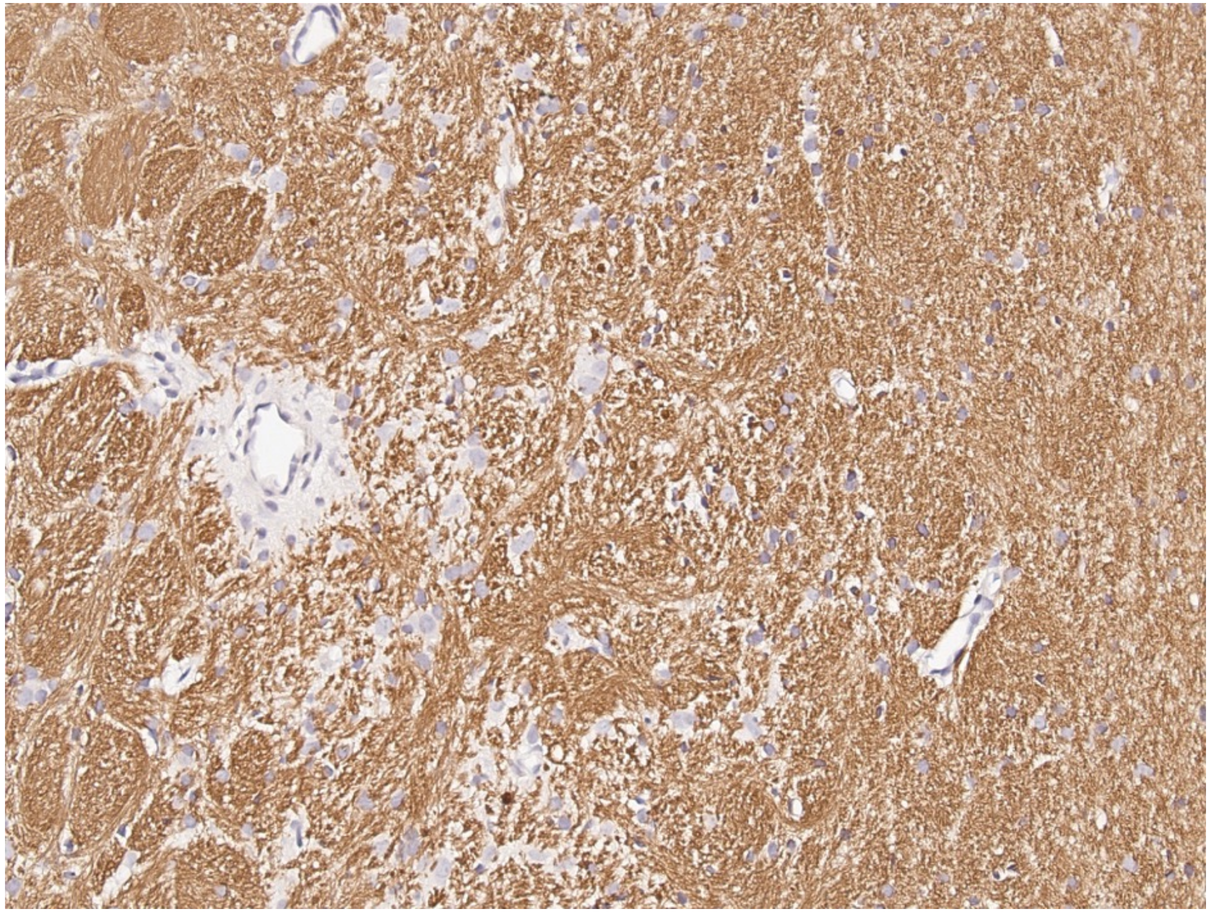

Remyelinated

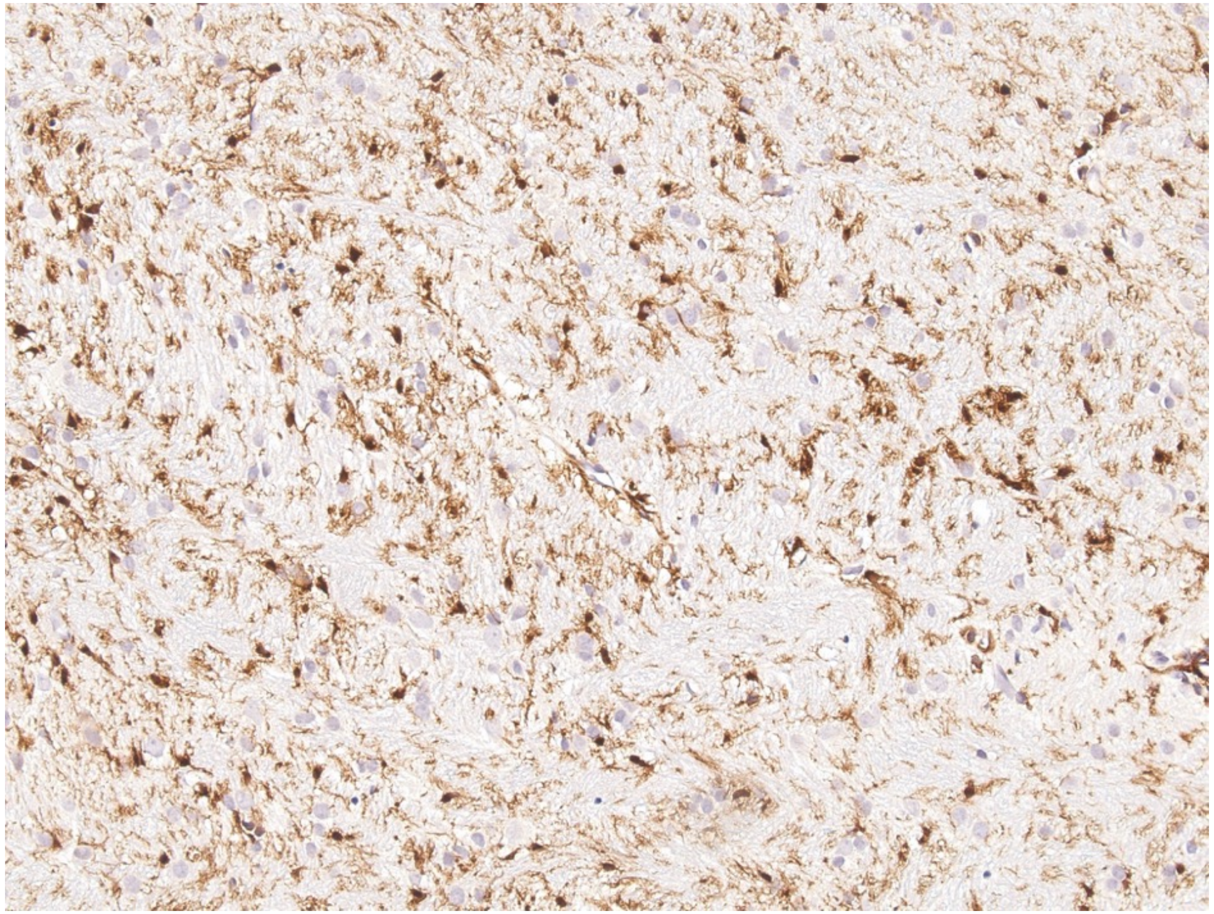

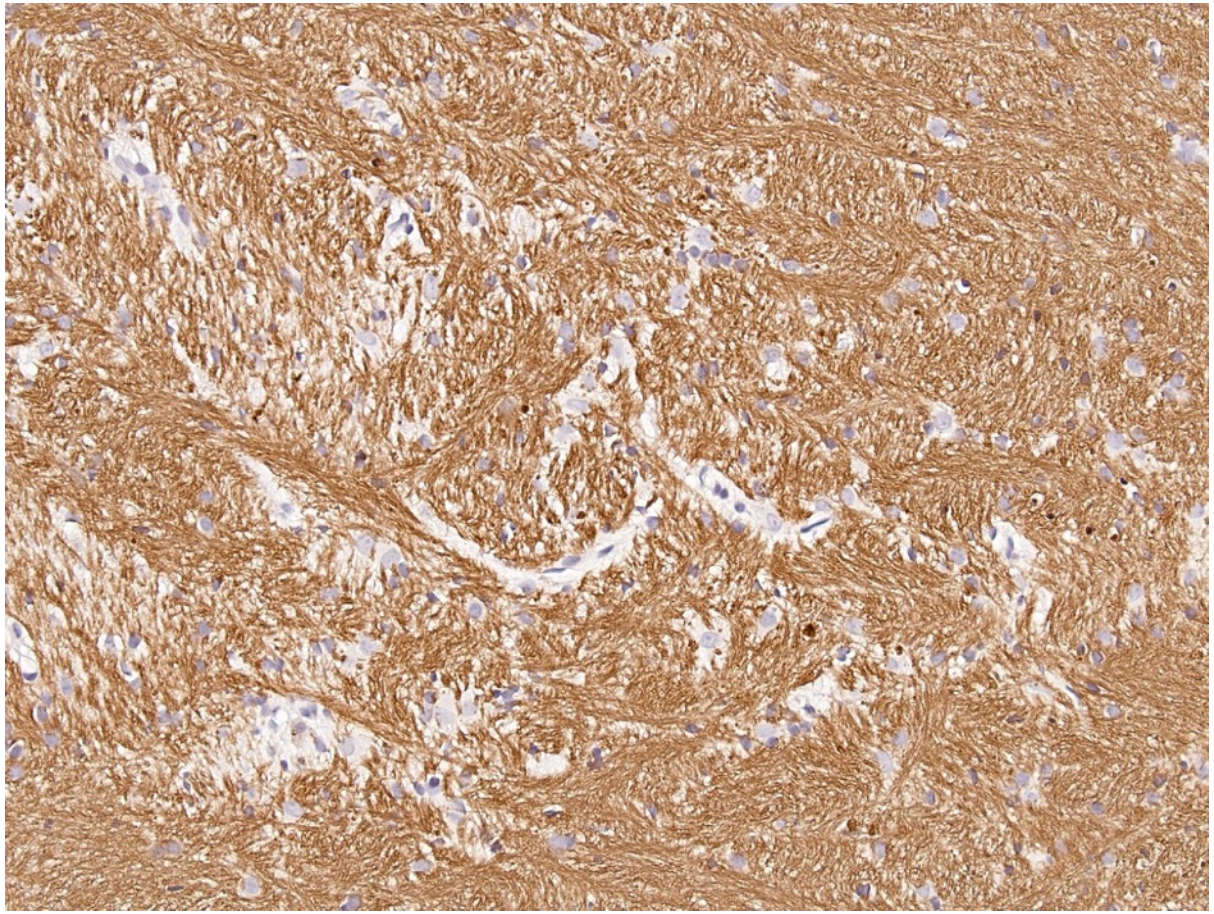

Remyelinated

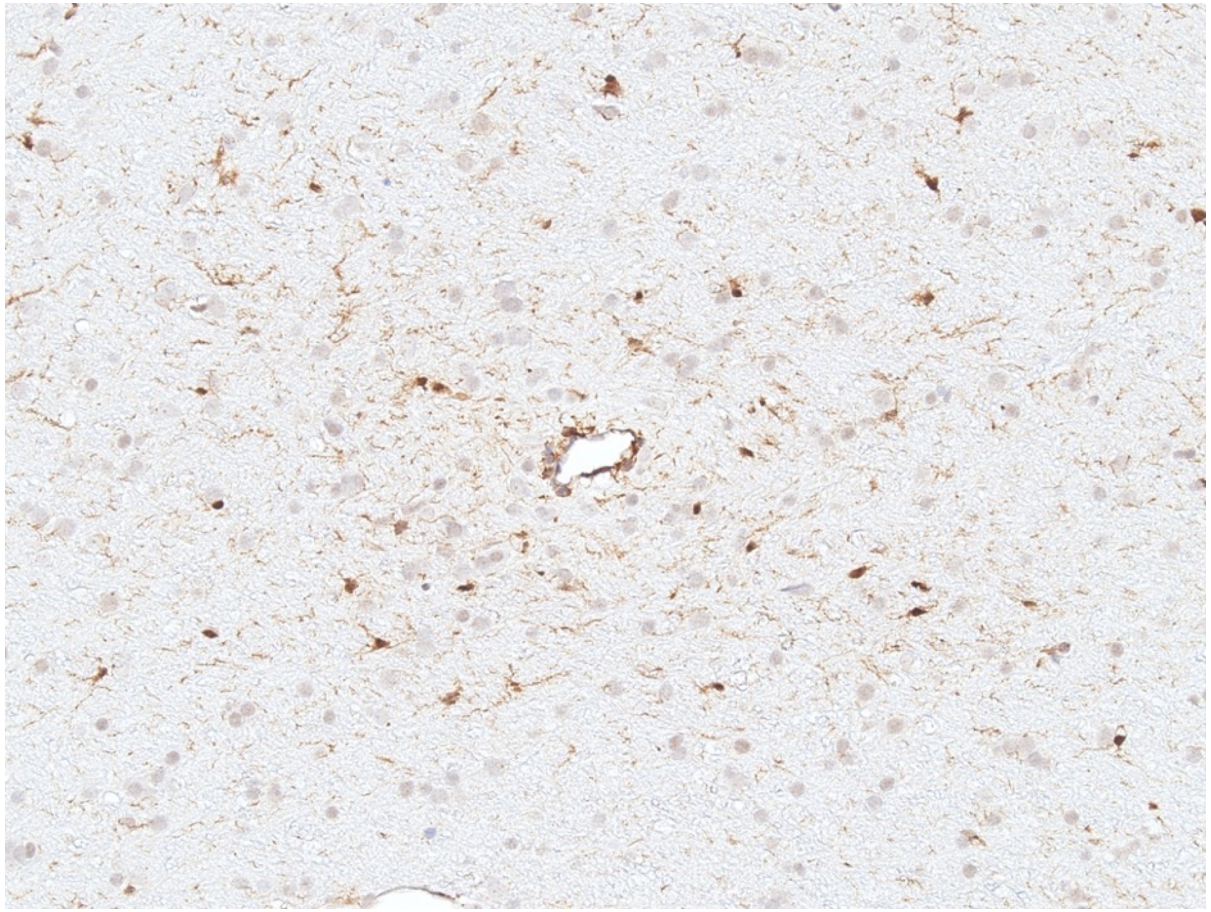

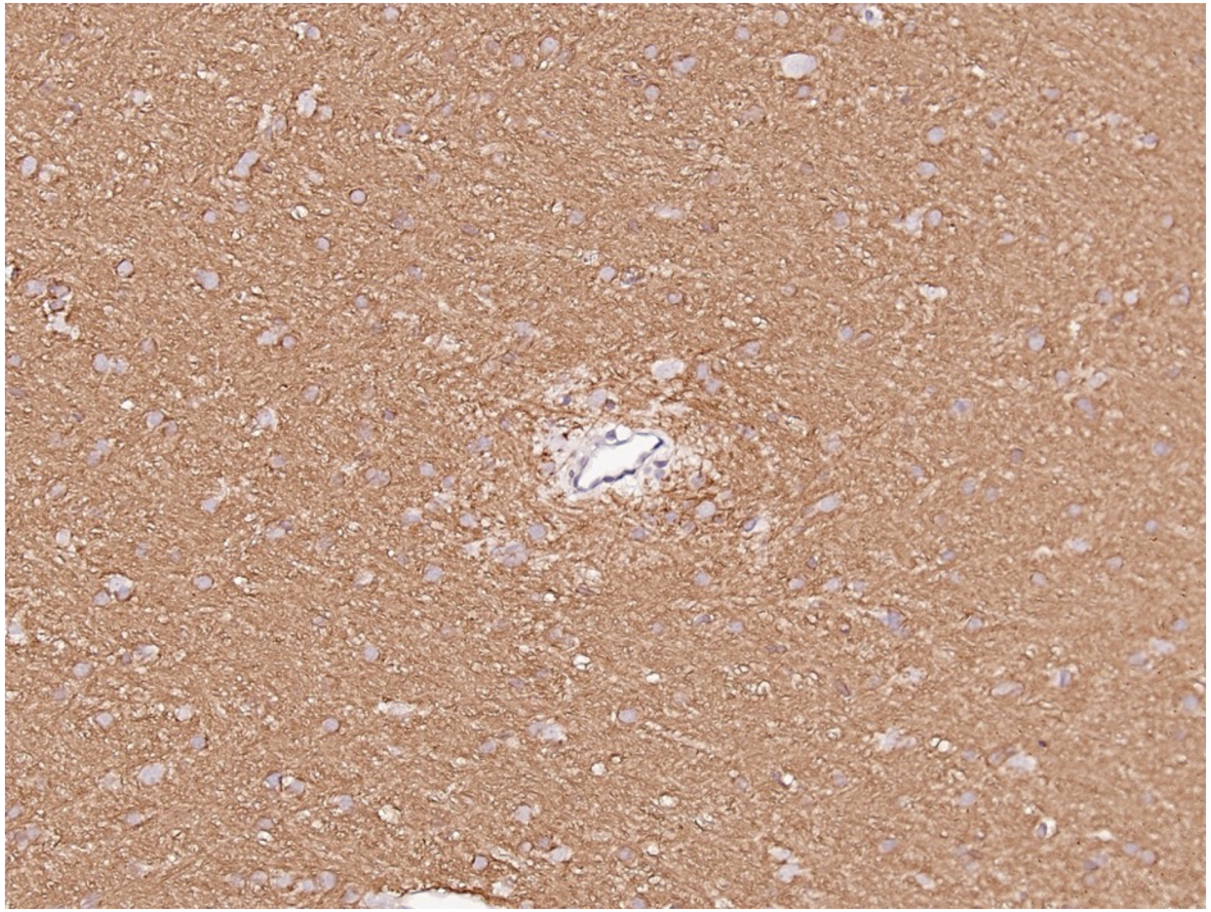

Remyelinated

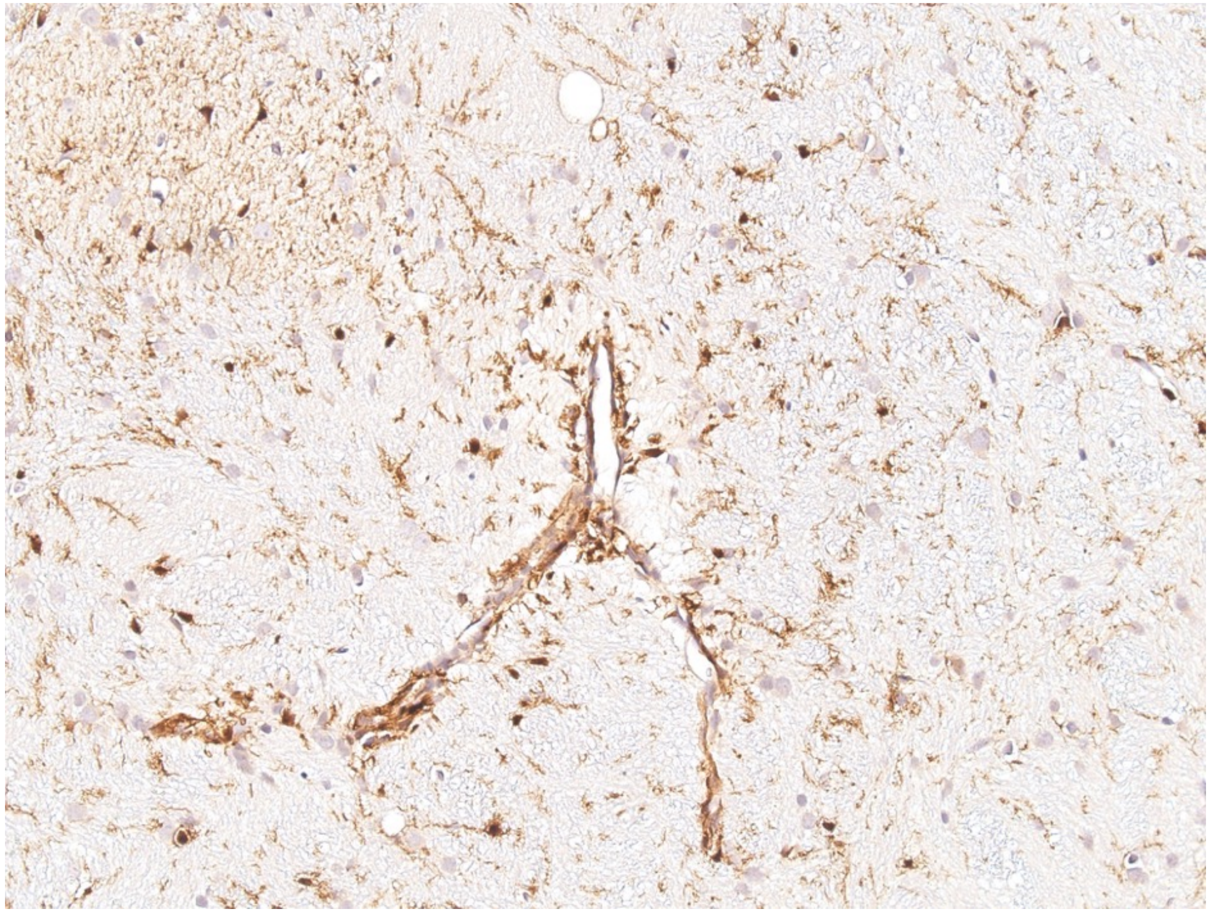

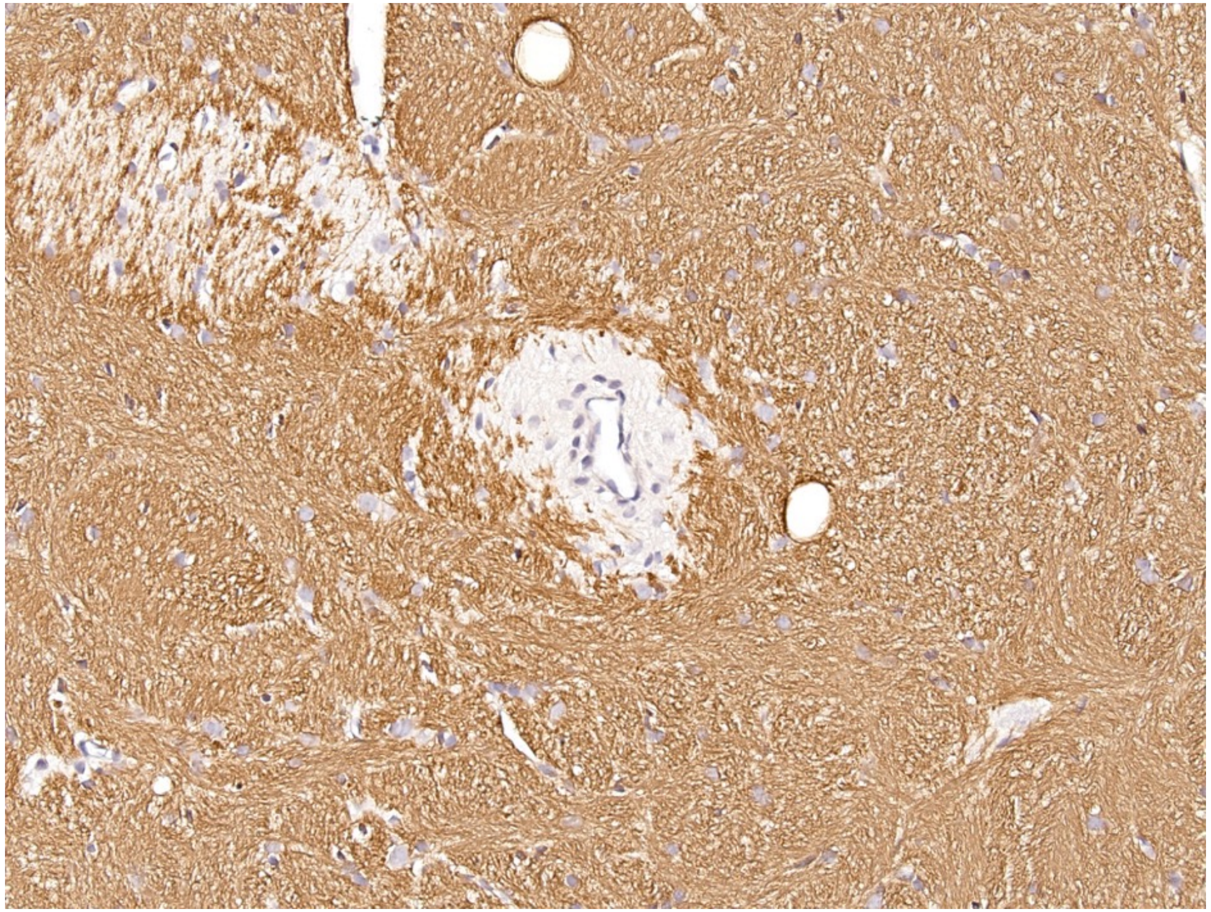

Remyelinated

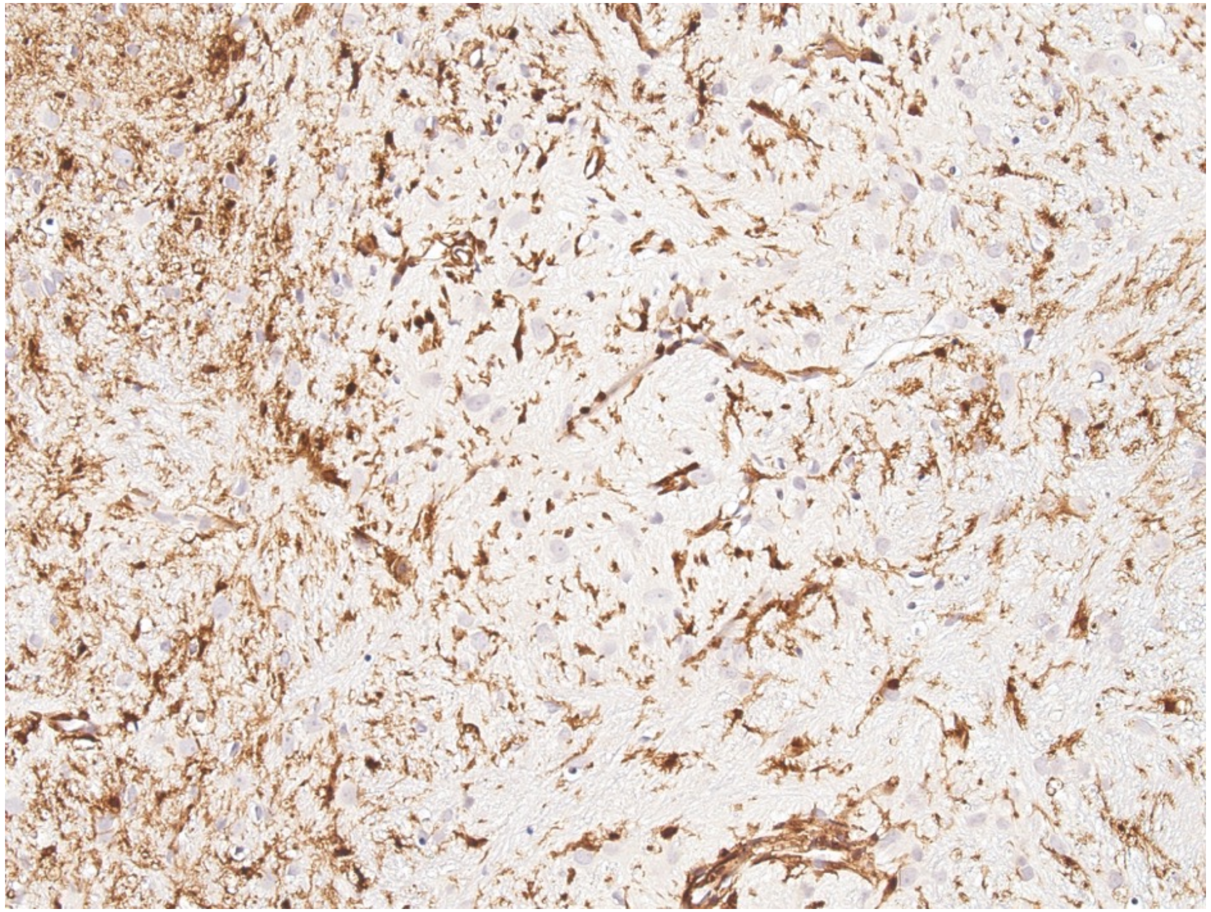

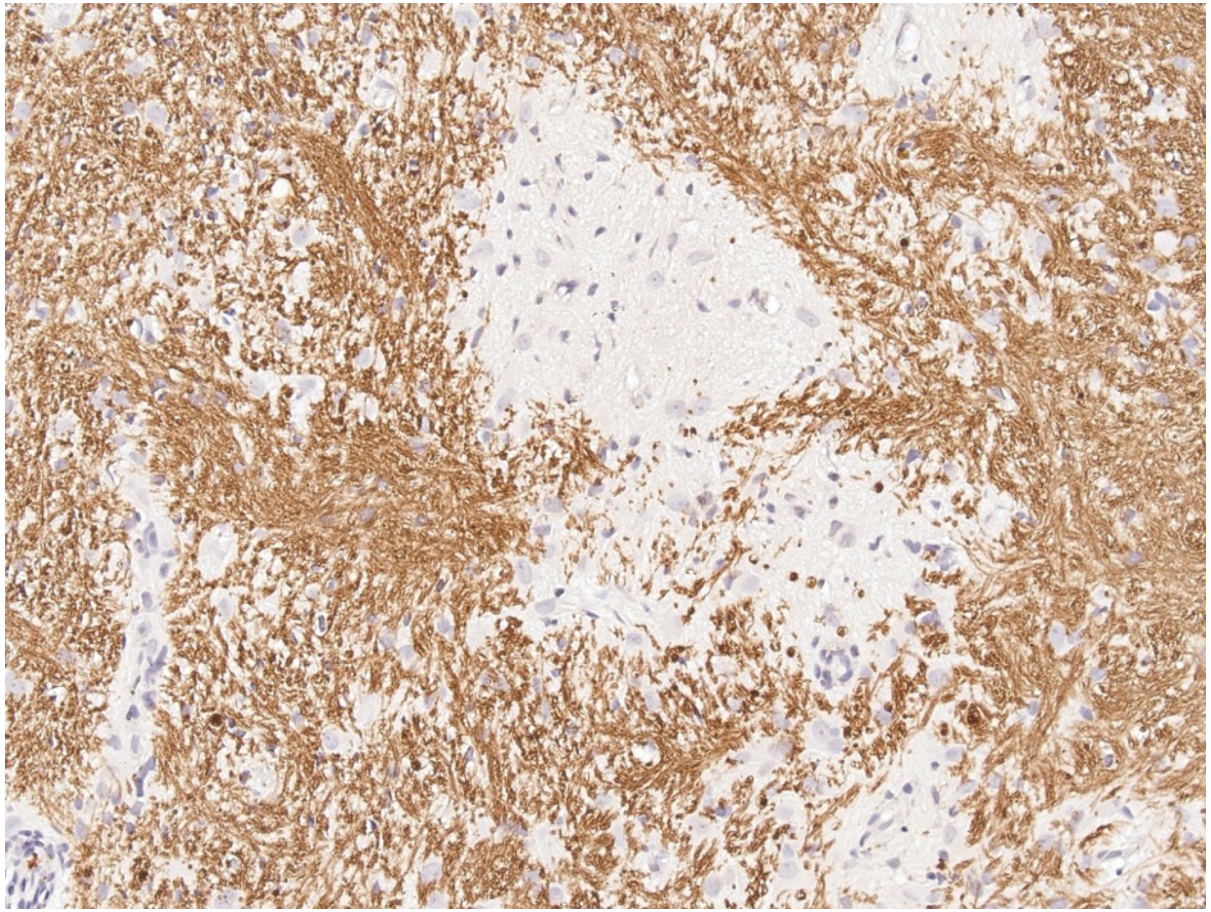

Remyelinated

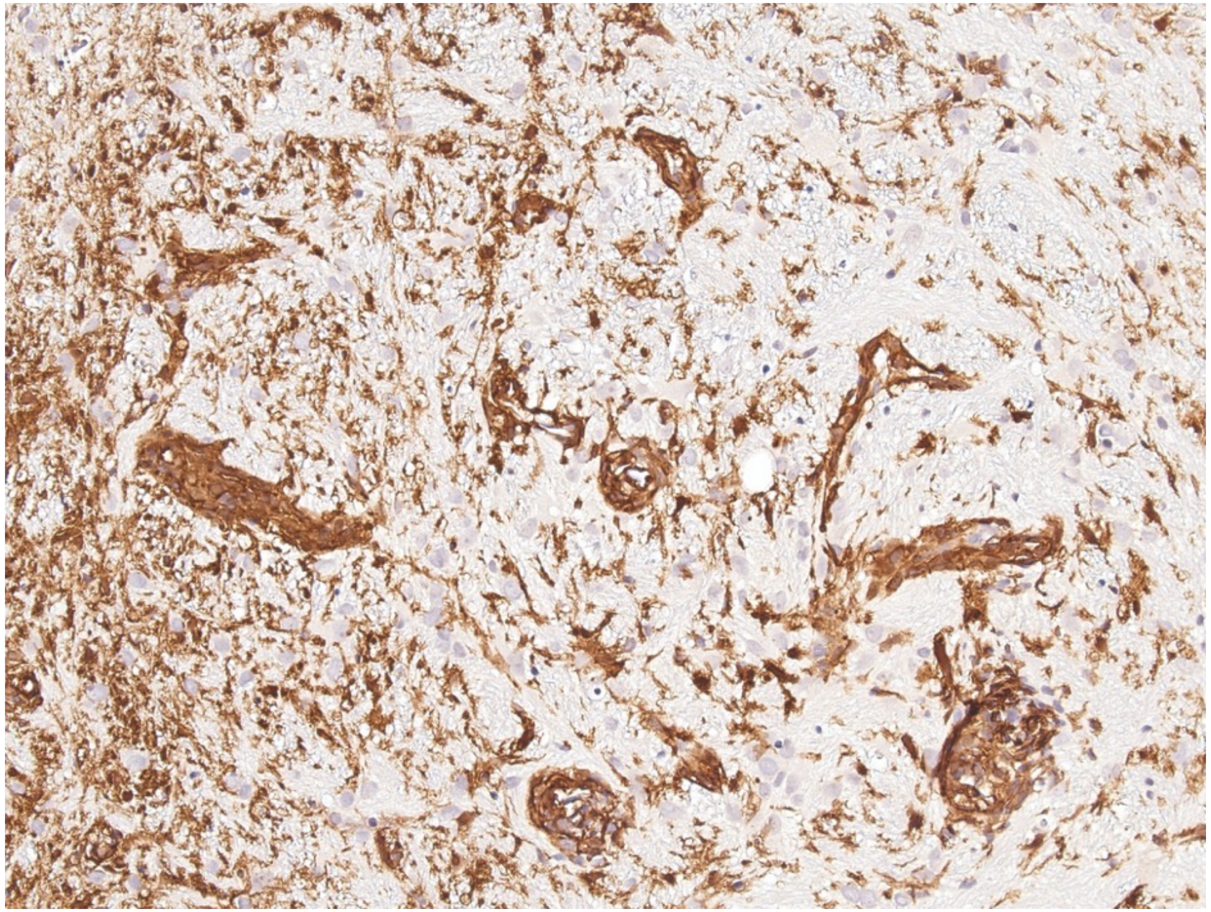

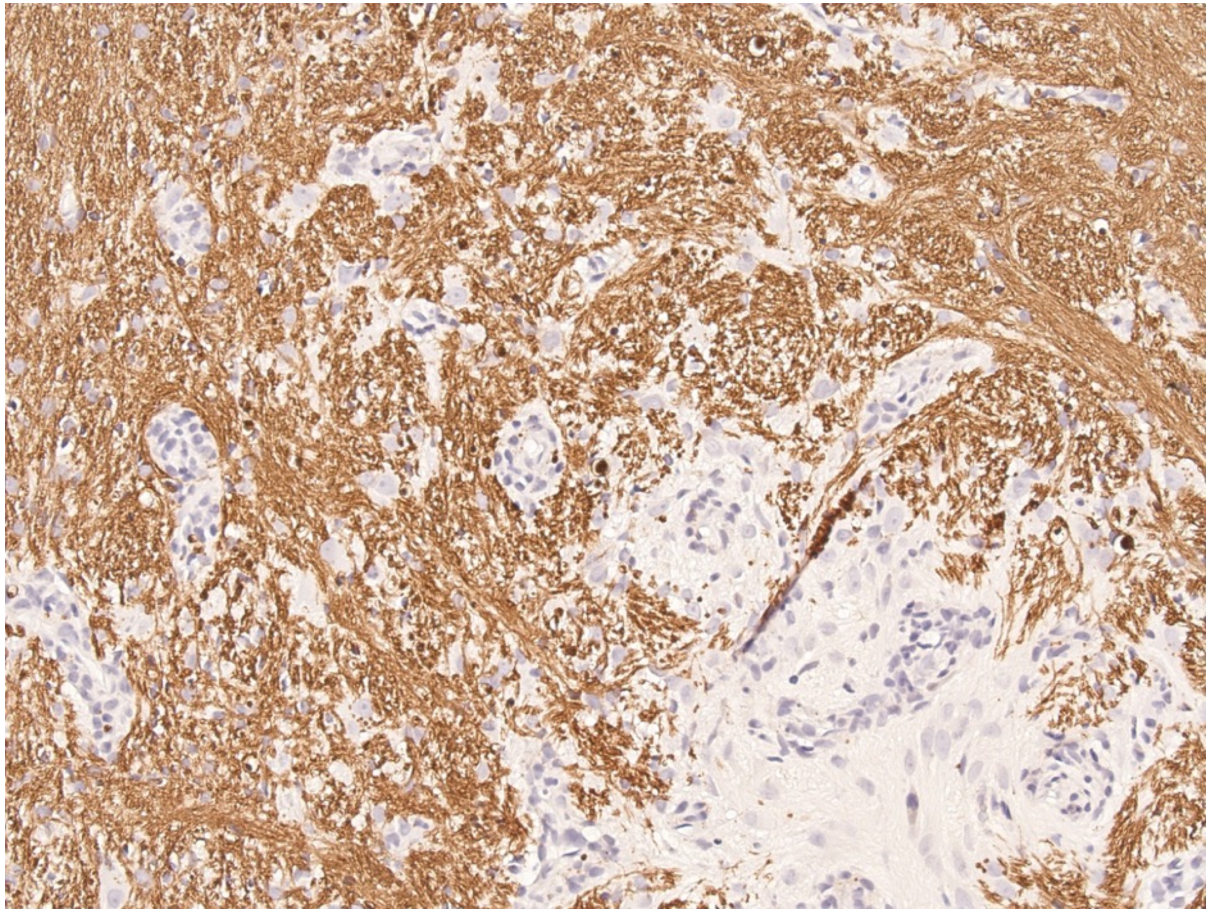

Remyelinated

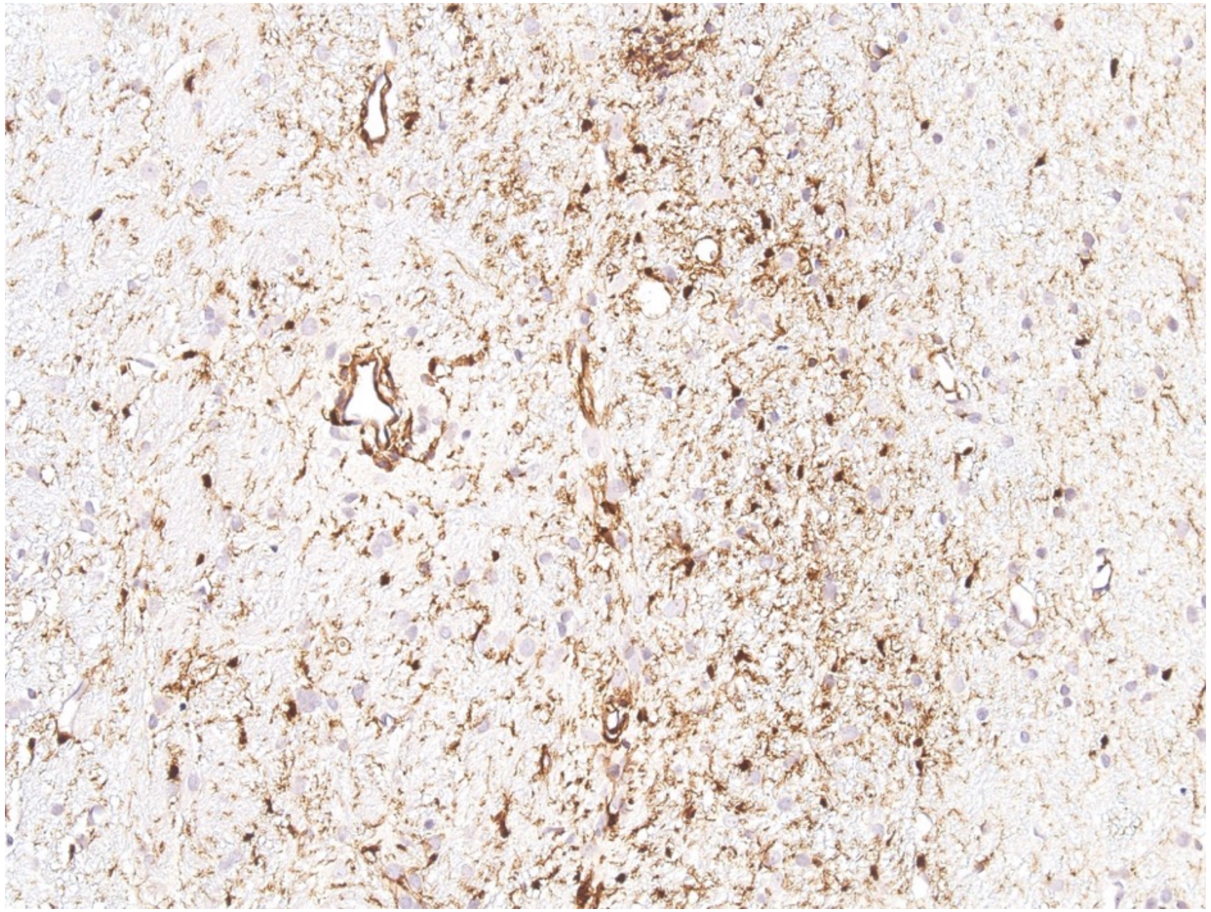

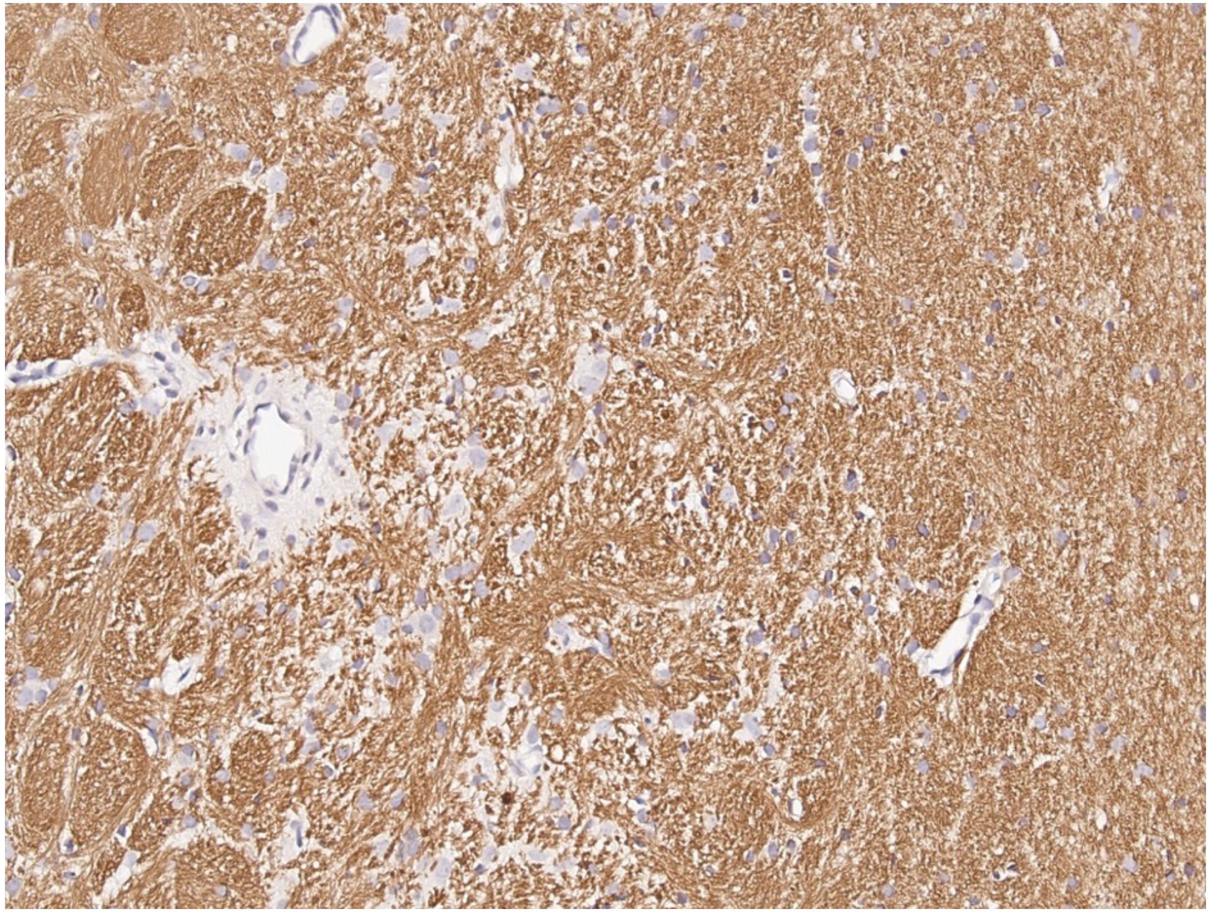

M#3

- 1 Chronic Demyelinated
- 5 Remyelinated

Chronic Demyelinated

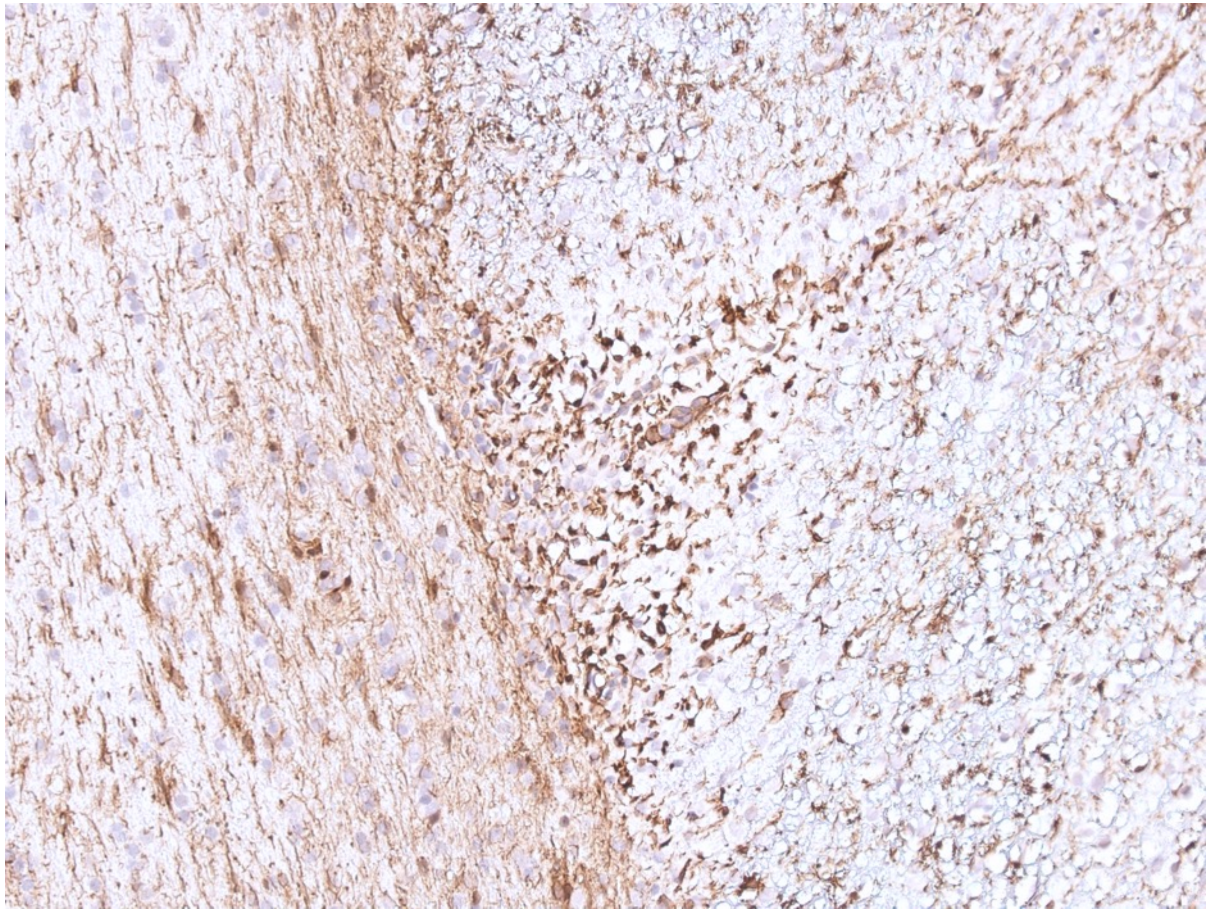

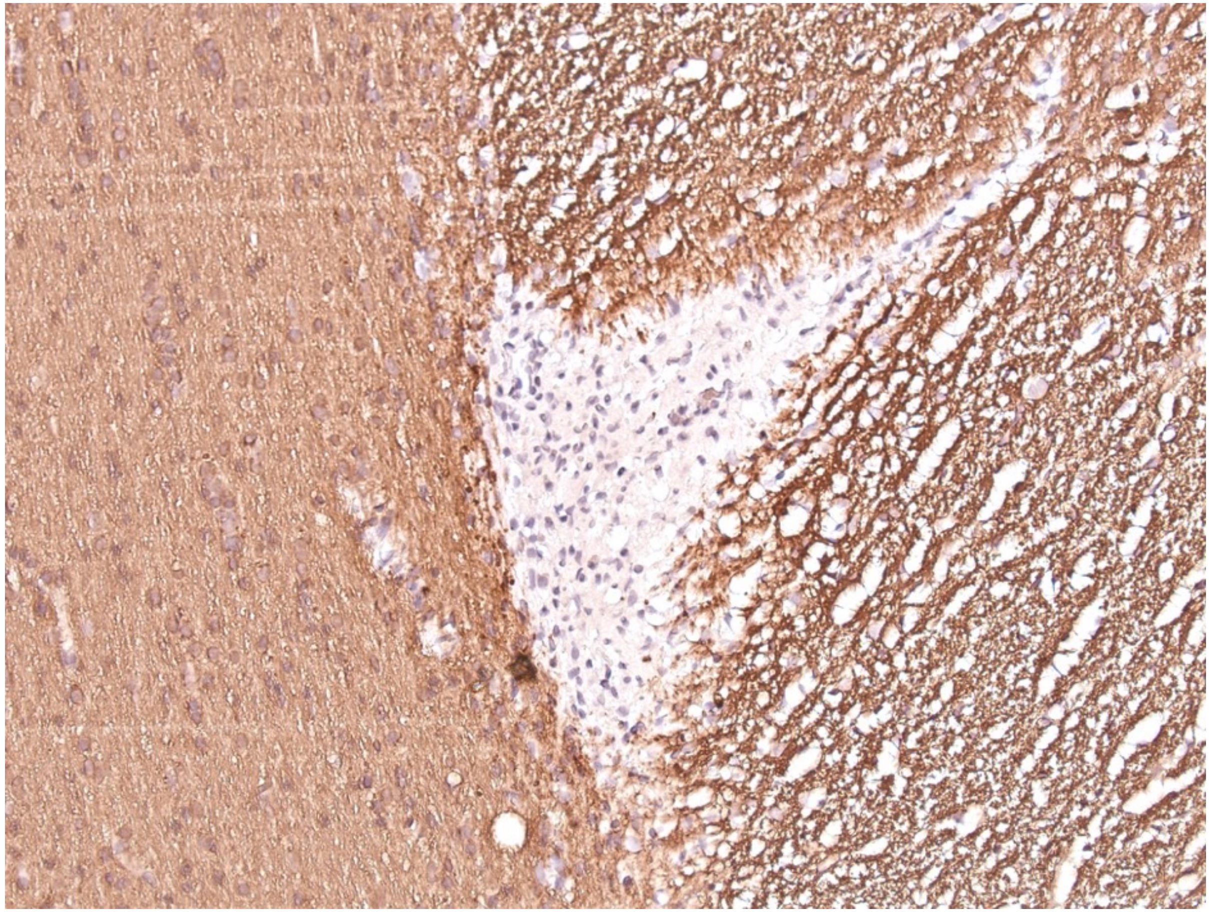

Remyelinated

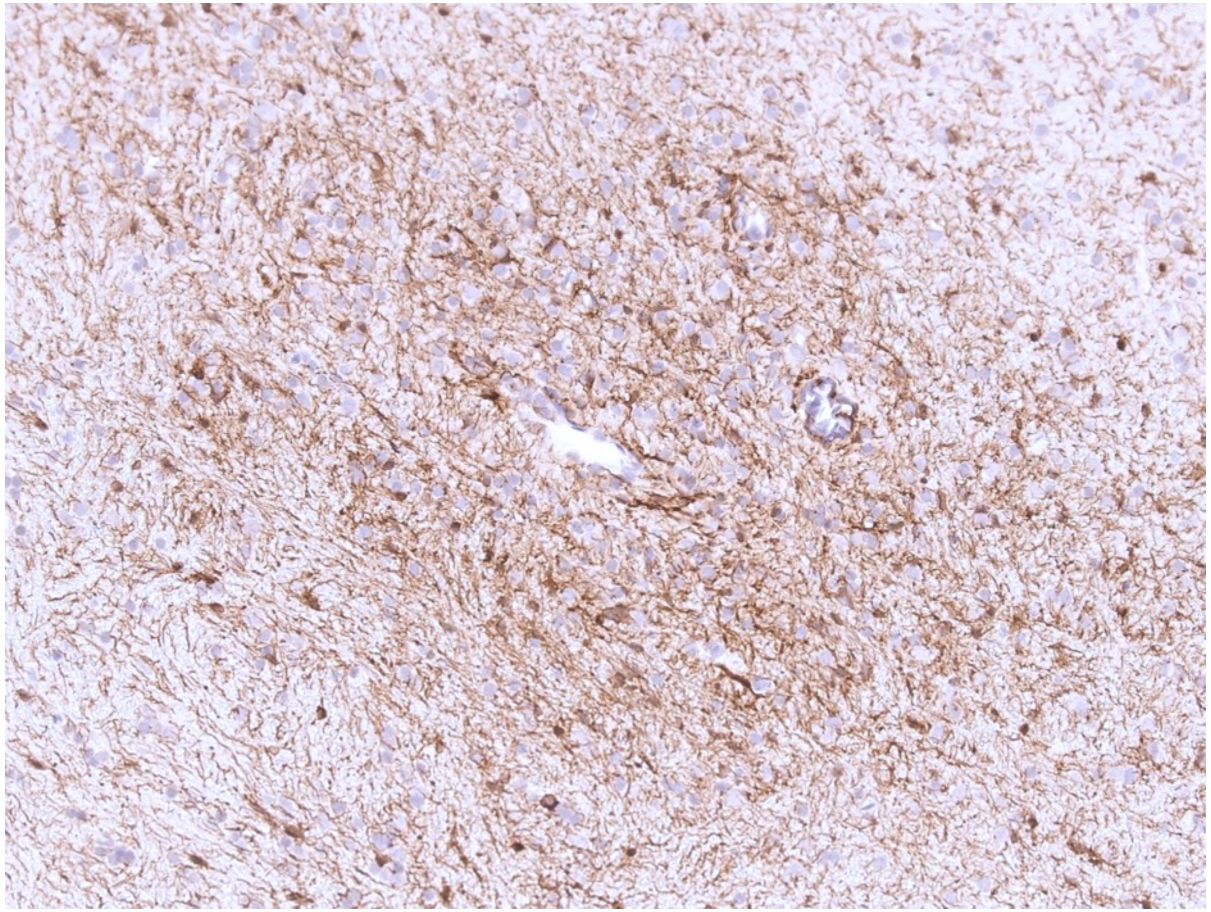

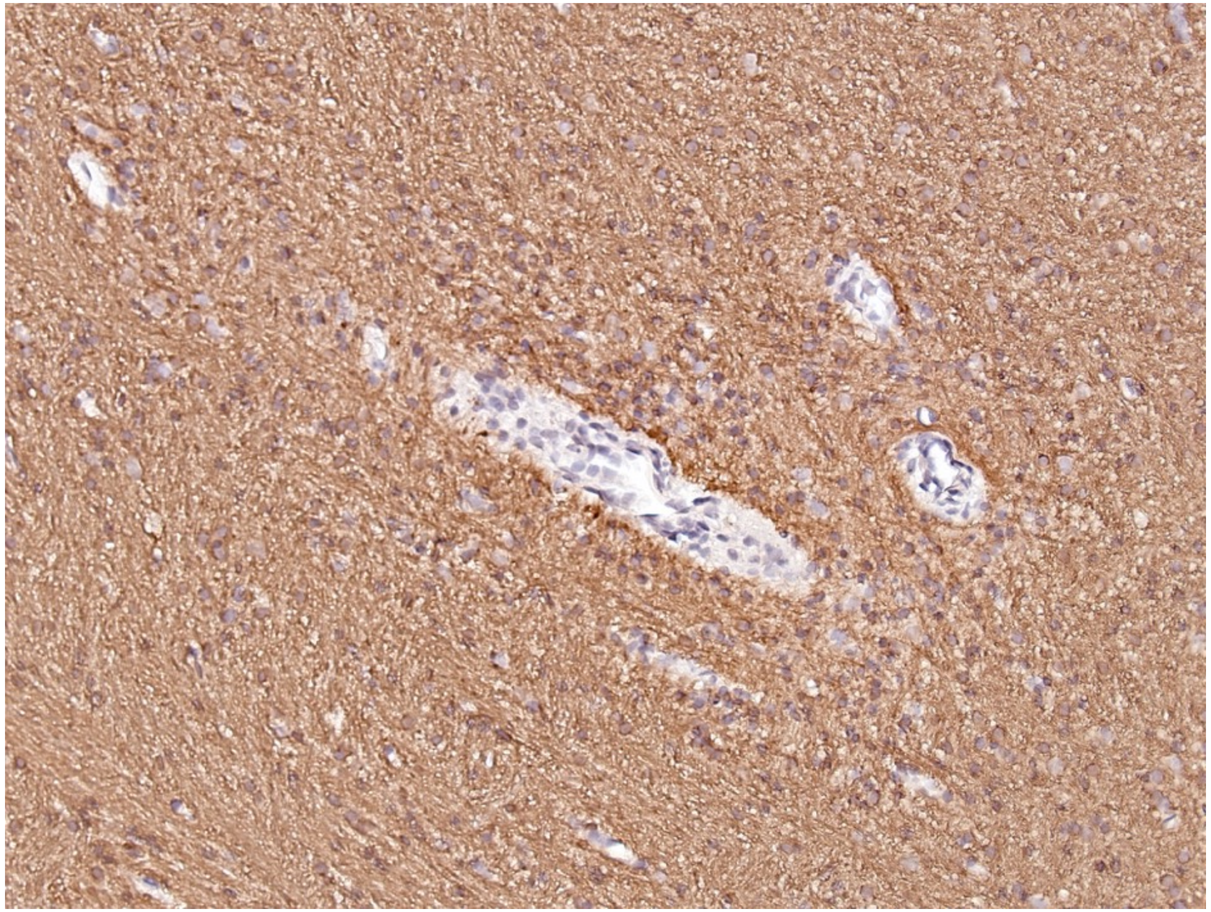

Remyelinated

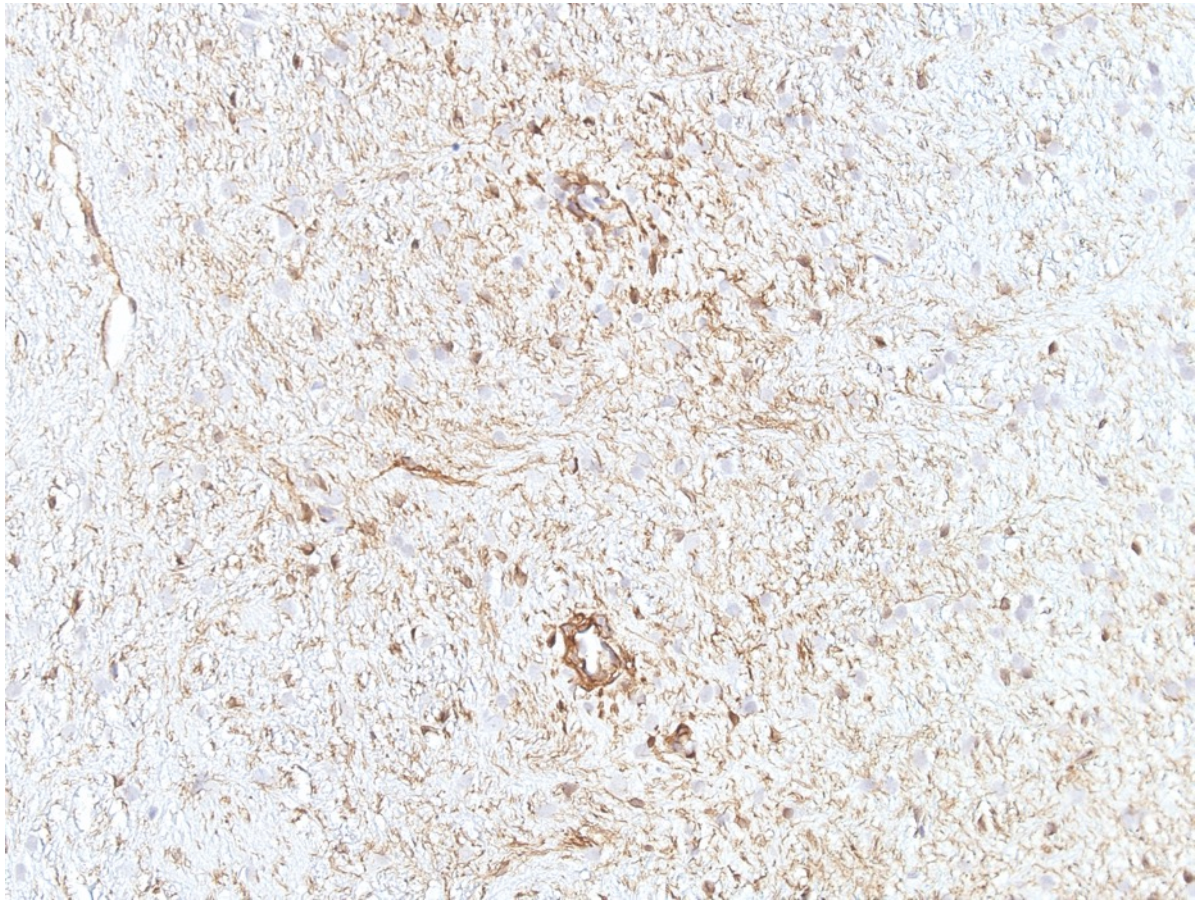

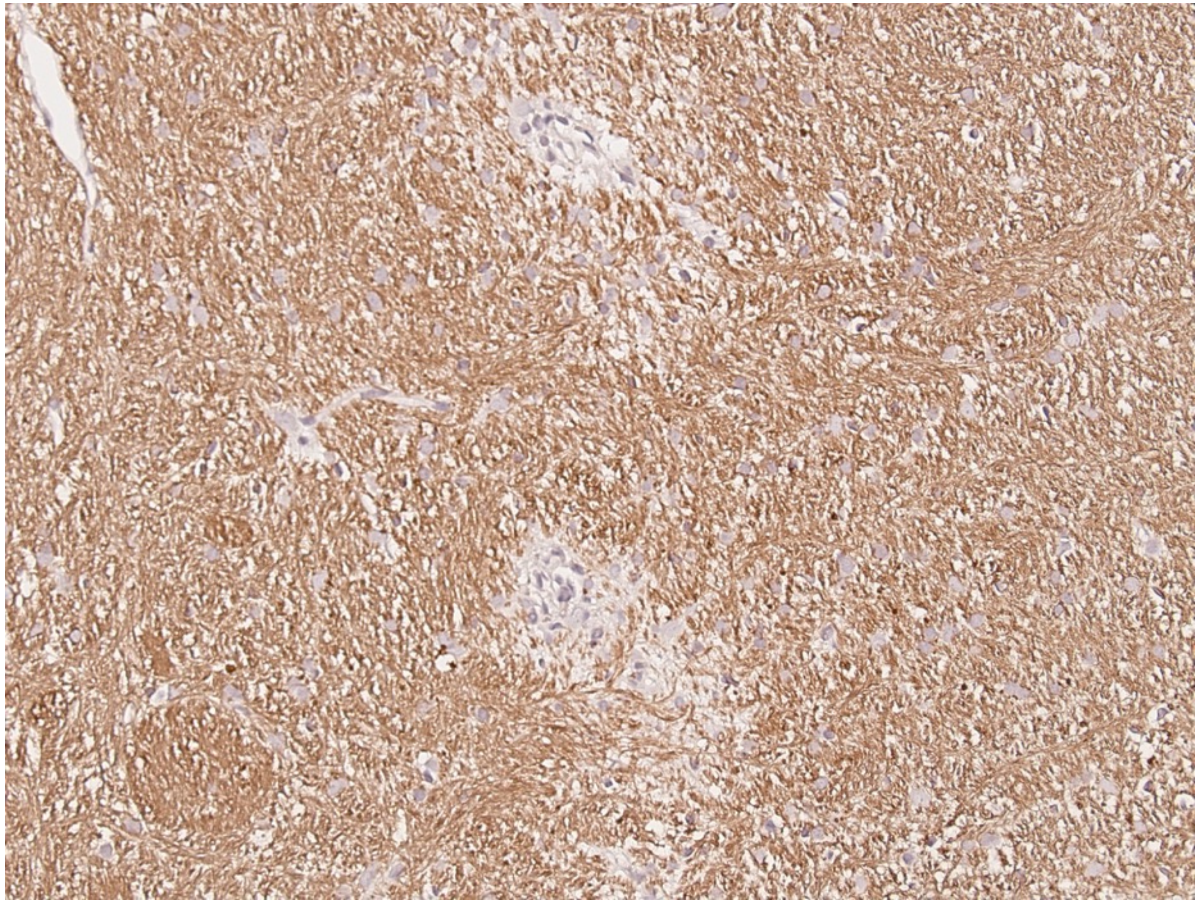

Remyelinated

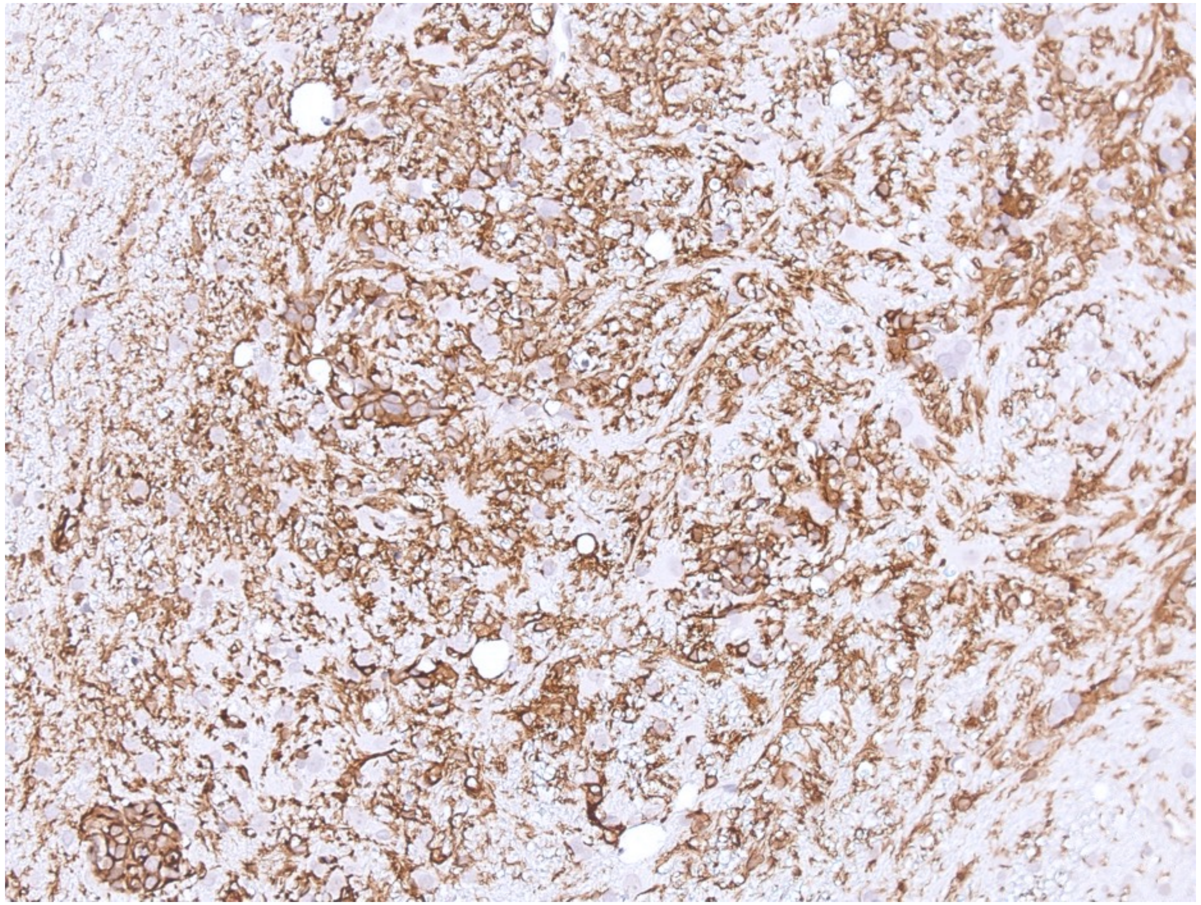

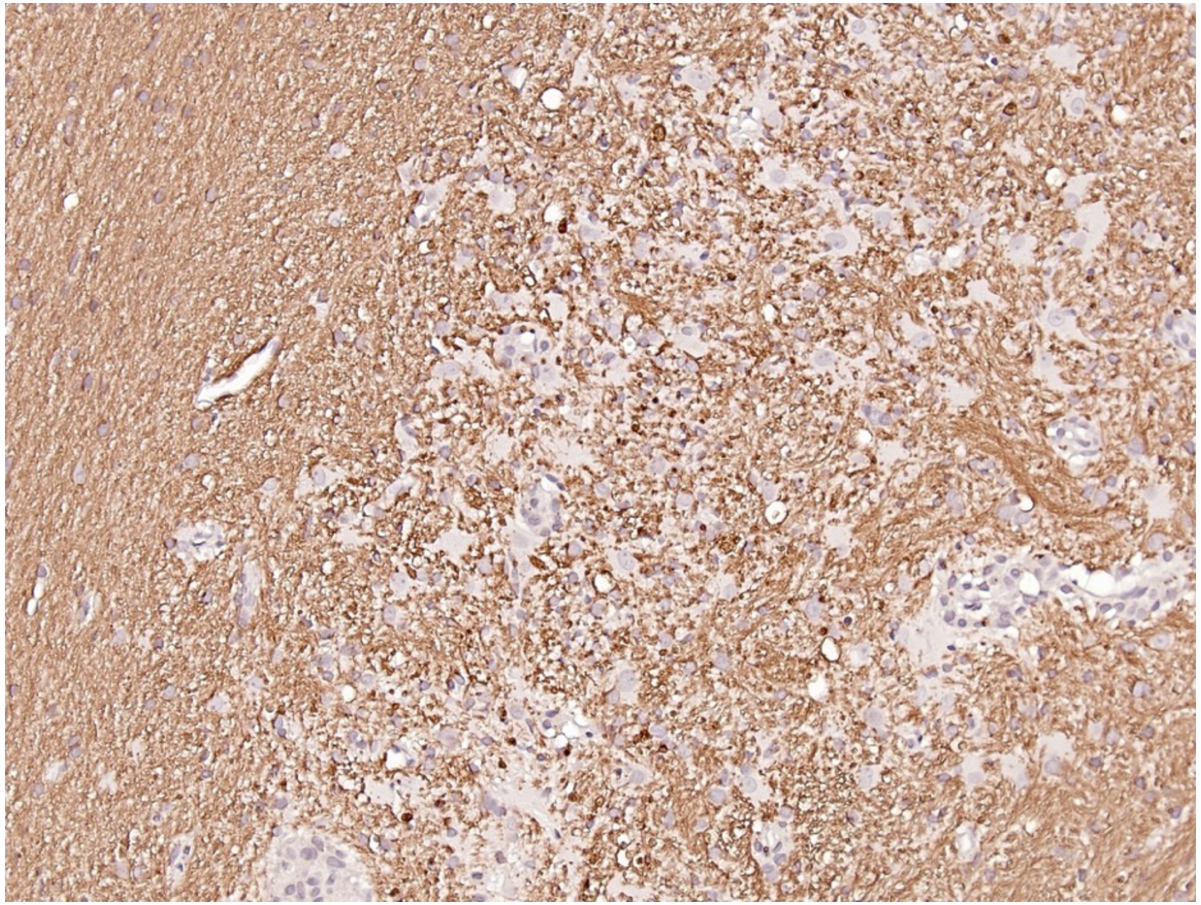

Remyelinated

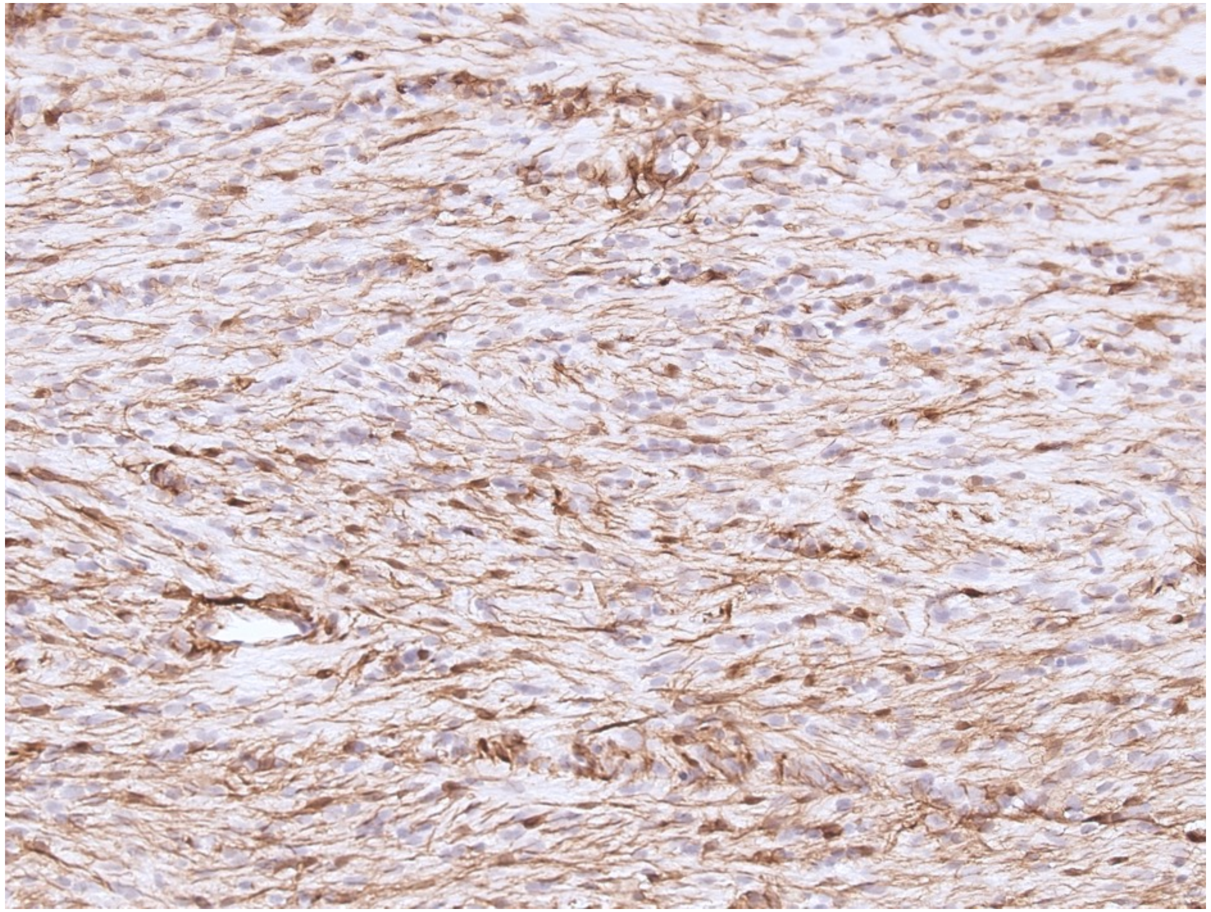

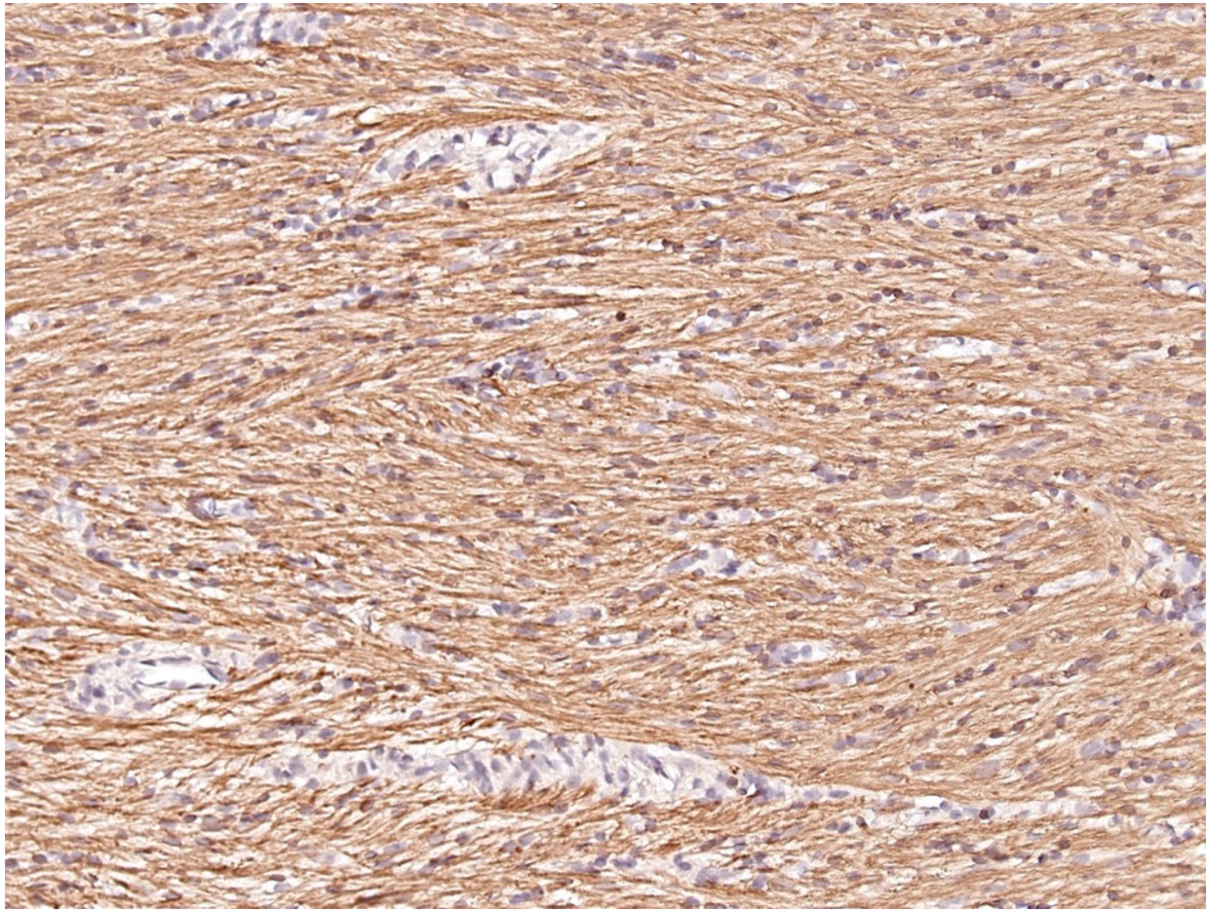

Remyelinated

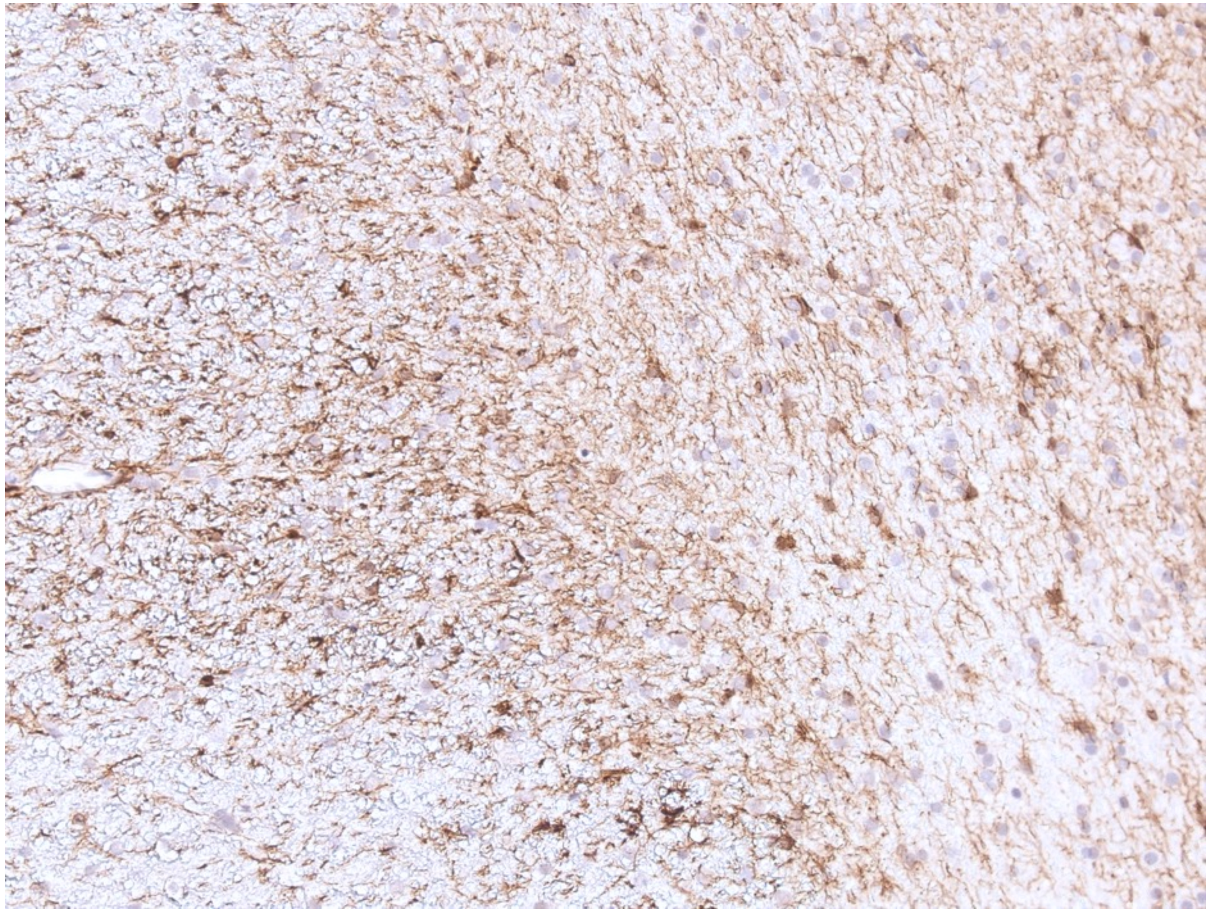

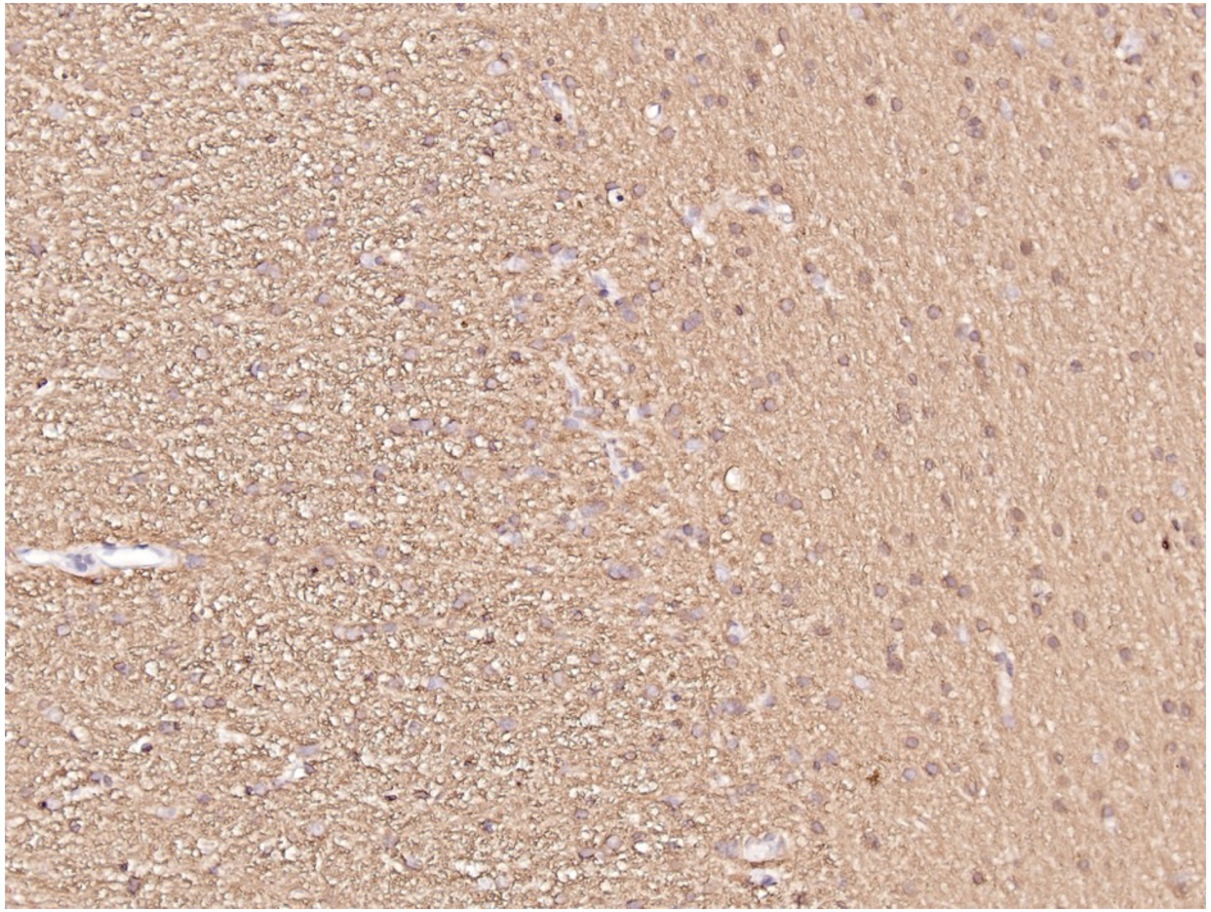

M#4

- 1 Chronic Demyelinated
- 2 Remyelinated

Chronic Demyelinated

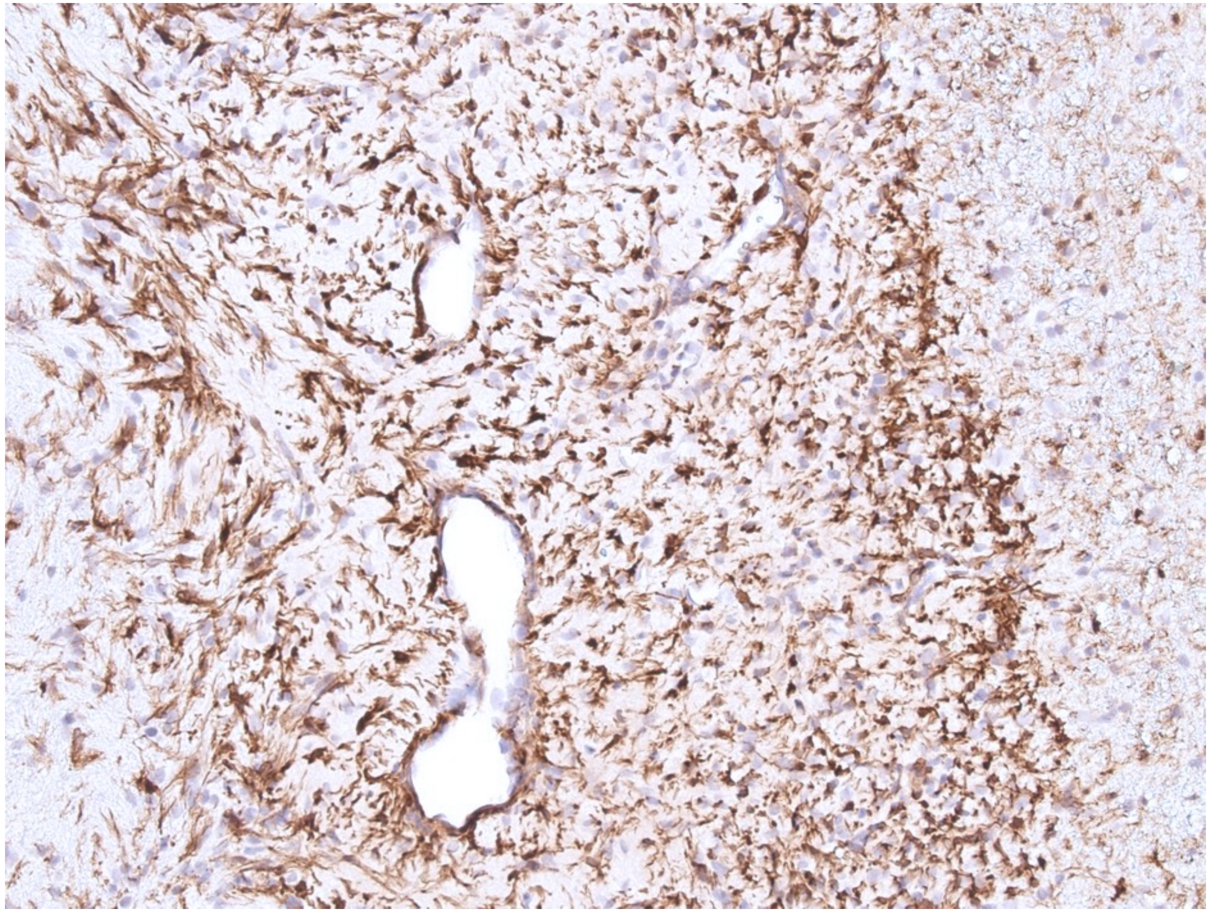

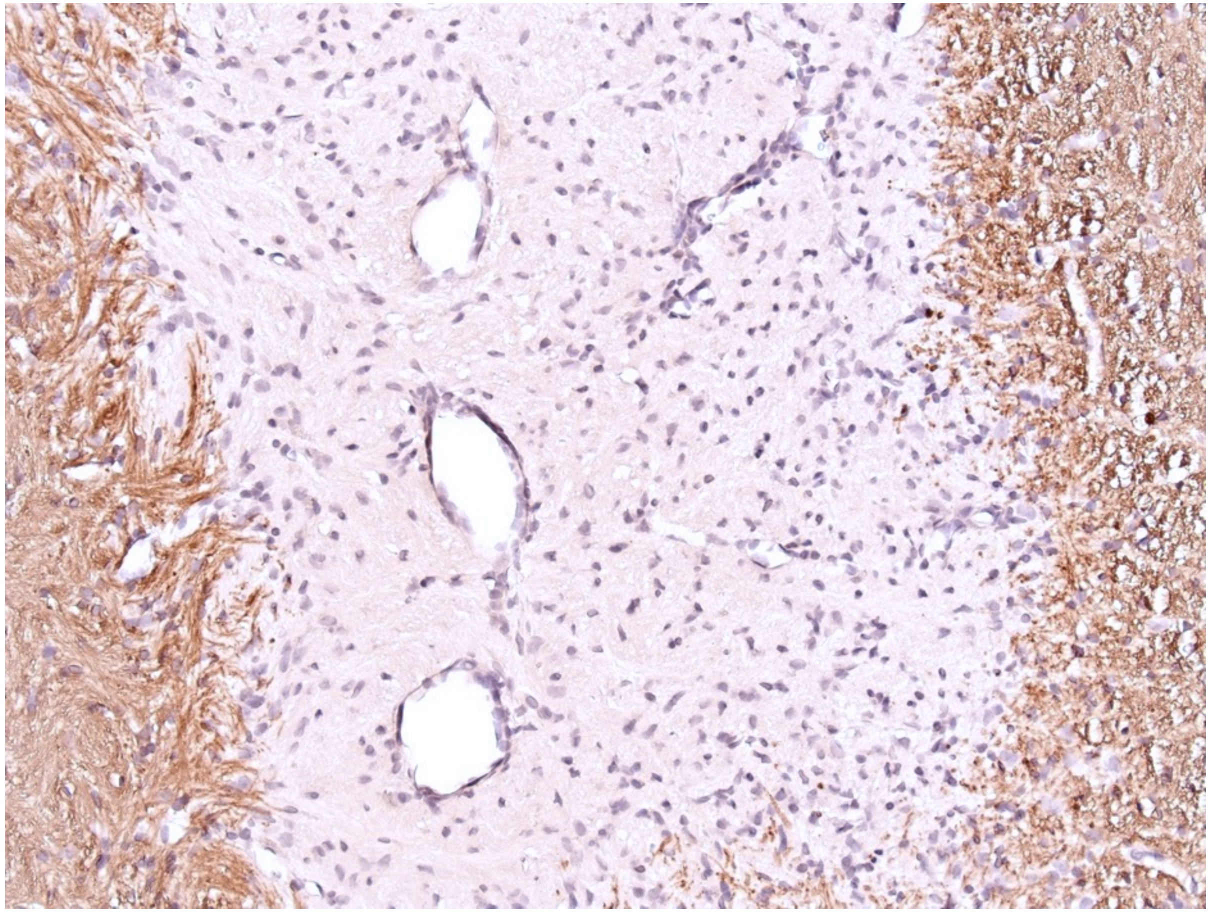

Remyelinated

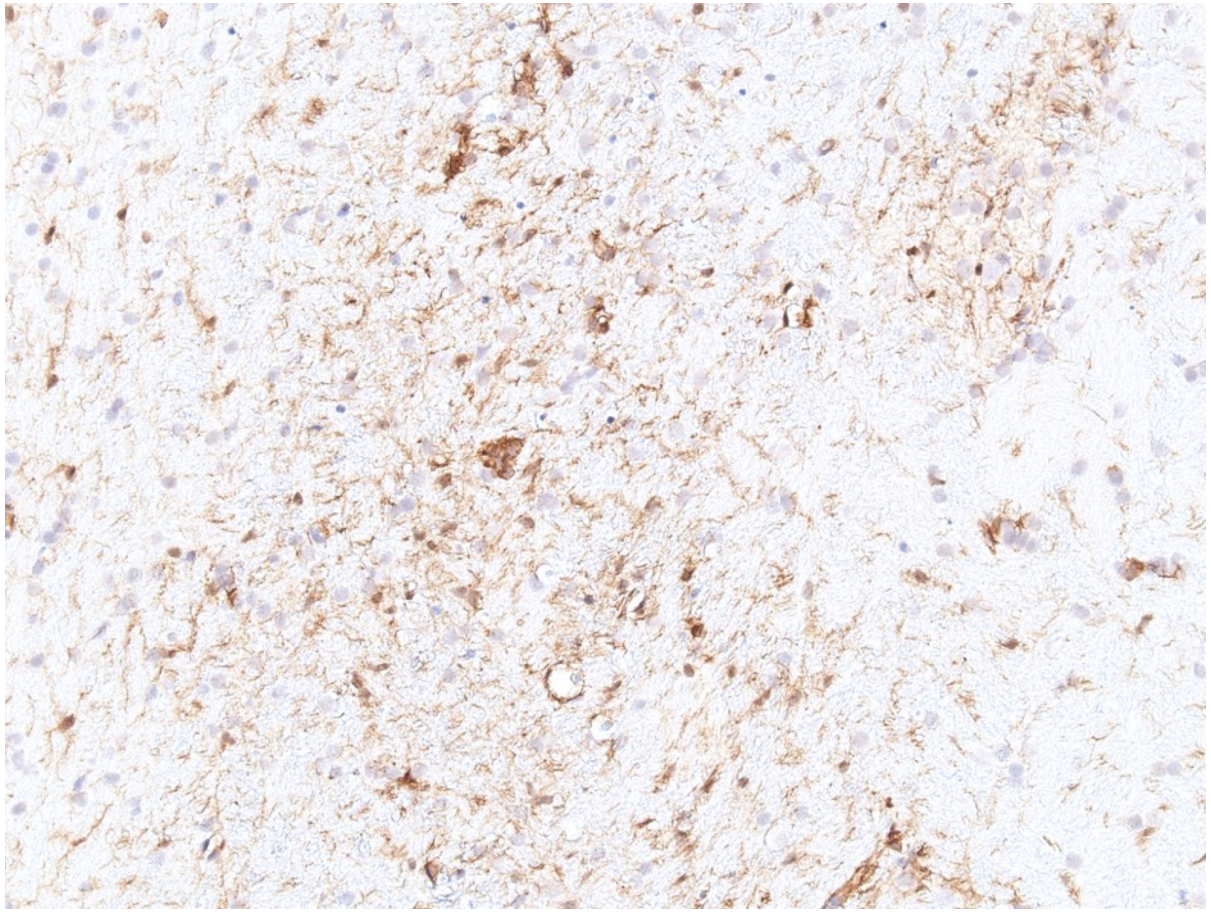

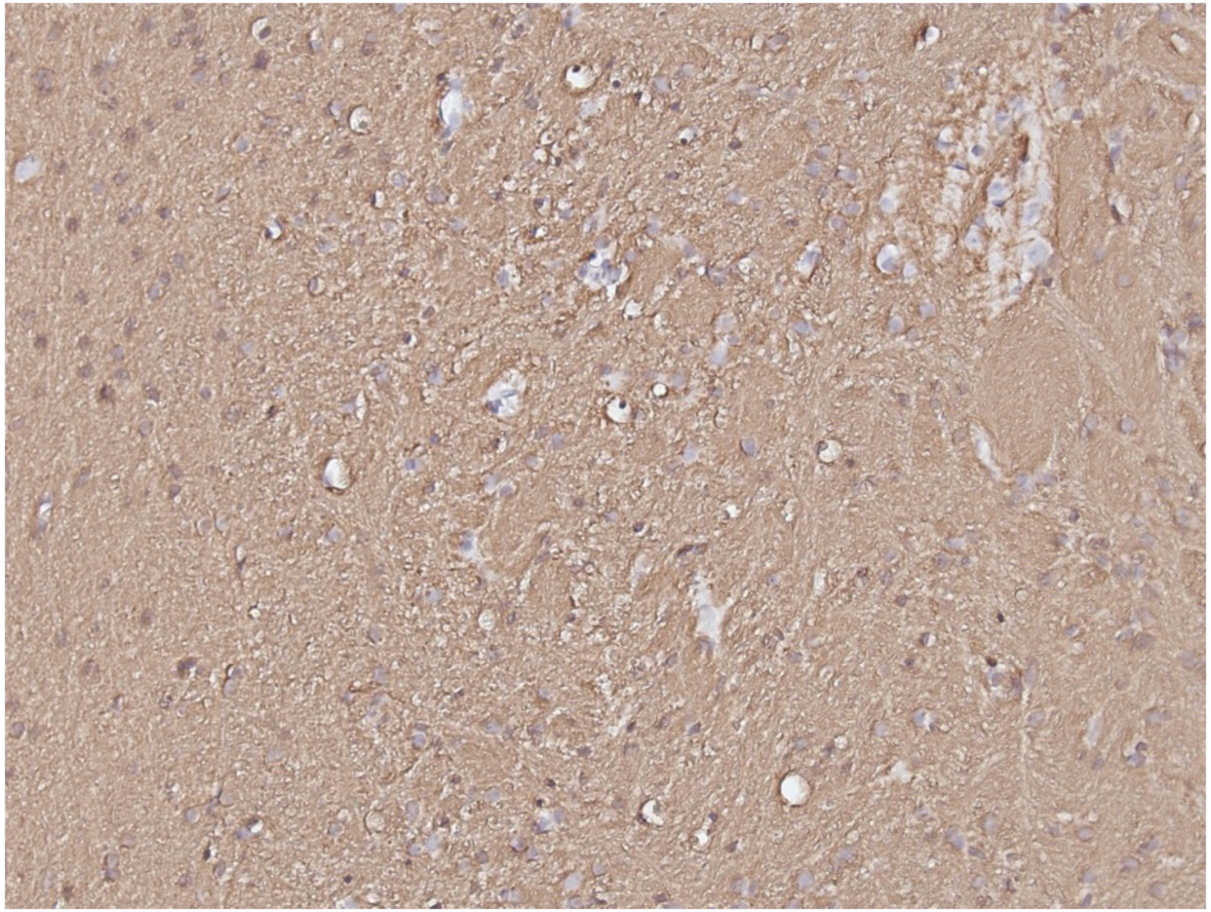

Remyelinated

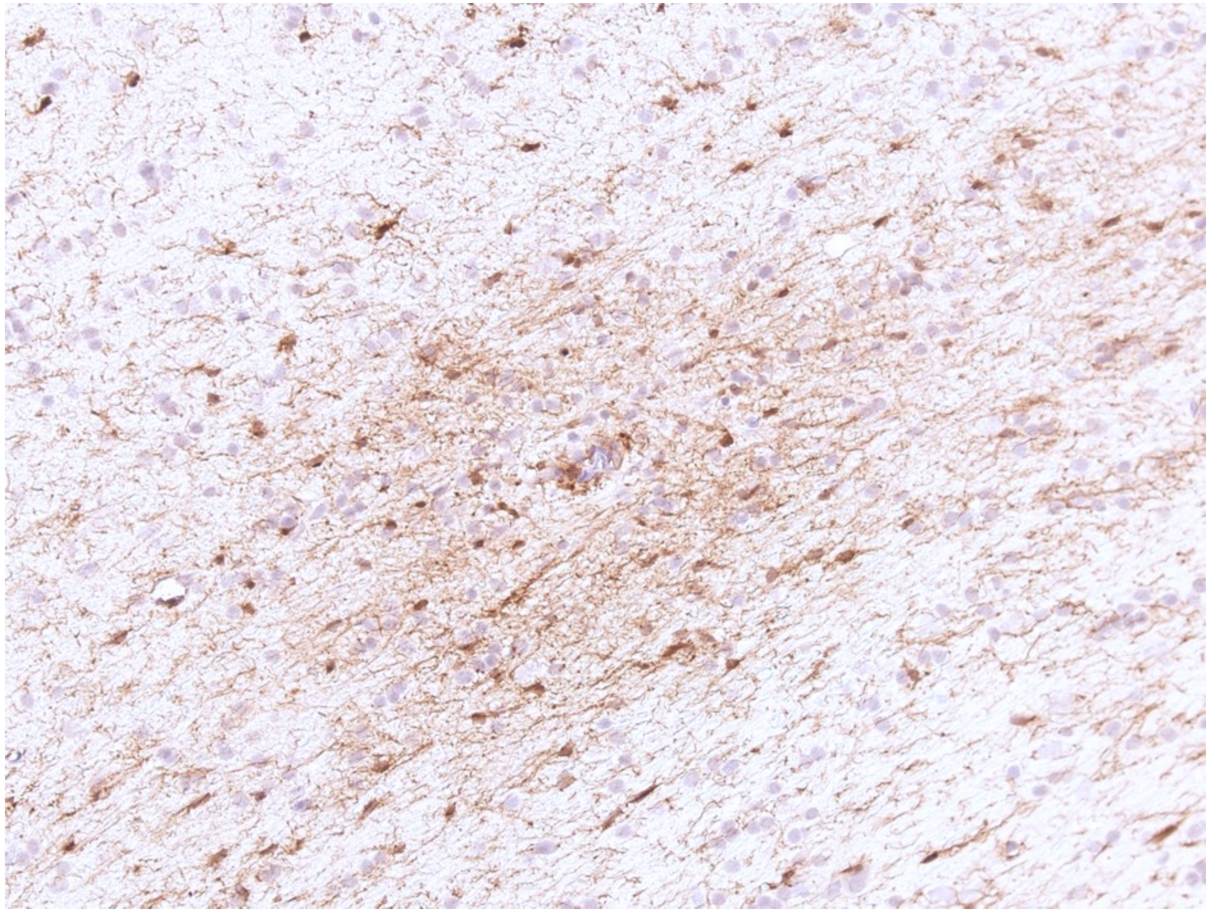

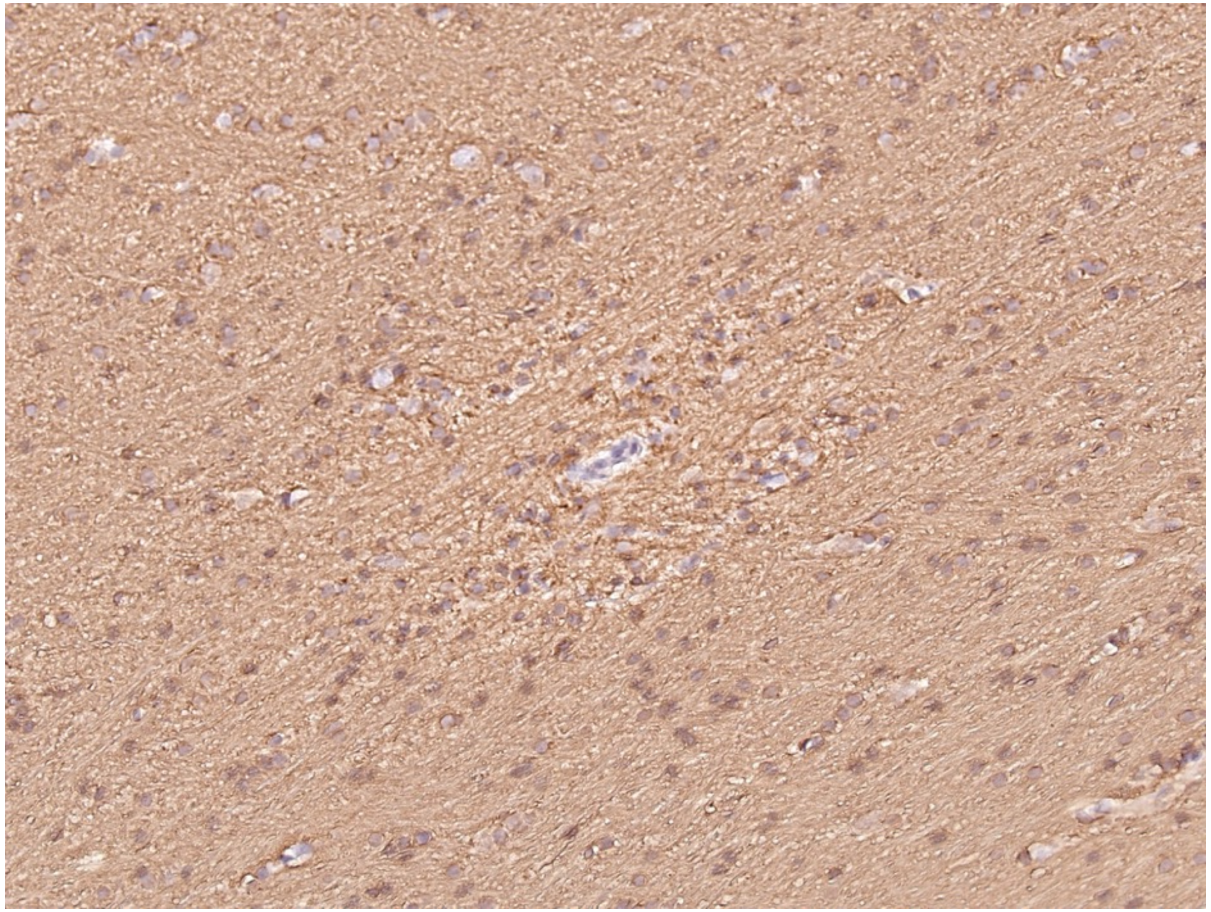

M#5

- 4 Acute Demyelinating
- 4 Chronic Demyelinated
- 1 Remyelinated

Acute Demyelinating

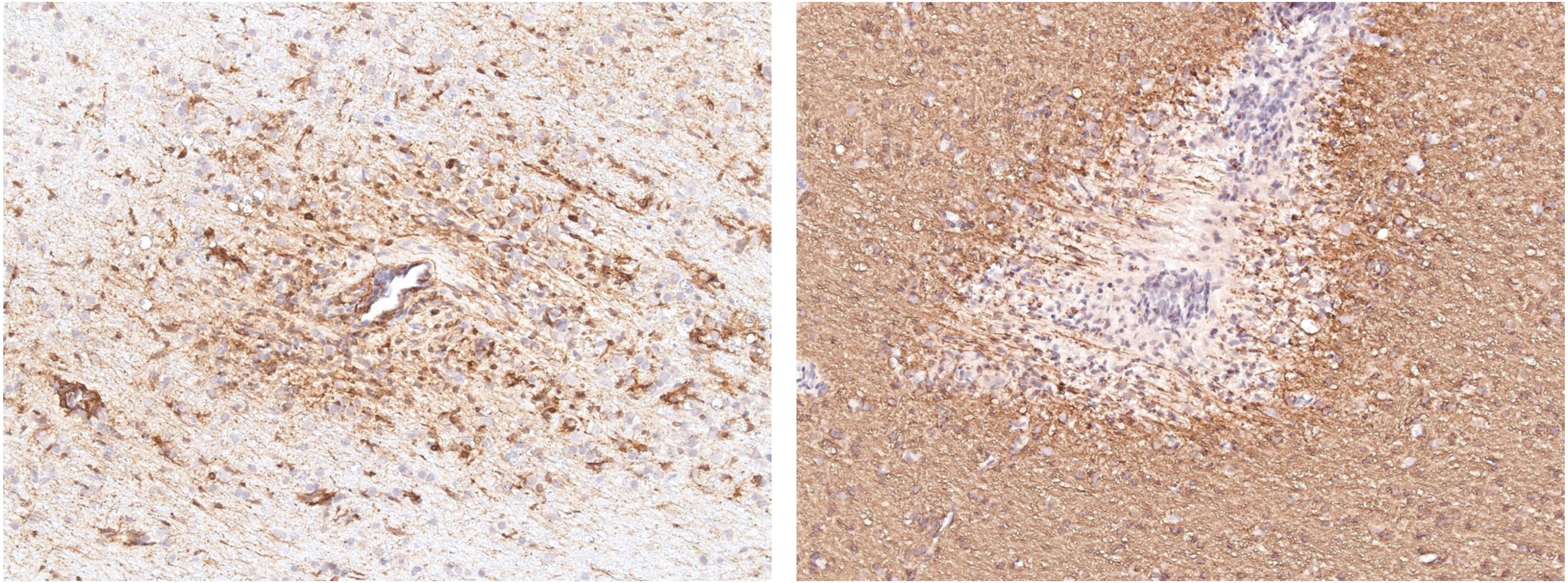

Acute Demyelinating

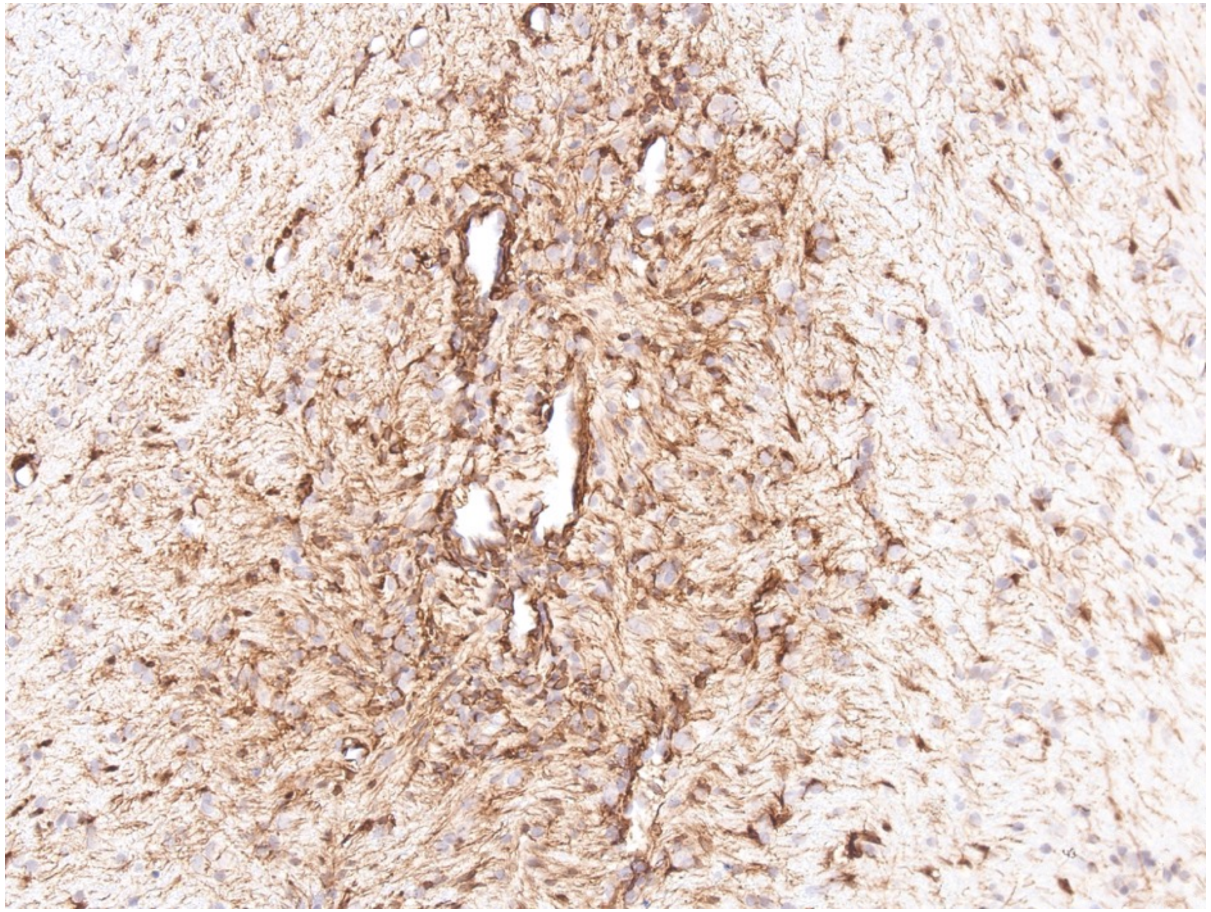

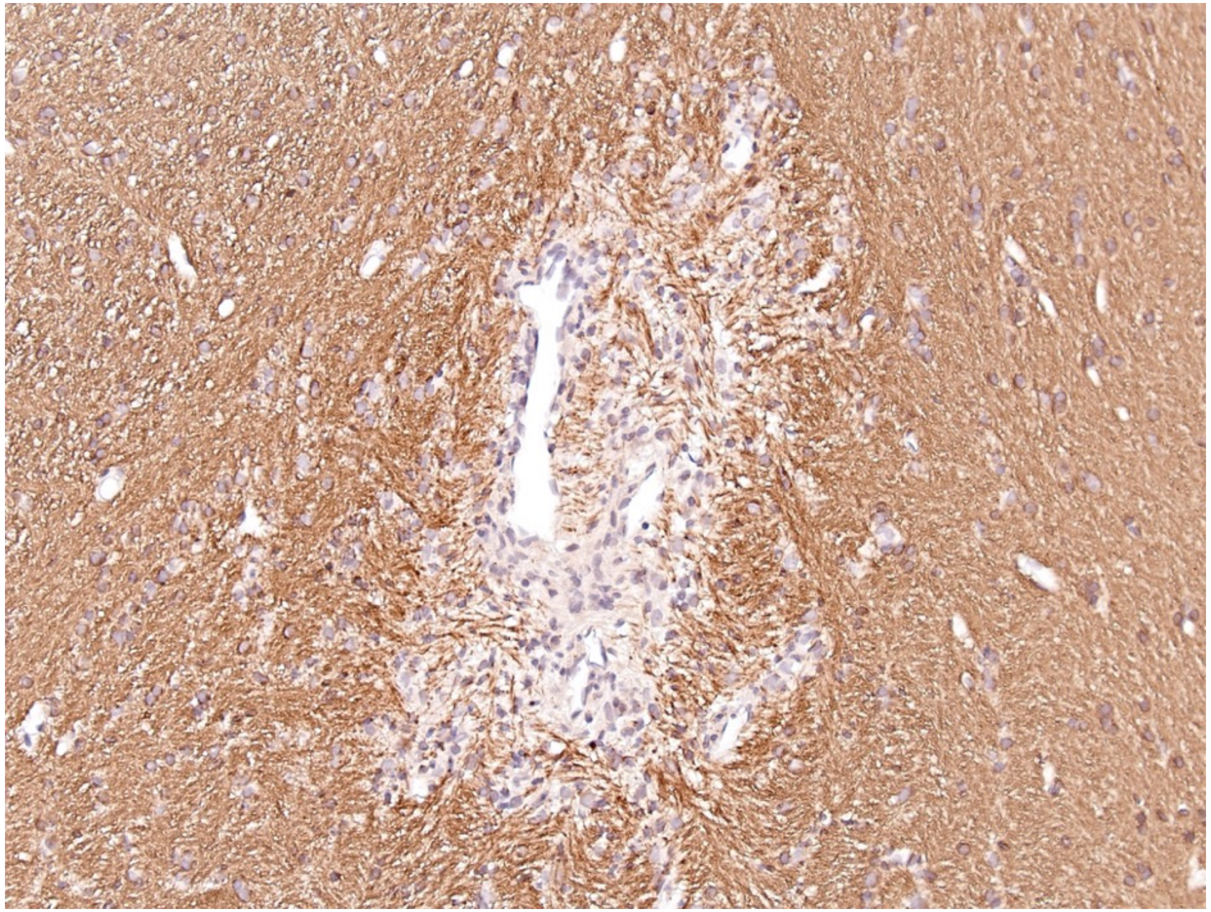

Acute Demyelinating

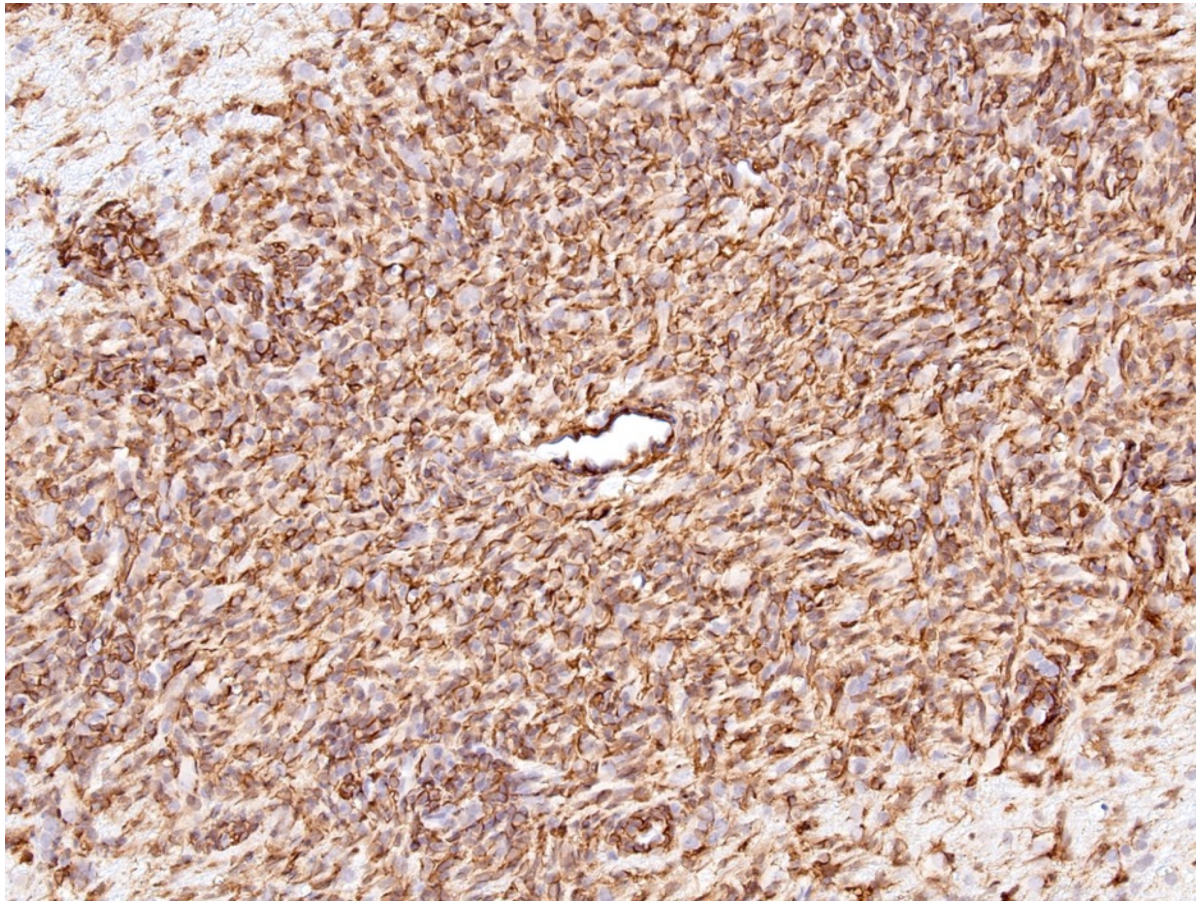

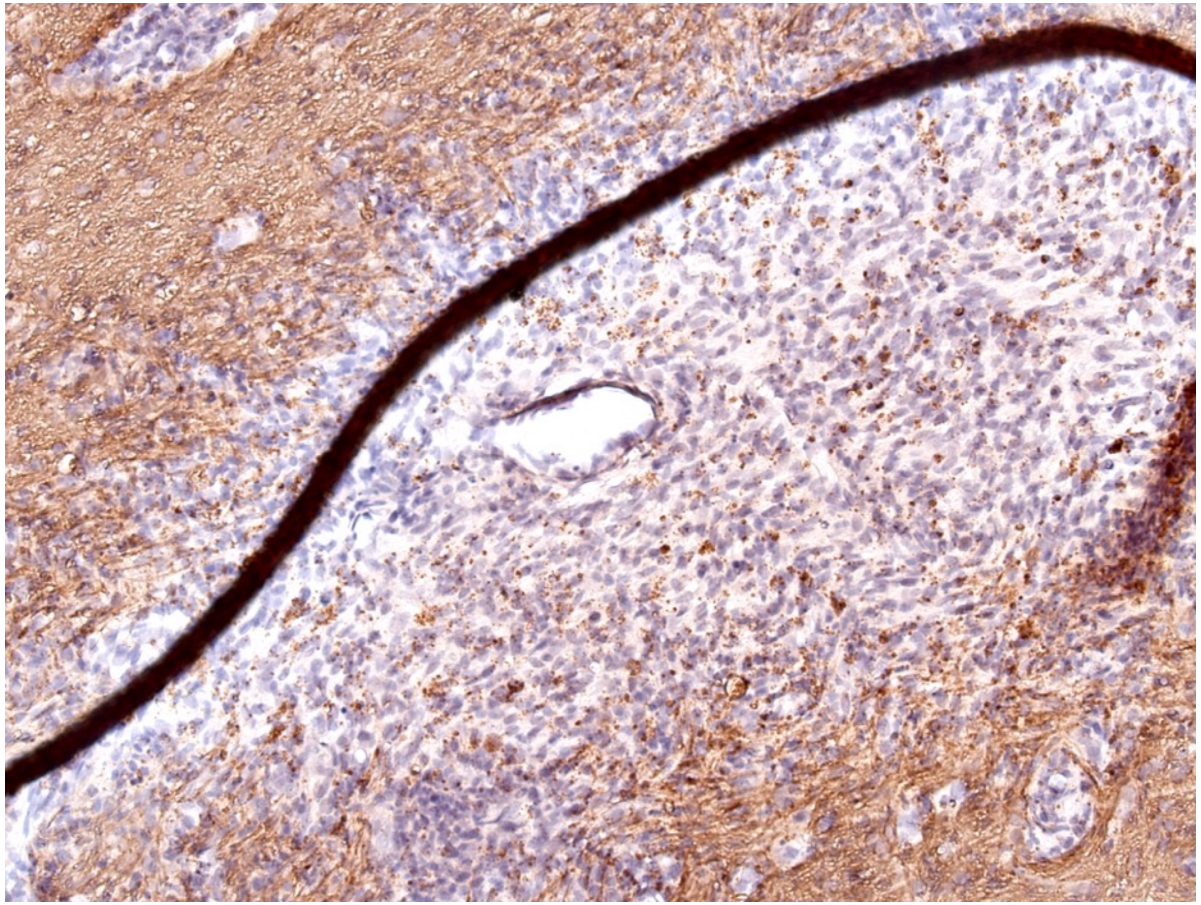

Acute Demyelinating

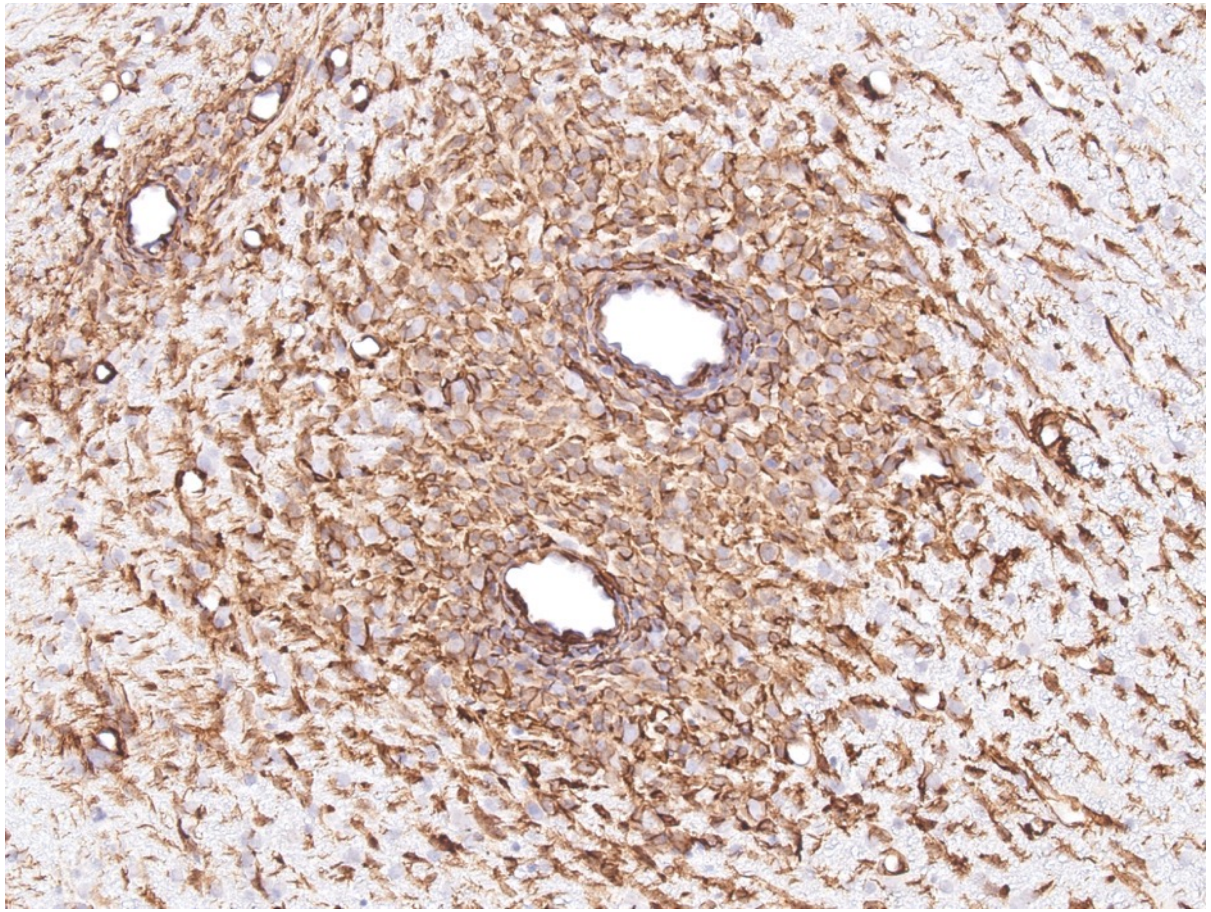

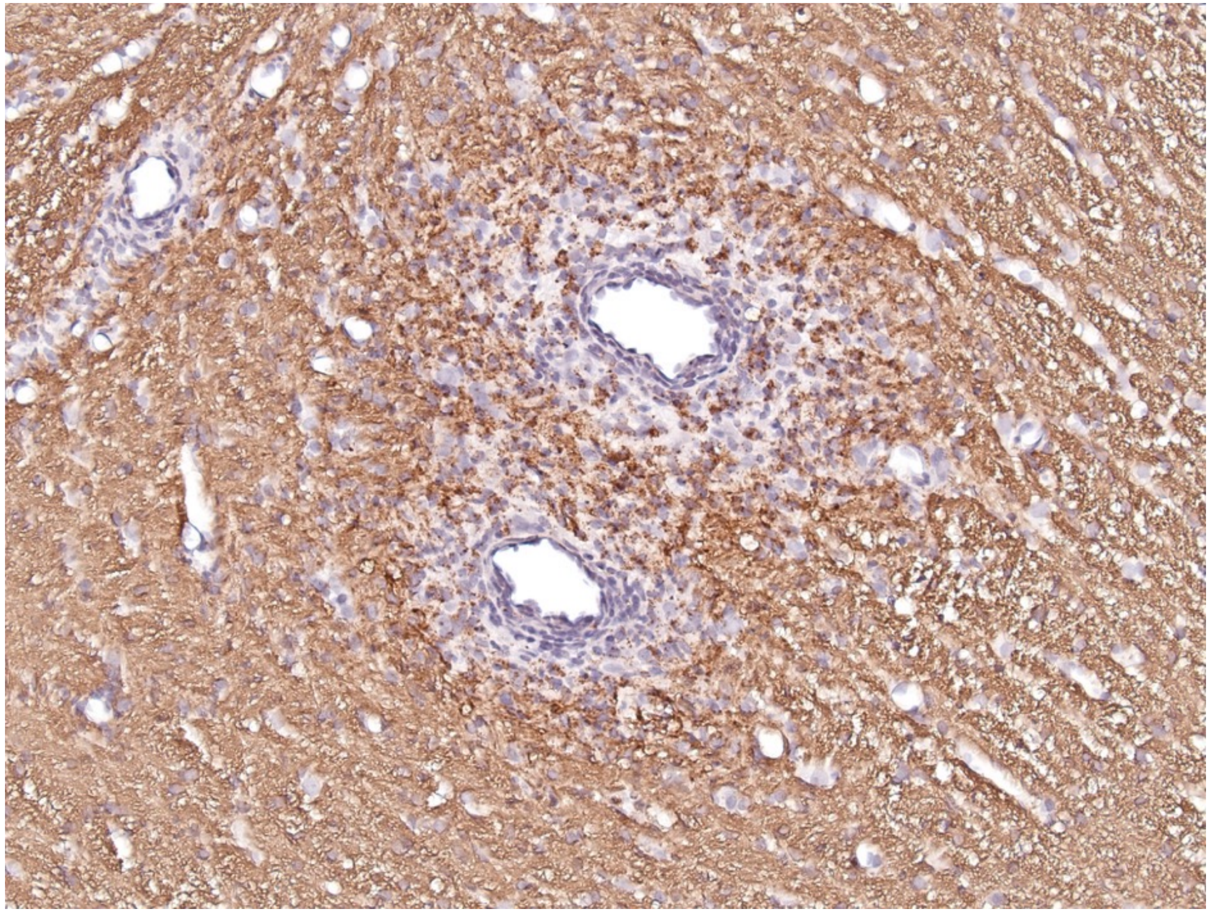

Chronic Demyelinated

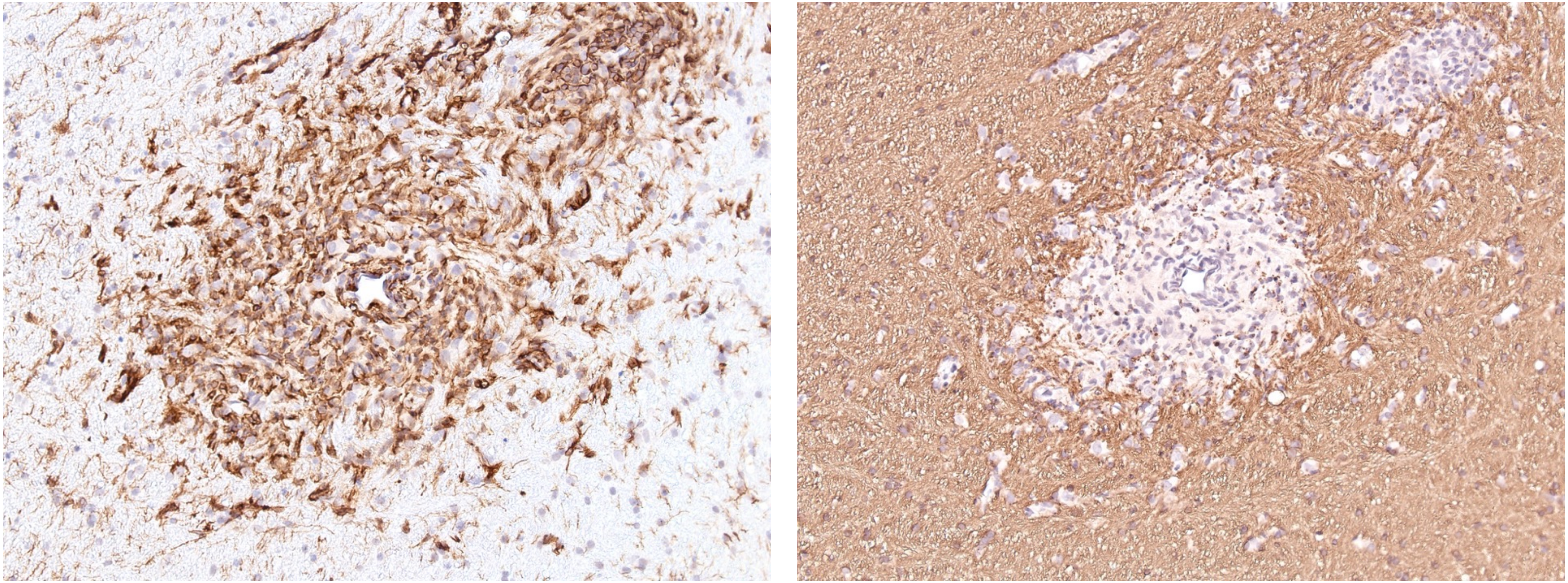

Chronic Demyelinated

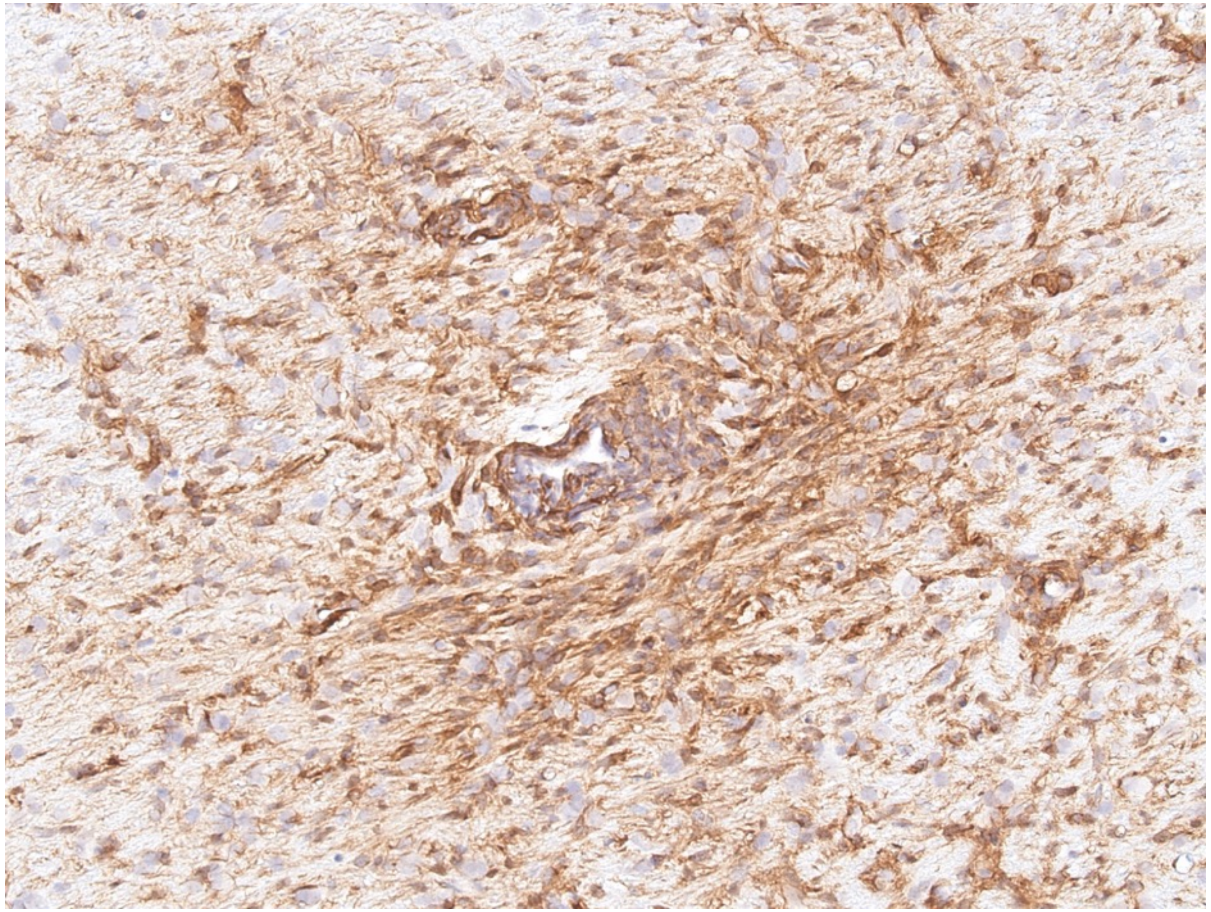

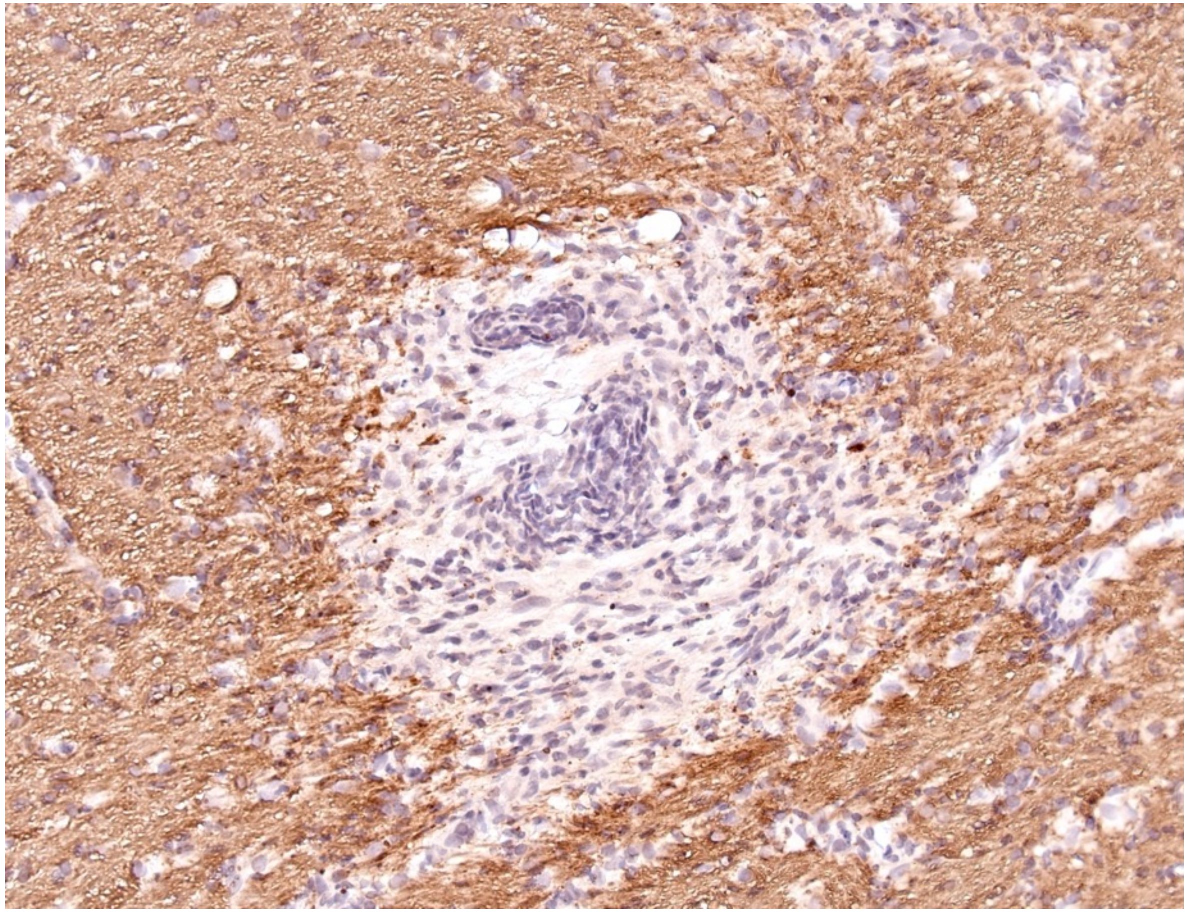

Chronic Demyelinated

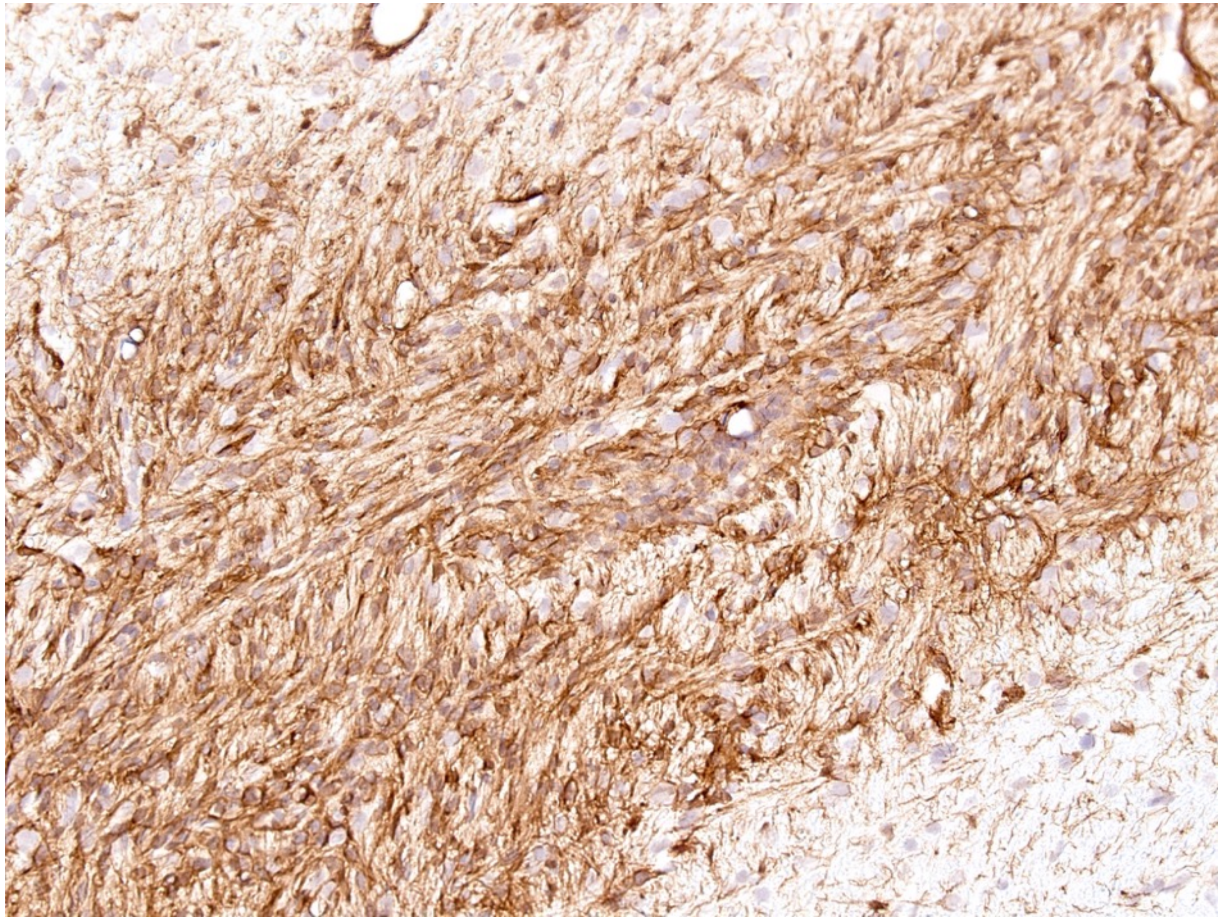

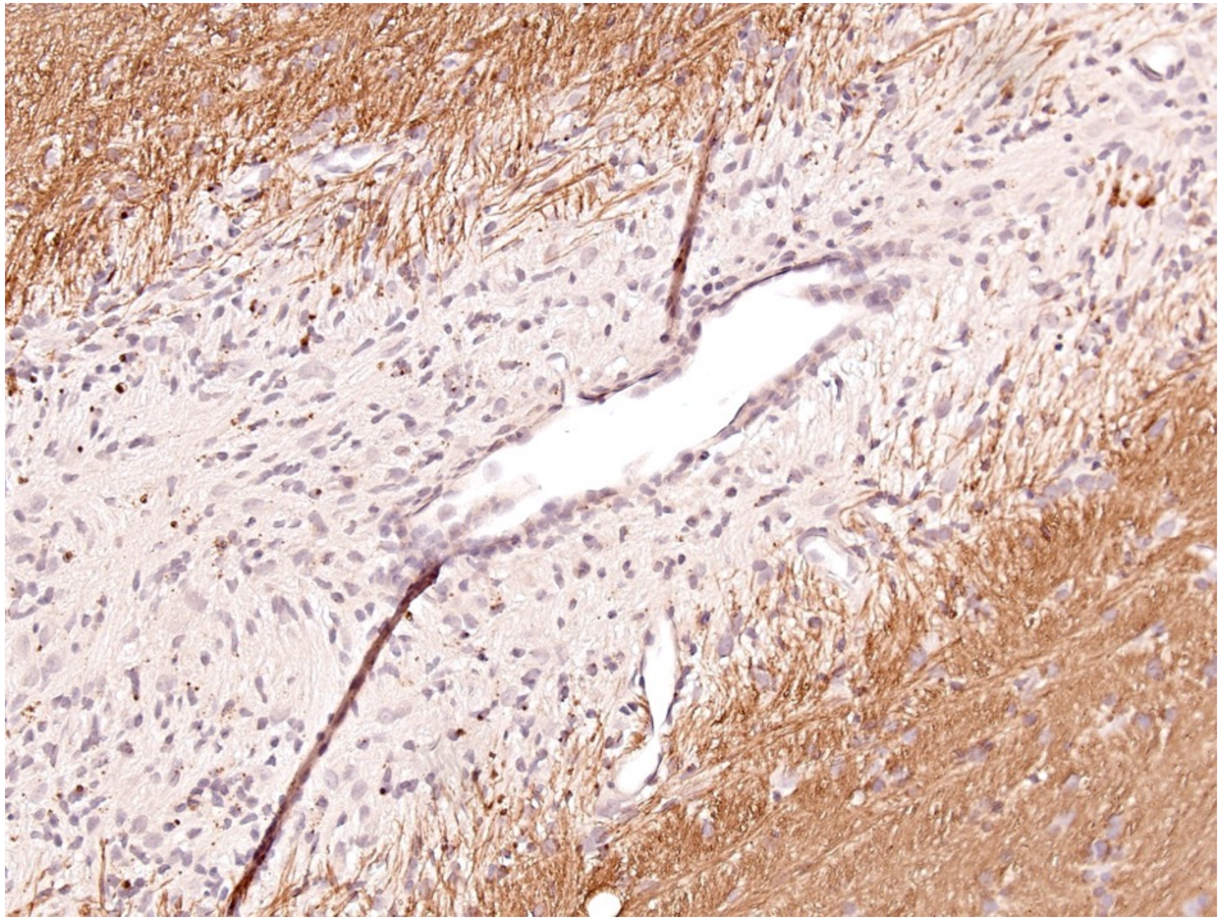

Chronic Demyelinated

Slide unavailable / damaged

Remyelinated

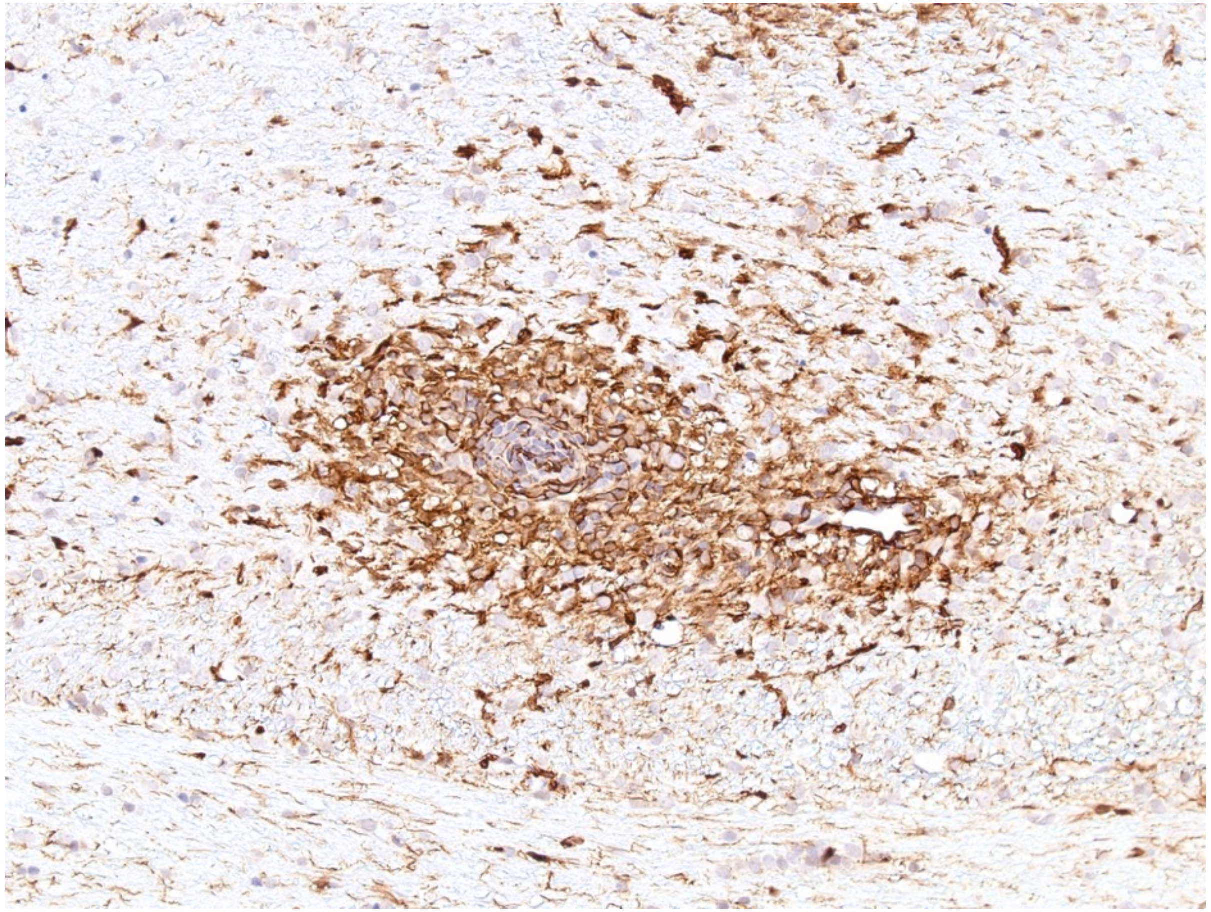

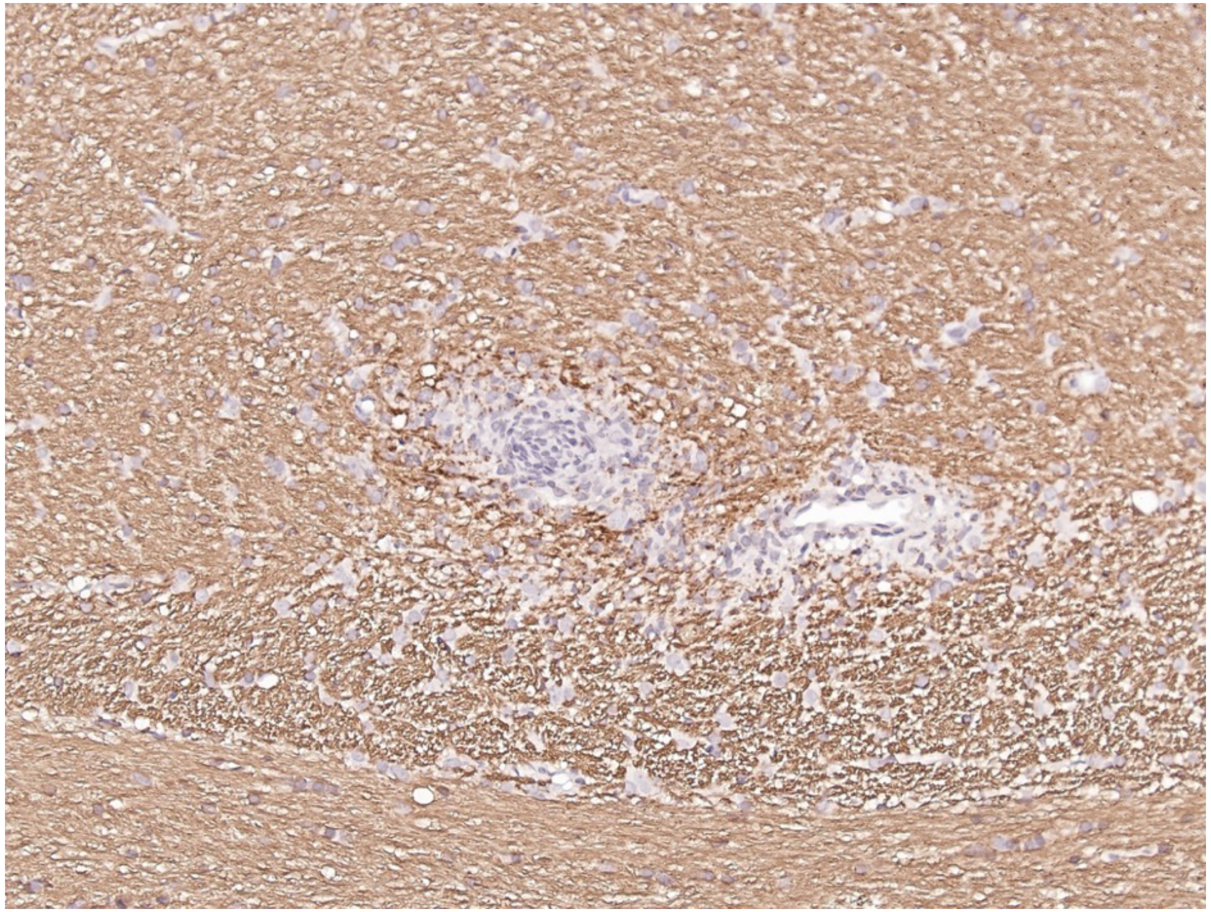

M#6

- 5 Acute Demyelinating
- 3 Chronic Demyelinated

Acute Demyelinating

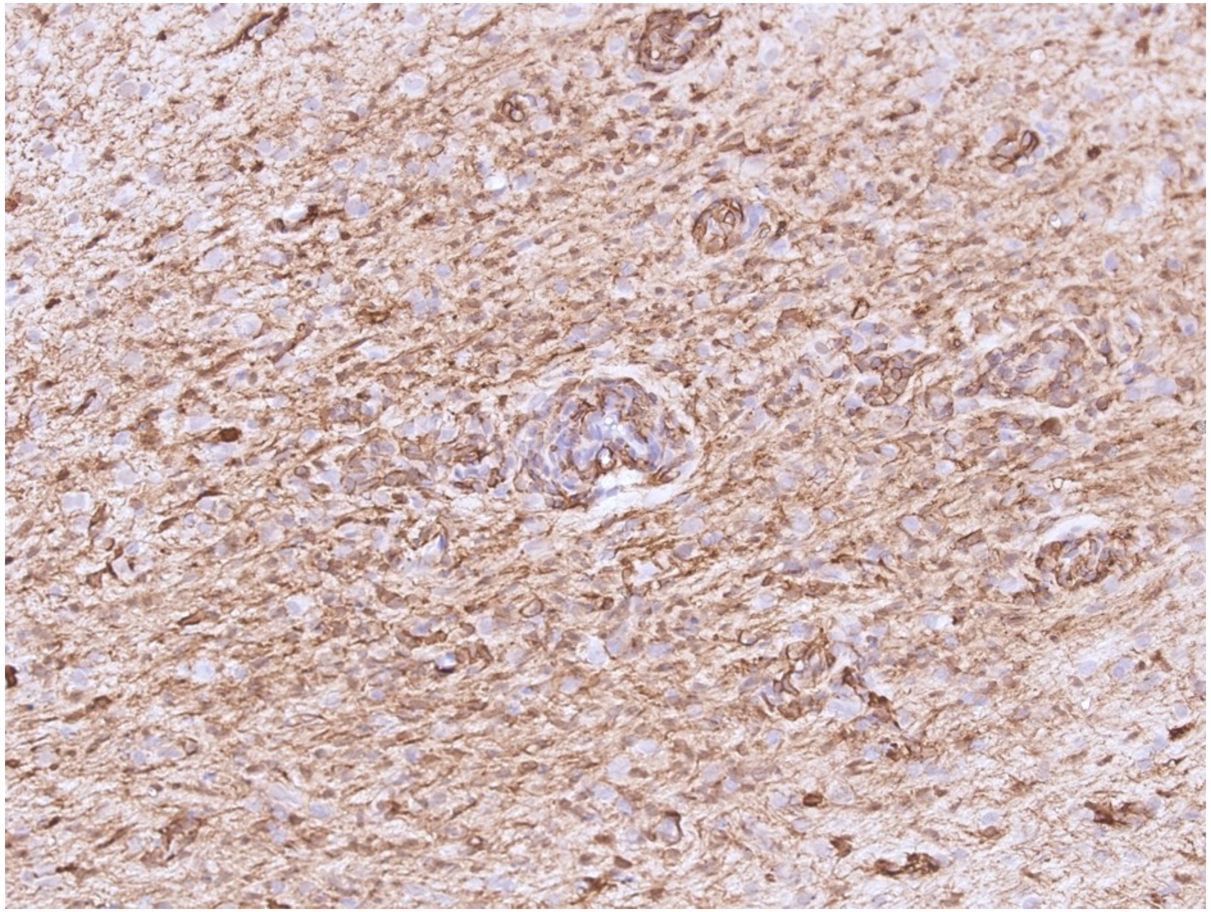

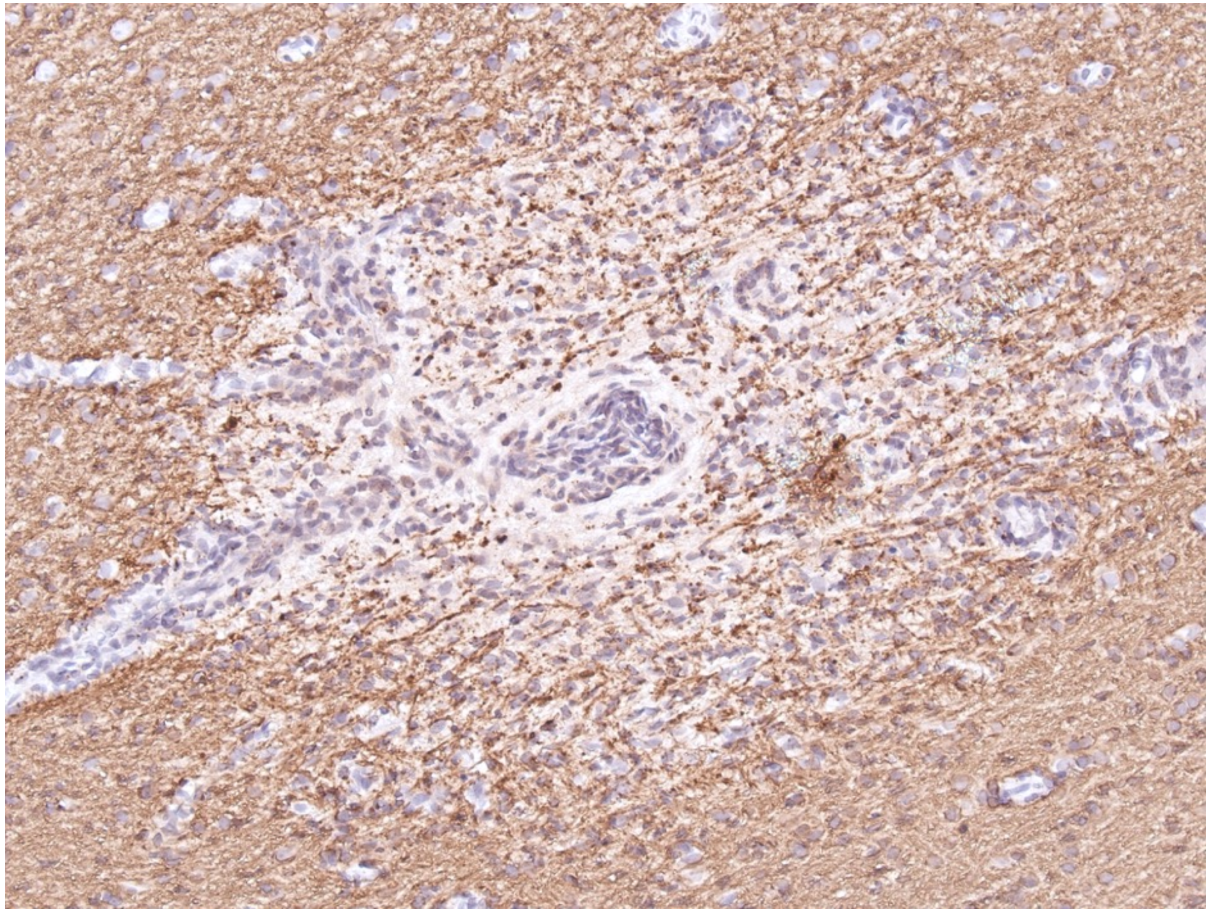

Acute Demyelinating

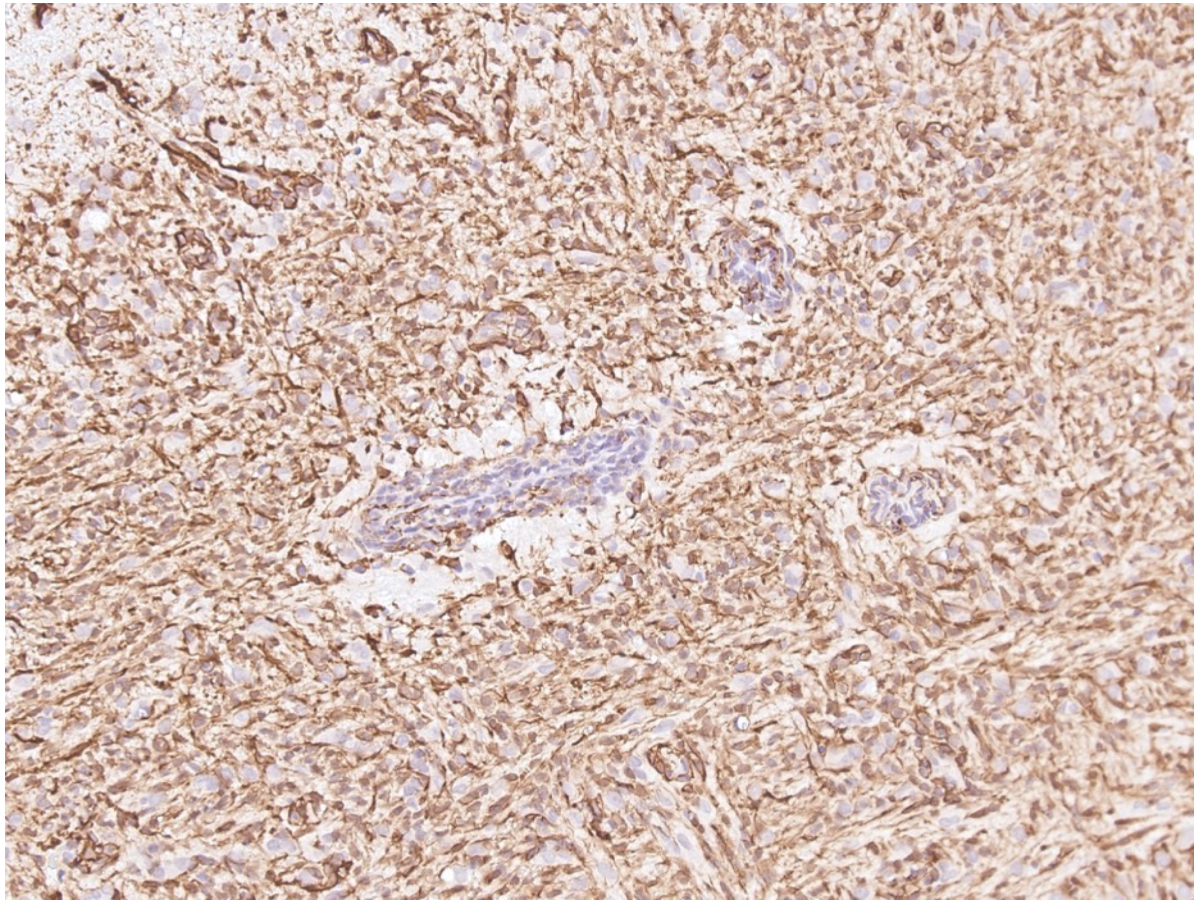

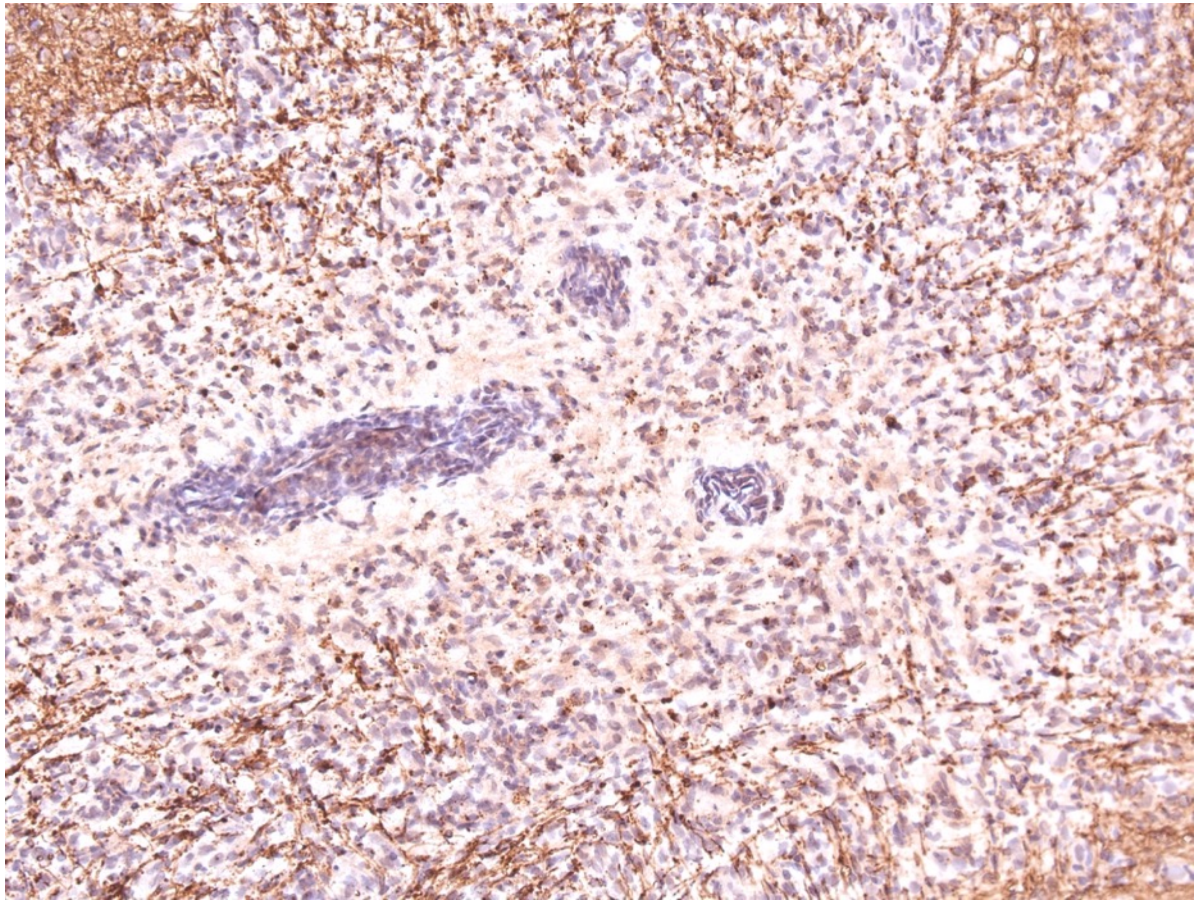

Acute Demyelinating

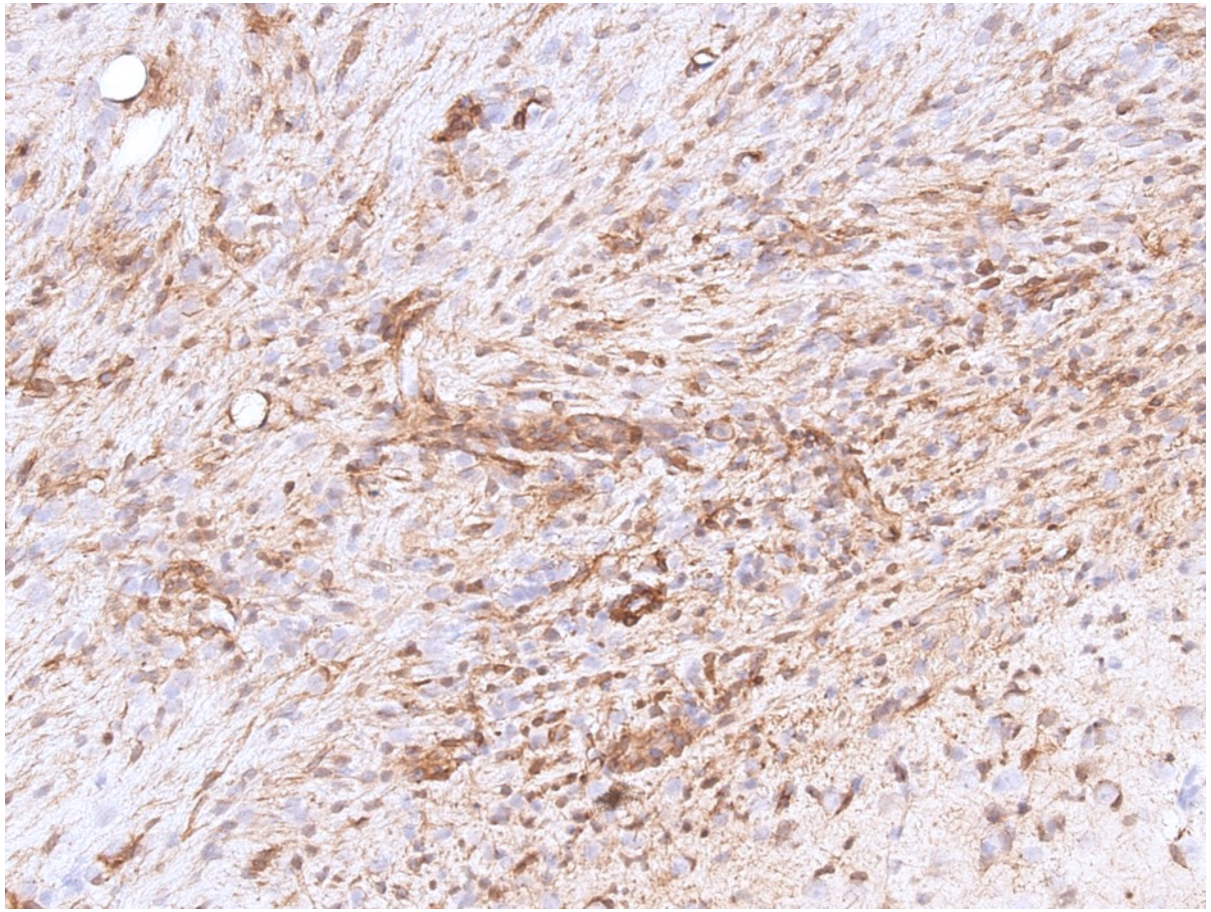

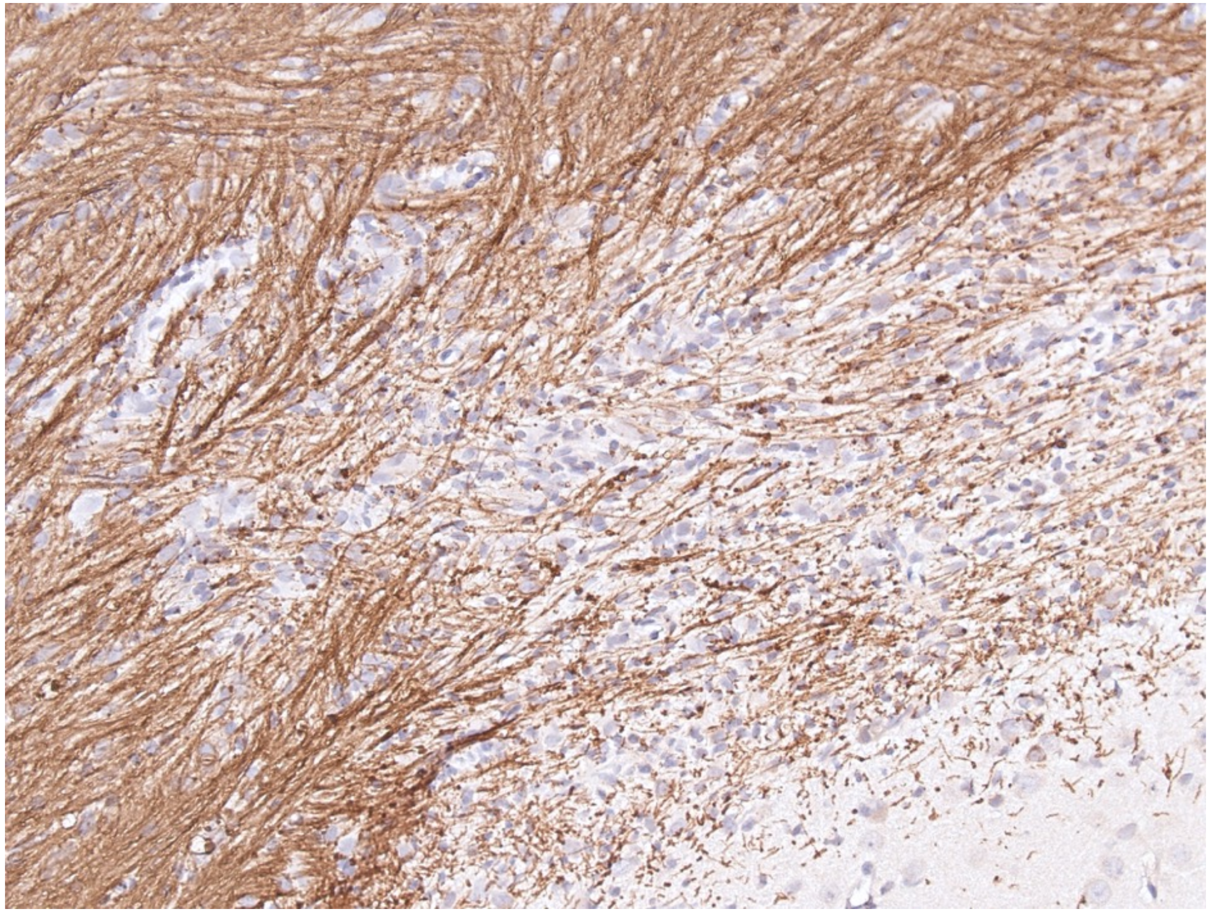

Acute Demyelinating

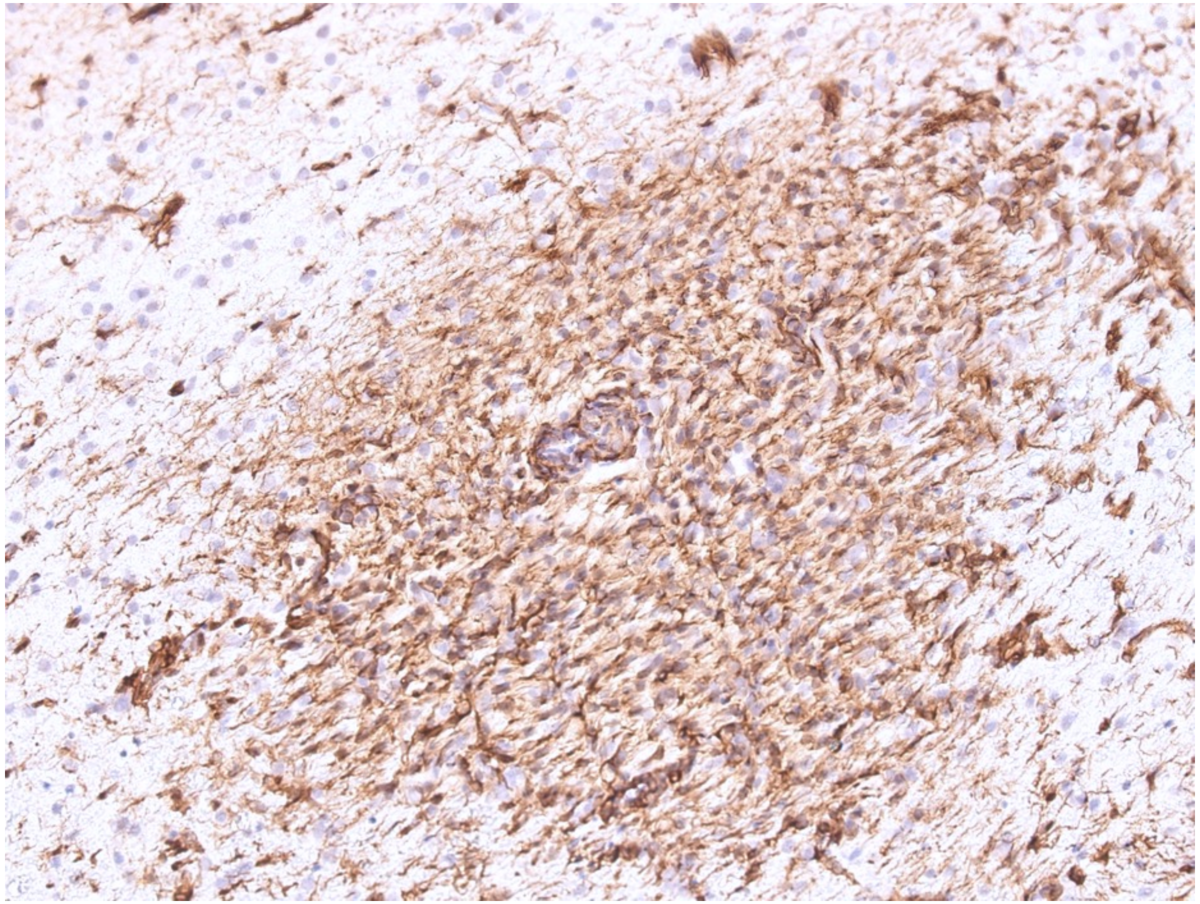

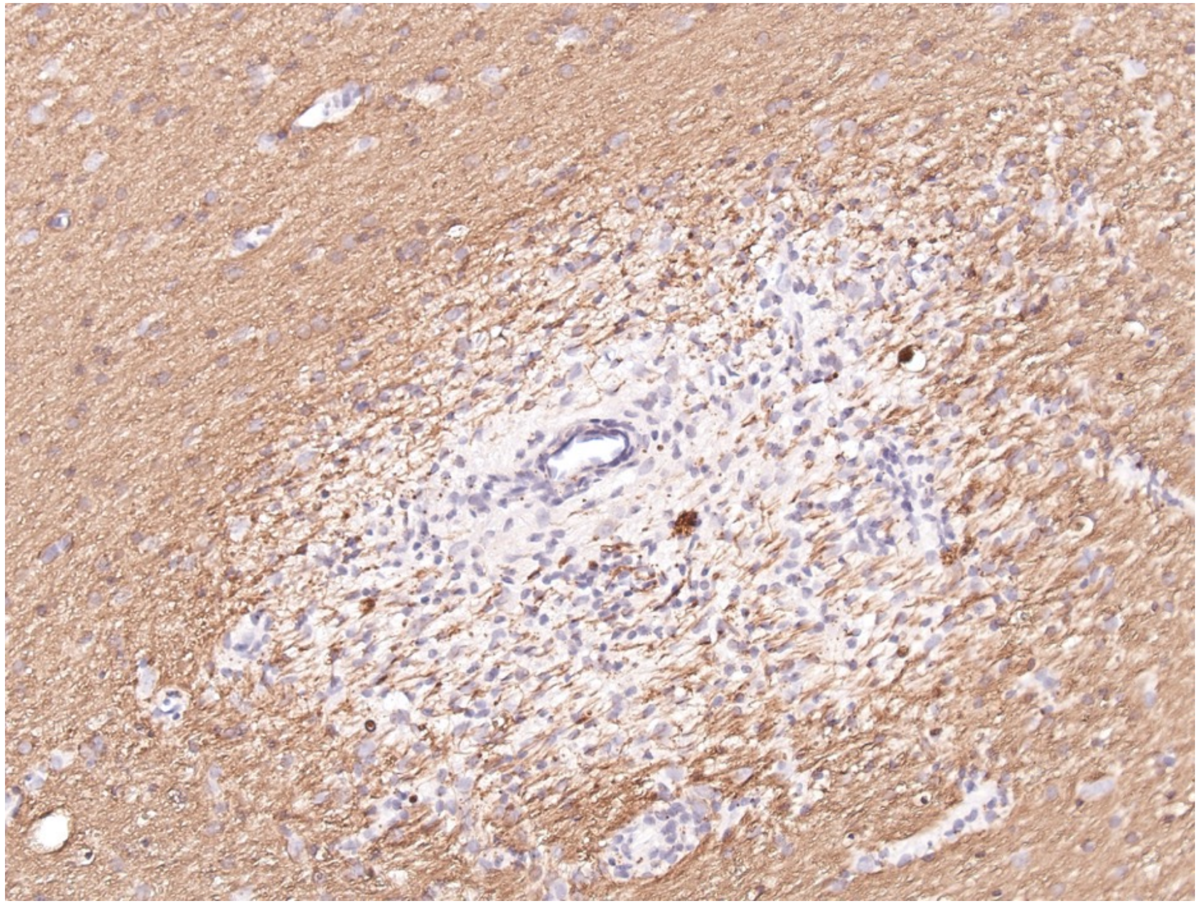

Acute Demyelinating

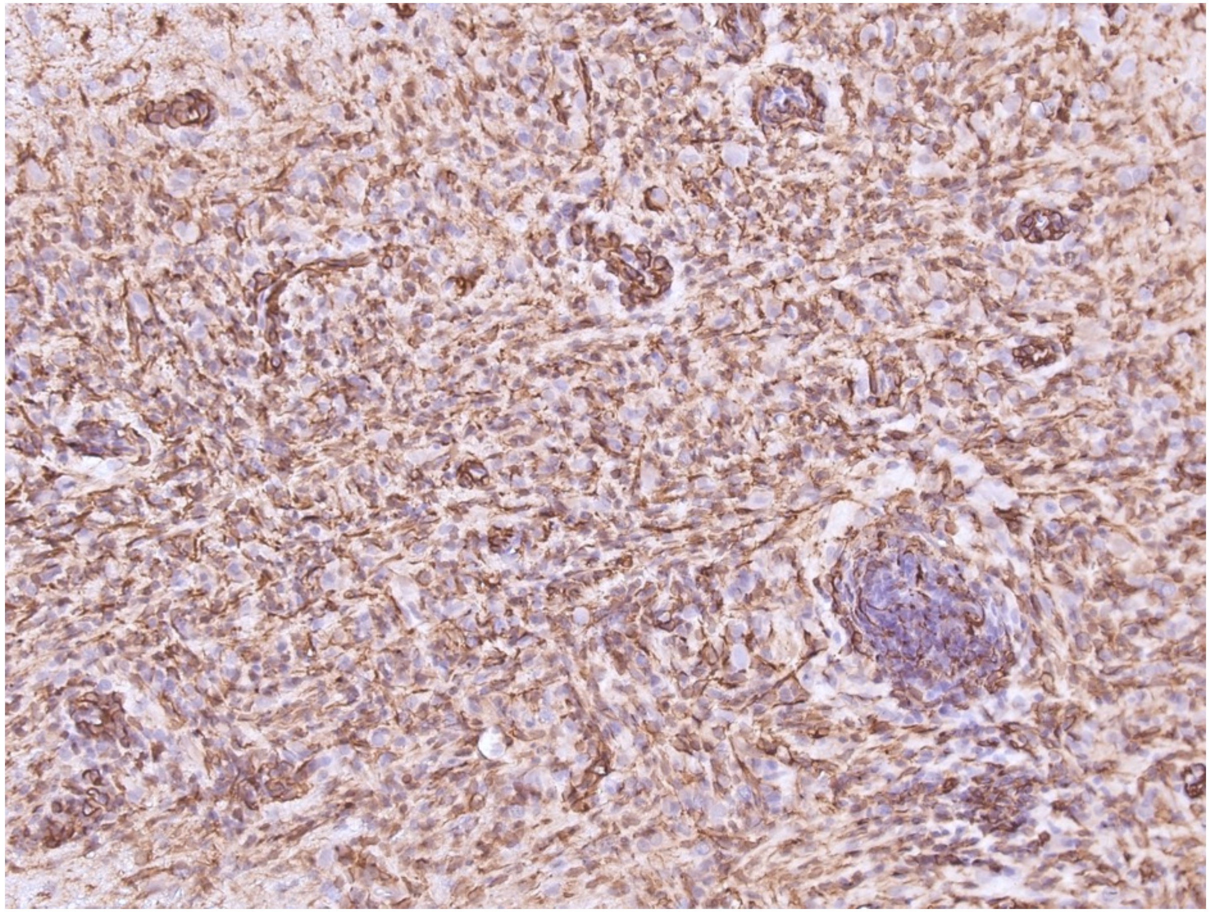

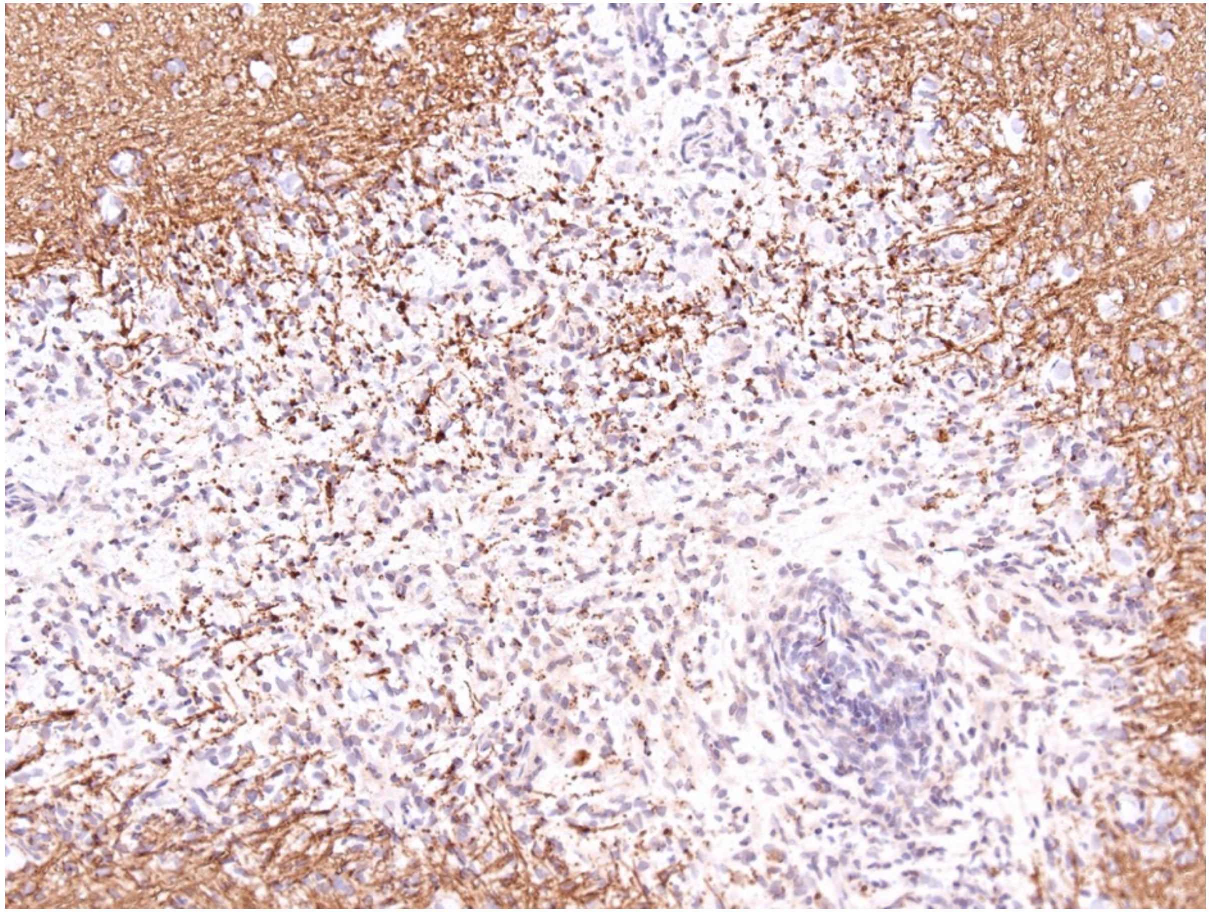

Chronic Demyelinated

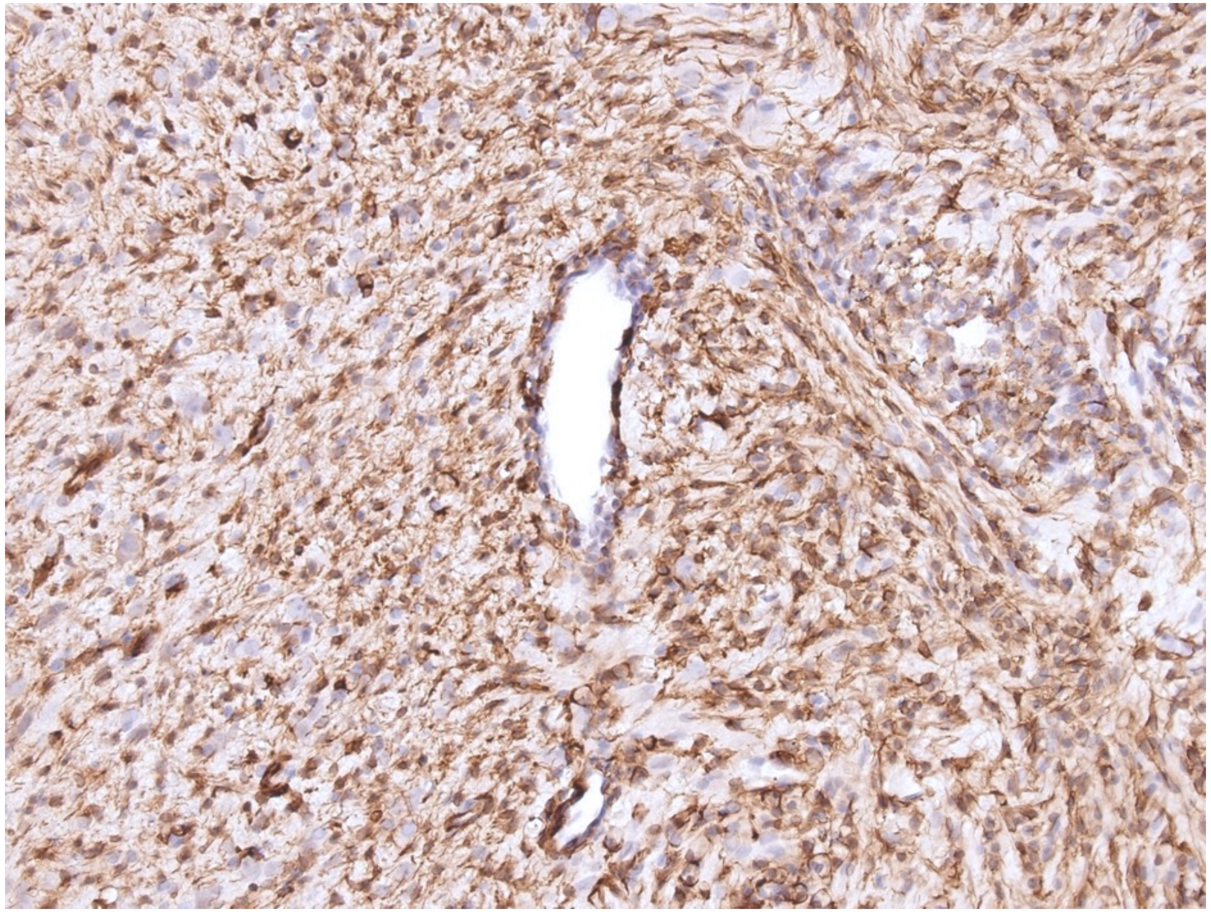

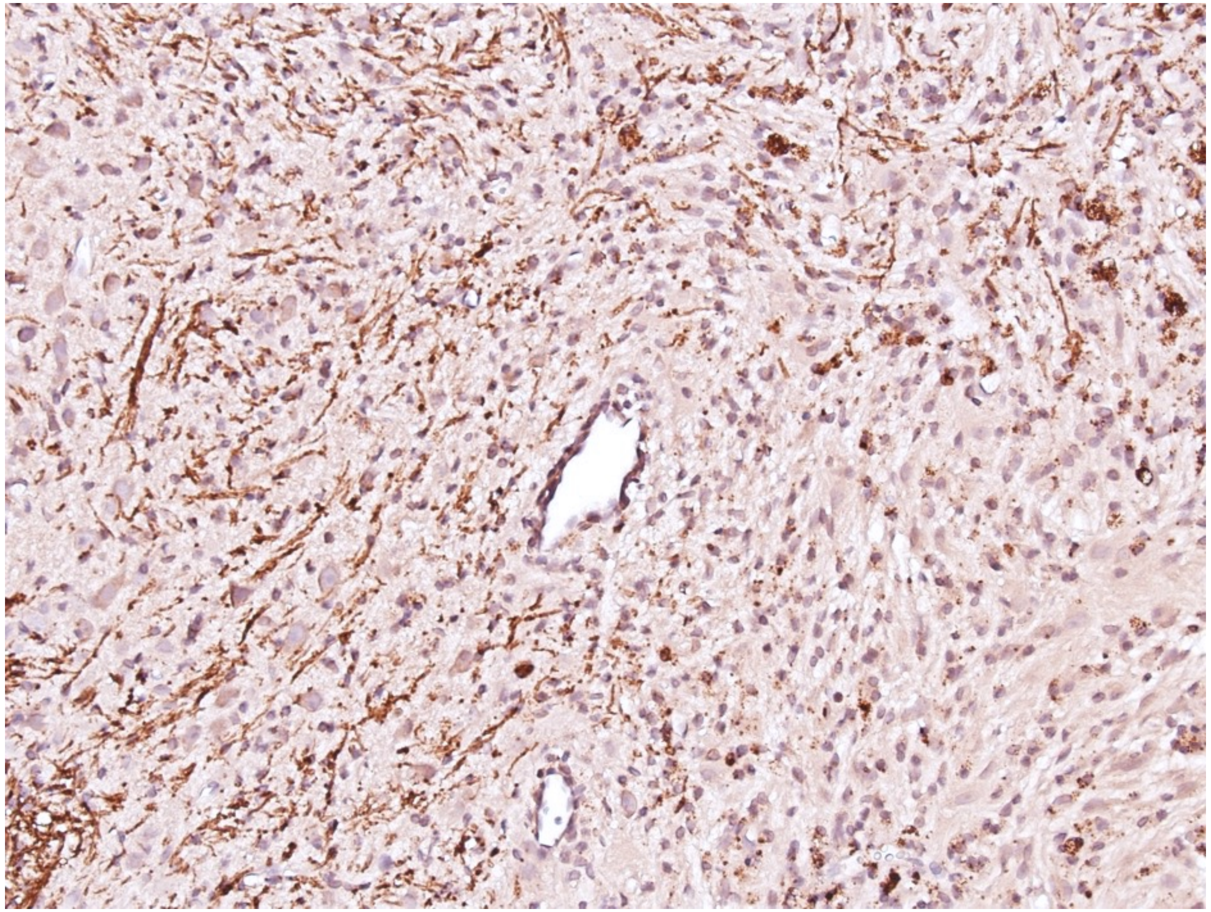

Chronic Demyelinated

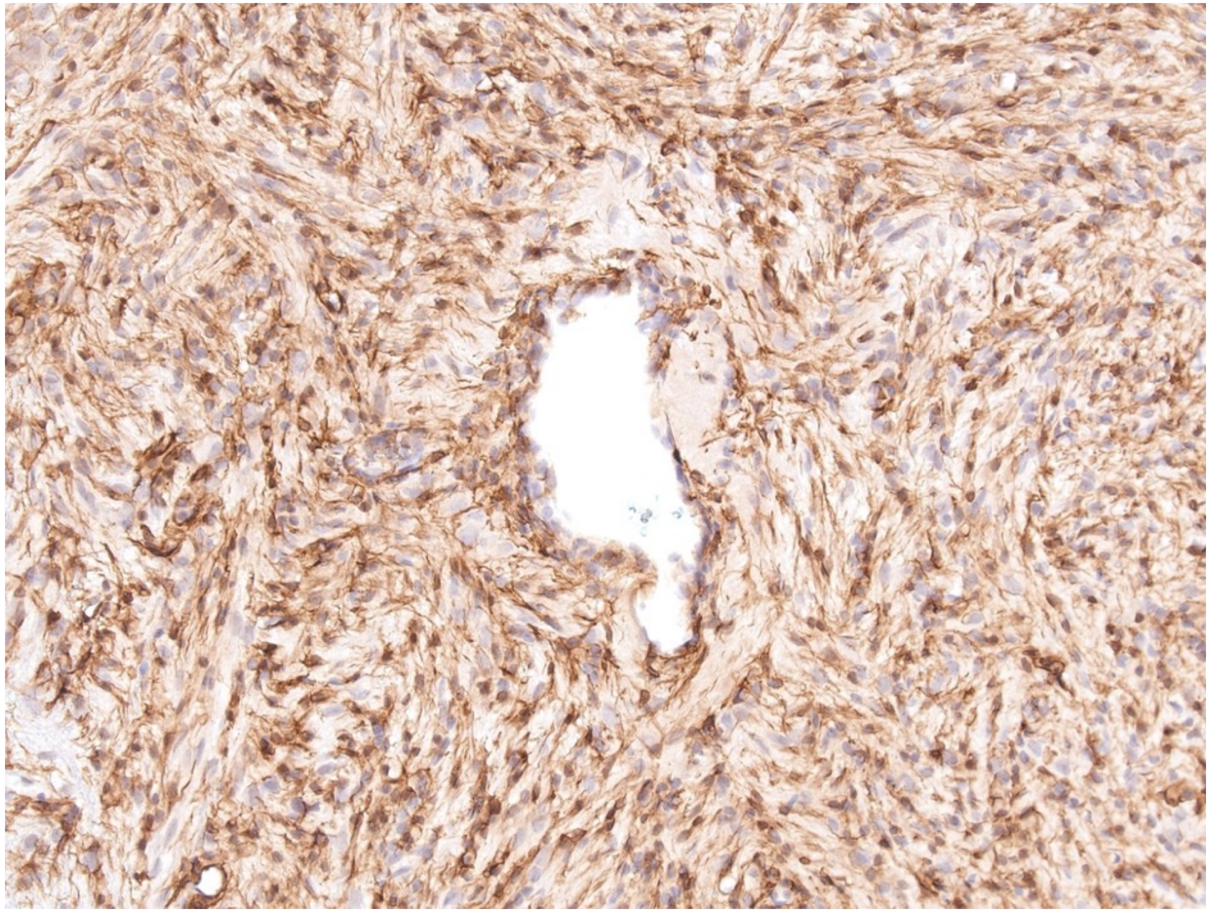

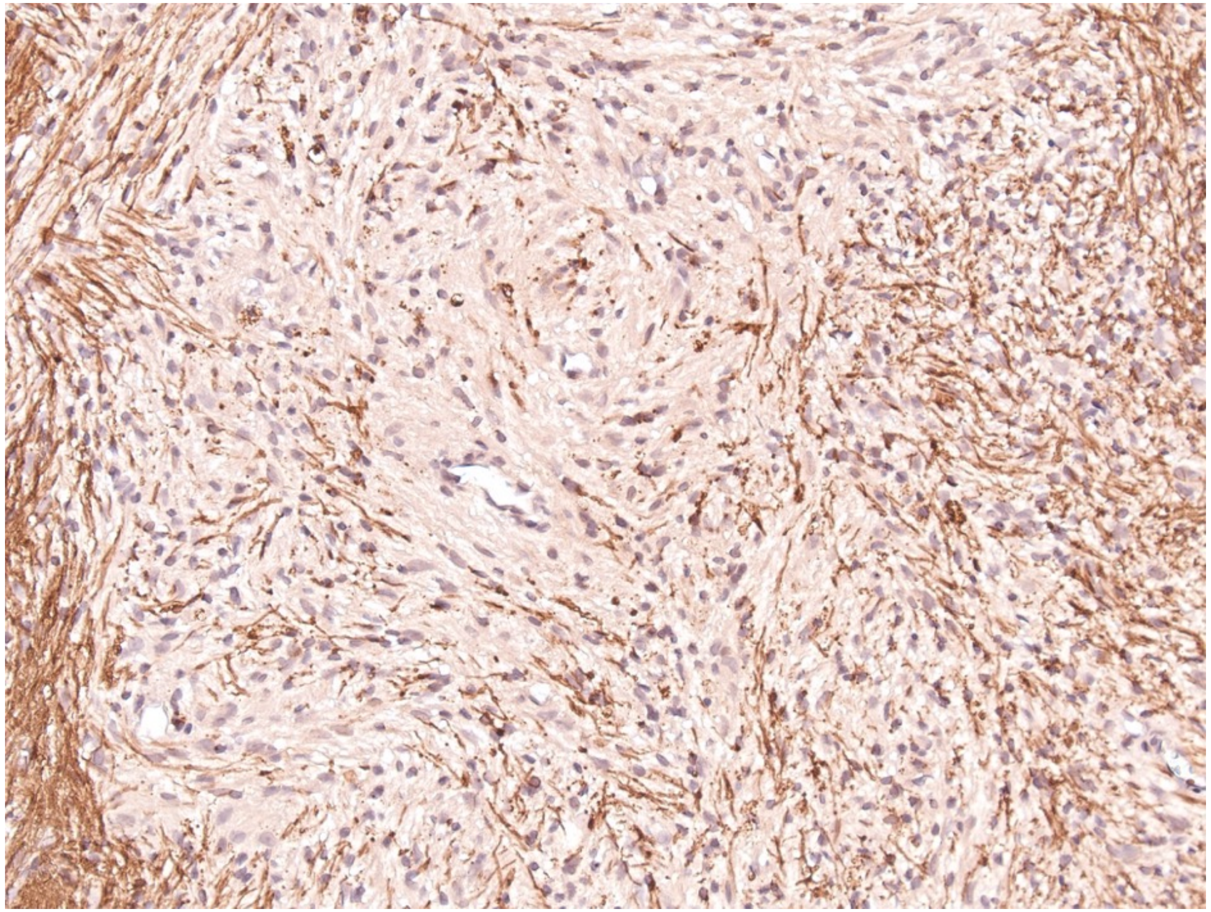

Chronic Demyelinated

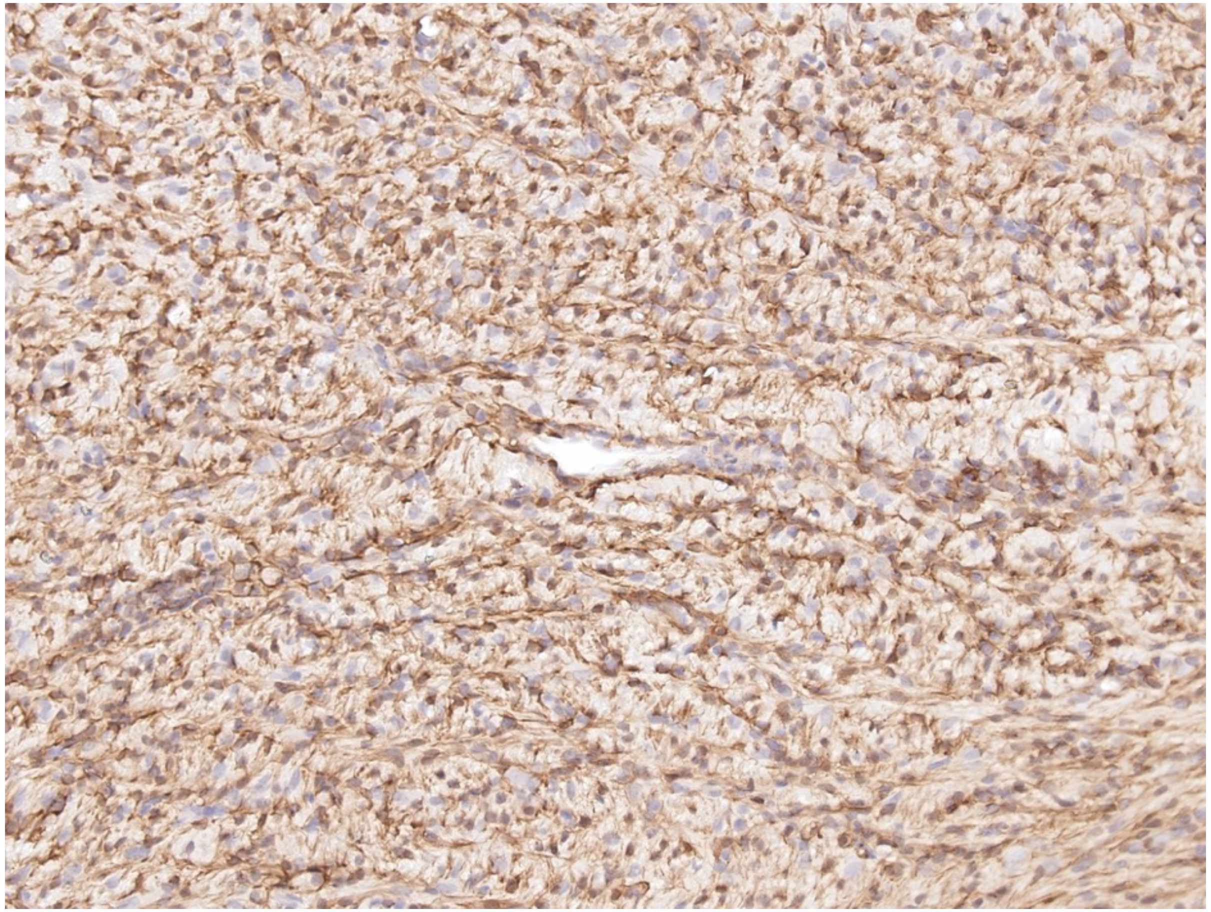

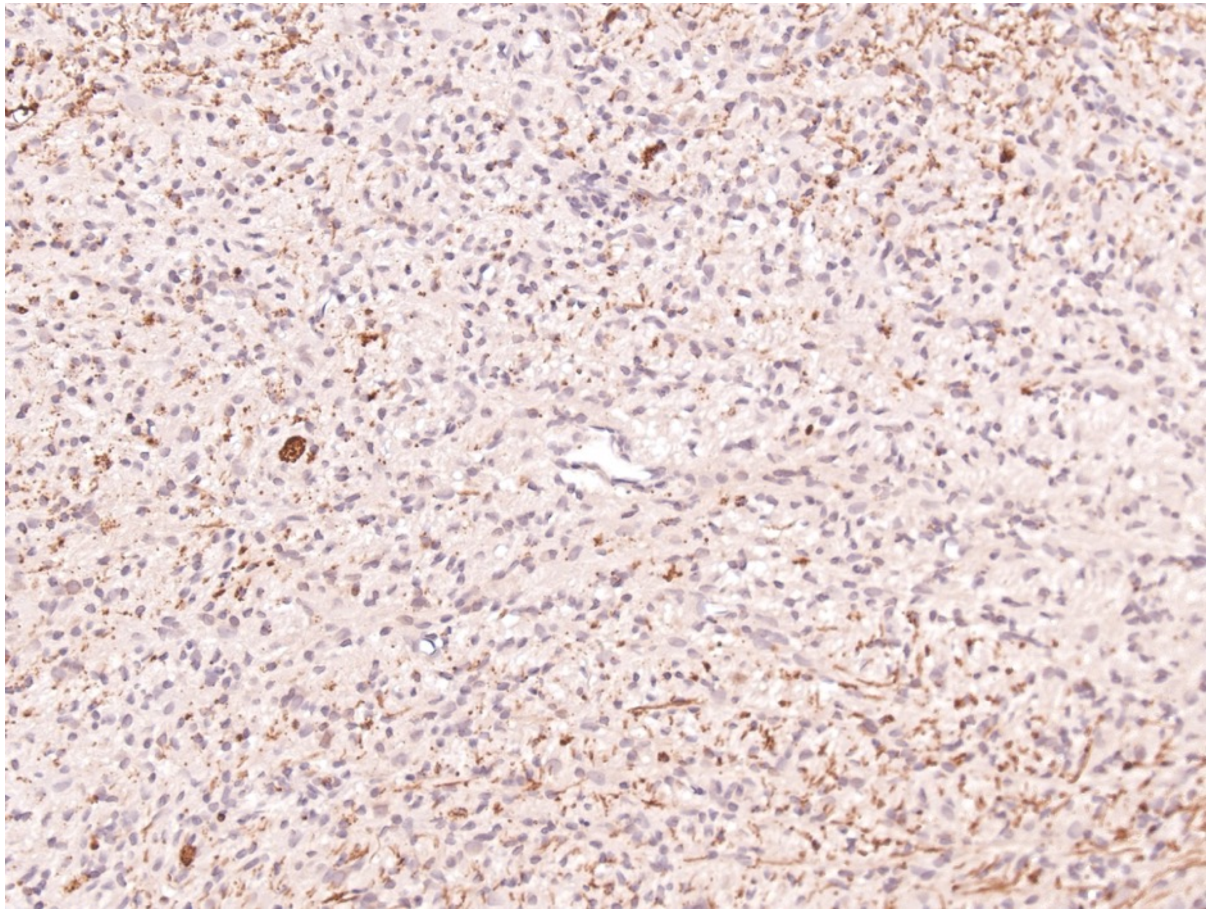

Supplement: Source data 9. [file elife-73786-data9.pdf]
