# Supplementary material for: In vivo MRI is sensitive to remyelination in a nonhuman primate model of multiple sclerosis
Source: eLife. 2023 Apr 21;12:e73786. doi: 10.7554/eLife.73786 (PMC10171859; doi:10.7554/eLife.73786)
Supplement: Transparent reporting form [file elife-73786-transrepform1.docx]

***eLife’s* transparent reporting form**

We encourage authors to provide detailed information *within their submission* to facilitate the interpretation and replication of experiments. Authors can upload supporting documentation to indicate the use of appropriate reporting guidelines for health-related research (see [EQUATOR Network](http://www.equator-network.org/%20)), life science research (see the [BioSharing Information Resource](https://biosharing.org/)), or the [ARRIVE guidelines](http://www.plosbiology.org/article/info:doi/10.1371/journal.pbio.1000412) for reporting work involving animal research. Where applicable, authors should refer to any relevant reporting standards documents in this form.

If you have any questions, please consult our Journal Policies and/or contact us: [editorial@elifesciences.org](mailto:editorial@elifesciences.org).

**Sample-size estimation**

- You should state whether an appropriate sample size was computed when the study was being designed
- You should state the statistical method of sample size computation and any required assumptions
- If no explicit power analysis was used, you should describe how you decided what sample (replicate) size (number) to use

Please outline where this information can be found within the submission (e.g., sections or figure legends), or explain why this information doesn’t apply to your submission:

The number of marmosets used for the study was mainly determined based on the availability of the animals in the facility, as marmosets are a scarce resource. No explicit power analysis was used, but since the statistical analyses were based on the lesion level, we believe this will provide enough statistical power to support the conclusions of the current study.

**Replicates**

- You should report how often each experiment was performed
- You should include a definition of biological versus technical replication
- The data obtained should be provided and sufficient information should be provided to indicate the number of independent biological and/or technical replicates
- If you encountered any outliers, you should describe how these were handled
- Criteria for exclusion/inclusion of data should be clearly stated
- High-throughput sequence data should be uploaded before submission, with a private link for reviewers provided (these are available from both GEO and ArrayExpress)

Please outline where this information can be found within the submission (e.g., sections or figure legends), or explain why this information doesn’t apply to your submission:

All of the above information can be found inside the manuscript, under the METHODS section. Briefly, the marmosets were scanned *in vivo* using the 7T MRI dedicated to animal studies on a weekly basis, from the induction of experimental autoimmune encephalomyelitis until the animals were euthanized at the end of the study once the ethical and medical indications were met (also explained in detail under the METHODS section). No outliers were excluded from the study. Inclusion/exclusion criteria for lesions, mainly based on size and clarity on the MRI scans, are also elaborated within the manuscript.

**Statistical reporting**

- Statistical analysis methods should be described and justified
- Raw data should be presented in figures whenever informative to do so (typically when N per group is less than 10)
- For each experiment, you should identify the statistical tests used, exact values of N, definitions of center, methods of multiple test correction, and dispersion and precision measures (e.g., mean, median, SD, SEM, confidence intervals; and, for the major substantive results, a measure of effect size (e.g., Pearson's r, Cohen's d)
- Report exact p-values wherever possible alongside the summary statistics and 95% confidence intervals. These should be reported for all key questions and not only when the p-value is less than 0.05.

Please outline where this information can be found within the submission (e.g., sections or figure legends), or explain why this information doesn’t apply to your submission:

As we have acquired hundreds of serial *in vivo* MRI for each animals, not all raw data were included in the figures, but all the representative lesions went through the same pipeline of image correction, including skull stripping (outlined detailedly on our Supplementary Figure 1). and analysis based on our in-house machine learning algorithm.

(For large datasets, or papers with a very large number of statistical tests, you may upload a single table file with tests, Ns, etc., with reference to sections in the manuscript.)

**Group allocation**

- Indicate how samples were allocated into experimental groups (in the case of clinical studies, please specify allocation to treatment method); if randomization was used, please also state if restricted randomization was applied
- Indicate if masking was used during group allocation, data collection and/or data analysis

Please outline where this information can be found within the submission (e.g., sections or figure legends), or explain why this information doesn’t apply to your submission:

The treatment groups for steroids vs. no steroids was decided based on which animal within the twin pairs developed the lesions first, detected by *in vivo* MRI. While data were analyzed, the name of all the animals, age of lesions, and any information pertinent to the analyses were anonymized. Further information can be found on the METHODS sections.

**Additional data files (“source data”)**

- We encourage you to upload relevant additional data files, such as numerical data that are represented as a graph in a figure, or as a summary table
- Where provided, these should be in the most useful format, and they can be uploaded as “Source data” files linked to a main figure or table
- Include model definition files including the full list of parameters used
- Include code used for data analysis (e.g., R, MatLab)
- Avoid stating that data files are “available upon request”

Please indicate the figures or tables for which source data files have been provided:

All of the 6 marmosets’ serial *in vivo* MRI images, including all the sequences used for analysis and figure generation, were uploaded in an easily accessible format (NIFTI). The file names are titled with the corresponding animal # used in the manuscript, as well as the date of MRI acquisition.
